# Synthesis, Molecular Structure, Anticancer Activity, and QSAR Study of *N*-(aryl/heteroaryl)-4-(1*H*-pyrrol-1-yl)Benzenesulfonamide Derivatives

**DOI:** 10.3390/ijms19051482

**Published:** 2018-05-16

**Authors:** Beata Żołnowska, Jarosław Sławiński, Zdzisław Brzozowski, Anna Kawiak, Mariusz Belka, Joanna Zielińska, Tomasz Bączek, Jarosław Chojnacki

**Affiliations:** 1 Department of Organic Chemistry, Medical University of Gdansk, Al. Gen. J. Hallera 107, 80-416 Gdansk, Poland; brzozowskigumed@gmail.com; 2 Department of Biotechnology, Intercollegiate Faculty of Biotechnology, University of Gdansk and Medical University of Gdansk, ul. Abrahama 58, 80-307 Gdansk, Poland; kawiak@biotech.ug.edu.pl; 3 Laboratory of Human Physiology, Medical University of Gdansk, ul. Tuwima 15, 80-210 Gdansk, Poland; 4 Department of Pharmaceutical Chemistry, Medical University of Gdansk, Al. Gen. J. Hallera 107, 80-416 Gdansk, Poland; mariusz.belka@gumed.edu.pl (M.B.); joanna.zielinska@gumed.edu.pl (J.Z.); tomasz.baczek@gumed.edu.pl (T.B.); 5 Department of Inorganic Chemistry, Gdansk University of Technology, ul. Narutowicza 11/12, 80-233 Gdansk, Poland; jaroslaw.chojnacki@pg.edu.pl

**Keywords:** benzenesulfonamide, synthesis, anticancer, apoptosis, OPLS

## Abstract

A series of *N*-(aryl/heteroaryl)-4-(1*H*-pyrrol-1-yl)benzenesulfonamides were synthesized from 4-amino-*N*-(aryl/heteroaryl)benzenesulfonamides and 2,5-dimethoxytetrahydrofuran. All the synthesized compounds were evaluated for their anticancer activity on HeLa, HCT-116, and MCF-7 human tumor cell lines. Compound **28**, bearing 8-quinolinyl moiety, exhibited the most potent anticancer activity against the HCT-116, MCF-7, and HeLa cell lines, with IC_50_ values of 3, 5, and 7 µM, respectively. The apoptotic potential of the most active compound (**28**) was analyzed through various assays: phosphatidylserine translocation, cell cycle distribution, and caspase activation. Compound **28** promoted cell cycle arrest in G2/M phase in cancer cells, induced caspase activity, and increased the population of apoptotic cells. Relationships between structure and biological activity were determined by the QSAR (quantitative structure activity relationships) method. Analysis of quantitative structure activity relationships allowed us to generate OPLS (Orthogonal Projections to Latent Structure) models with verified predictive ability that point out key molecular descriptors influencing benzenosulfonamide’s activity.

## 1. Introduction

Nowadays, cancer causes one in seven deaths worldwide, and has become more fatal than AIDS (acquired immune deficiency syndrome), tuberculosis, and malaria combined. As was estimated by the International Agency for Research on Cancer, total cancer deaths in 2012 reached 8.2 million and it is expected that, by 2030 cancer deaths will surpass 13 million per year due to the growth and aging of the population [1]. 

According to statistics, in 2012 there were an estimated 1.4 million new colorectal cancer cases and 693,900 deaths. The incidence rates in both males and females were higher in high-income countries than low- and middle-income countries (LMICs). Breast cancer, the leading cause of cancer-related death among females worldwide, resulted in an estimated 1.7 million cases and 521,900 deaths in 2012. An estimated 527,600 cancer cases and 265,700 deaths in 2012 worldwide were caused by cervical cancer, which is the third leading cause of cancer-related death in females in LMICs [2]. 

Chemotherapeutics play an important role as anticancer agents, inducing apoptosis or restoring the apoptotic functions of proteins. Apoptosis is programmed cell death that leads to the elimination of unwanted cells [3]. In many cancers, the apoptotic process is deregulated because of inactivating mutations in genes encoding pro-apoptotic proteins or through upregulation of anti-apoptotic proteins, leading to the uncontrolled growth of tumors [4]. Apoptotic cancer cell death can occur by inducing upregulation of pro-apoptotic proteins or by directly decreasing the anti-apoptotic reservoir. 

**Figure 1:**
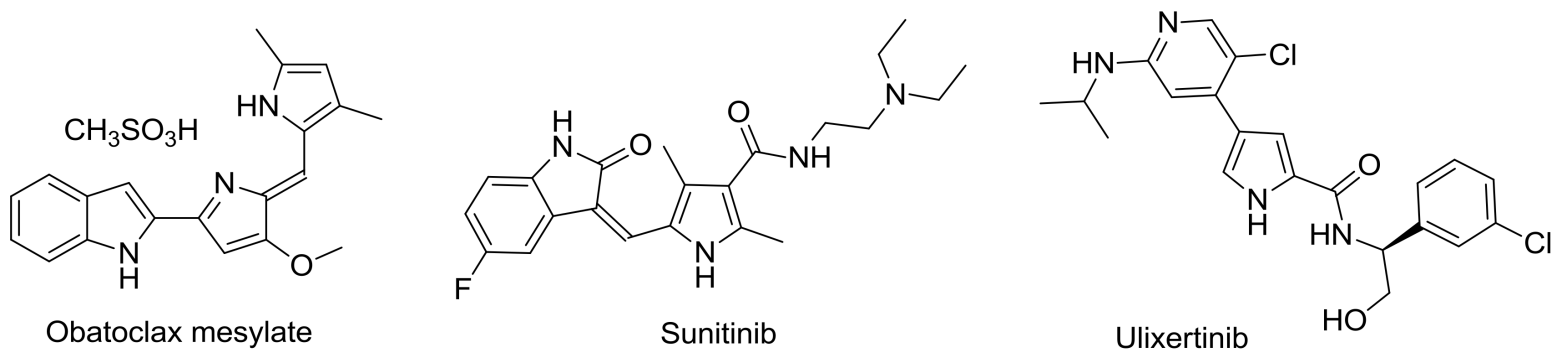
The structures of obatoclax mesylate, sunitinib, and ulixertinib.

Pyrrole has become a biologically important, nitrogen-containing heterocyclic object since its presence in many natural products—including heme, vitamin B12, bile pigments, and alkaloids—was discovered [5,6]. Diverse therapeutic applications of pyrrole derivatives as fungicides, antibiotics, anti-inflammatories, anti-tubercular agents, cholesterol reducing drugs, and antitumor agents have been widely described [6,7]. It is well known that some pyrroles belong to a class of promising anticancer agents, including obatoclax, sunitinib, and ulixertinib (Figure 1). Obatoclax was identified as a Bcl-2 family inhibitor, and has been evaluated preclinically and clinically in both hematological malignancies and solid tumors [8]. Sunitinib is an oral multi-targeted tyrosine kinase inhibitor, which has been approved in the first-line treatment of metastatic renal cell cancer [9]. Ulixertinib, an ERK1/2 kinase inhibitor, is used to treat cancer patients with MAPK mutant solid tumors [10]. 

**Figure 2:**
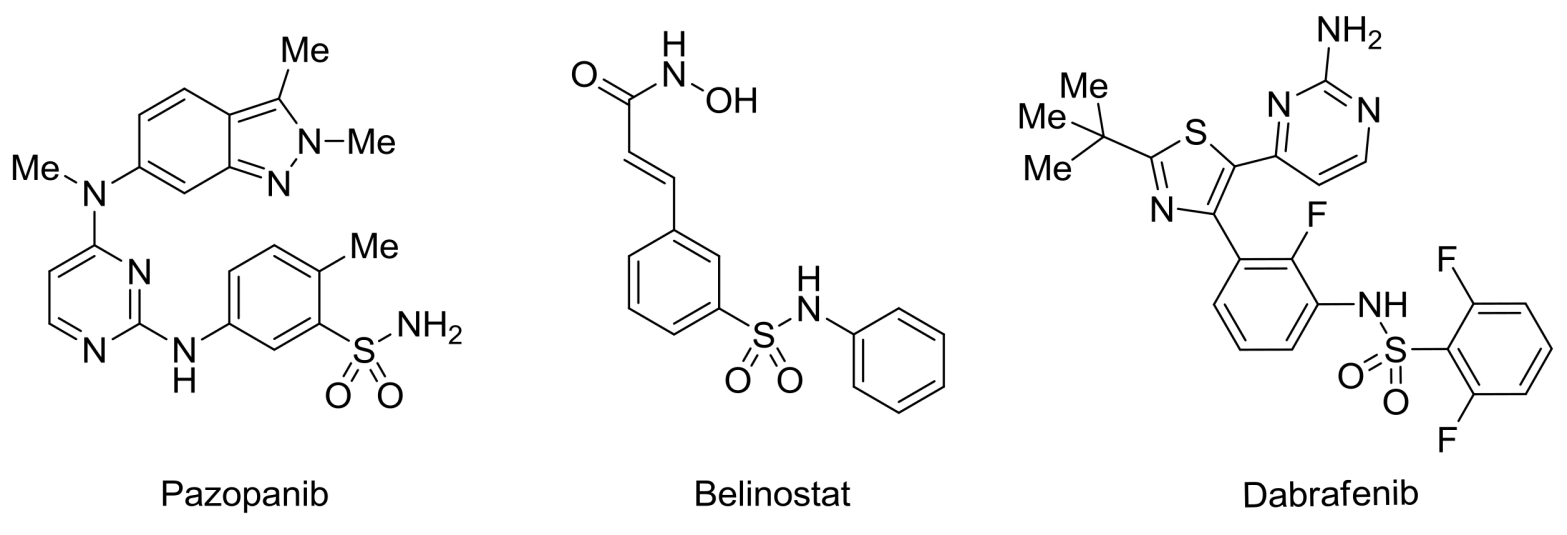
Sulfonamides used as antitumor drugs.

Among the variety of biological applications of sulfonamides, anticancer activity has attracted special attention from researchers over the last few years. A large number of sulfonamide derivatives show a variety of mechanisms of anticancer activity, including carbonic anhydrase inhibition, cell cycle perturbation in the G1 phase, tubulin polymerization inhibition, functional suppression of the transcriptional activator NF-Y (nuclear transcription factor Y) and angiogenesis (matrix metalloproteinase) inhibition [11]. Moreover, some sulfonamide drugs such as pazopanib, belinostat, and dabrafenib have been reported as antitumor agents (Figure 2). Pazopanib is known as a potent and selective multi-targeted receptor tyrosine kinase inhibitor and has been approved for renal cell carcinoma and soft tissue sarcoma [12,13]. Belinostat, as a histone deacetylase inhibitor, has been directed to treat peripheral T-cell lymphoma [14]. The BRAF inhibitor, dabrafenib, has been granted approval for the treatment of unresectable or metastatic melanoma [15] (Figure 2).

Furthermore, our latest research has proven the potential of benzenesulfonamides as anticancer agents, described in the literature as a series of novel *N*-substituted *N*′-[2-arylmethylthio-4-chloro-5-methylbenzenesulfonyl)guanidines [16], *N*-acylbenzenesulfonamides [17], *N*-(5-aryl-1,2,4-triazin-3-yl)benzenesulfonamides [18], 2-benzylthio-5-(1,3,4-oxadiazol-2-yl)benzenesulfonamides [19], *N*-(5-oxo-4,5-dihydro-1,2,4-triazin-3-yl)benzenesulfonamides [20], and 2-(2-alkylthiobenzenesulfonyl)-3-(phenylprop-2-ynylideneamino)guanidines [21]. 

In view of the importance of sulfonamides and nitrogen-containing heterocycles as privileged structures for the design of anticancer agents, we decided to explore the synthesis and anticancer activity of molecular hybrids obtained by the combination of benzenesulfonamide and pyrrole fragments. These compounds were evaluated for their cytotoxicity against three human cancer cell lines: HeLa (cervical cancer), HCT-116 (colon cancer), and MCF-7 (breast cancer). For the most active compound, apoptosis-inducing activity toward cancer cell lines was further investigated. Analysis of the quantitative structure activity relationships (QSAR) allowed us to generate OPLS models (Orthogonal Projections to Latent Structure) with verified predictive ability that defined the key descriptors affecting the *N*-(aryl/heteroaryl)-4-(1*H*-pyrrol-1-yl)benzenesulfonamide’s activity.

## 2. Results and Discussion

### 2.1. Chemistry

**Scheme 1:**
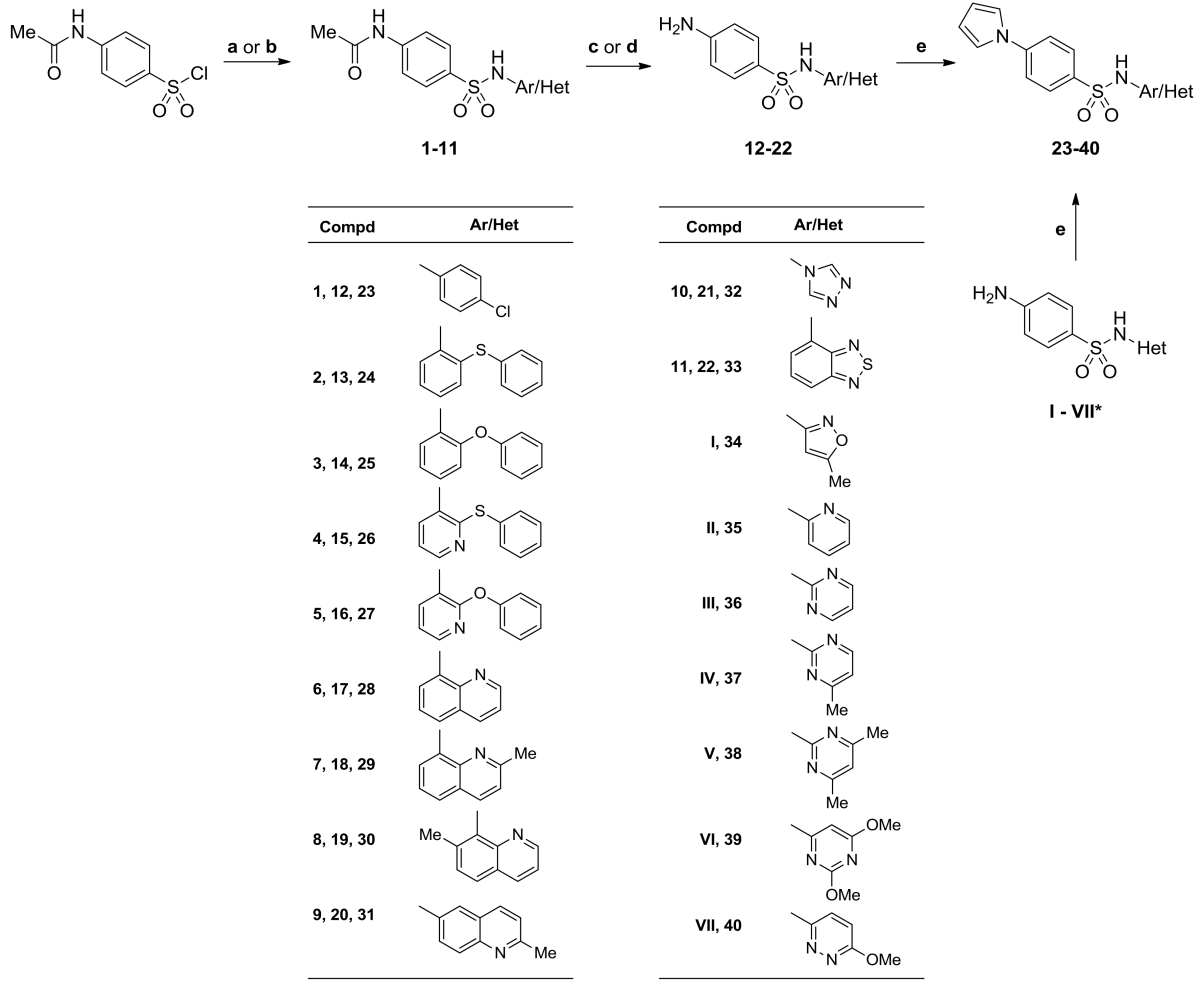
Synthesis of 4-(1*H*-pyrrol-1-yl)-*N*-(aryl/heteroaryl)benzenesulfonamides. (**a**) Ar/Het-NH_2_, dry pyridine, r.t. or 105 °C, 2‒20 h; (**b**) Ar/Het-NH_2_, dry acetone, dry pyridine, r.t., 18‒48 h; (**c**) 8% NaOH in H_2_O, ethanol, 100 °C, 1 h; (**d**) HCl aq, ethanol, 100 °C, 1 h; (**e**) 2,5-dimethoxytetrahydrofuran, *p*-dioxane, glacial acetic acid, reflux, 24‒26 h; Ar = aryl, Het = heteroaryl; * sulfamethoxazole (**I**), sulfapyridine (**II**), sulfadiazine (**III**), sulfamerazine (**IV**), sulfamethazine (**V**), sulfadimethoxine (**VI**), sulfamethoxypyridazine (**VII**).

A series of final *N*-(aryl/heteroaryl)-4-(1*H*-pyrrol-1-yl)benzenesulfonamide derivatives **23**–**40** was synthesized by the reaction of the appropriate 4-amino-*N*-(aryl/heteroaryl)benzenesulfonamides (**I**–**VII**, **12**–**22**) with 2,5-dimethoxytetrahydrofuran in a mixture of *p*-dioxane and glacial acetic acid for 24–26 h at reflux (Scheme 1). Substrates **I**–**VII**, known as antibacterial drugs (sulfamethoxazole, sulfapyridine, sulfadiazine, sulfamerazine, sulfamethazine, sulfadimethoxine, and sulfamethoxypyridazine), were commercially available. In contrast, substrates **12**–**22** were obtained in a two-step process, as shown in Scheme 1. The first step relied on *N*-sulfonation of aryl/heteroarylamine in the presence of dry pyridine or in a mixture of dry acetone and pyridine (*v*/*v* = 2:1). In the second step, a hydrolysis of 4-acetamido-*N*-(aryl/heteroaryl)benzenesulfonamide derivatives **1**–**11** was performed, giving the desired 4-amino-*N*-(aryl/heteroaryl)benzenesulfonamides **12**–**22**.

IR, ^1^H NMR, and ^13^C NMR spectroscopy confirmed the structures of the compounds. Additionally, **25** an X-ray analysis was undertaken. 4-Acetamido-*N*-(aryl/heteroaryl)benzenesulfonamides presented in the IR spectra typical absorption bands at 1662‒1692 cm^−1^, associated with C=O bond stretching. In the ^1^H NMR spectra it showed singlets at approximately 2 ppm corresponding to an acetyl group and two NH singlets at about 10 ppm (see the Supplementary Material). An acetyl group has been also recognized as a peak at approximately 170 ppm in the ^13^C NMR spectra (see the Supplementary Material). Hydrolysis of 4-acetamido-*N*-(aryl/heteroaryl)benzenesulfonamides caused the appearance of NH_2_ stretching, as well as bending bands at 3498‒3008 cm^−1^ and 1658‒1620 cm^−1^, respectively. On the other hand, the primary amine group of 4-amino-*N*-(aryl/heteroaryl)benzenesulfonamides has been identified in the ^1^H NMR spectra as a singlet in the 5.79‒6.00 ppm range. Finally, 4-(1*H*-pyrrol-1-yl)-*N*-(aryl/heteroaryl)benzenesulfonamides showed in IR intense absorption bands at 1516‒1506 cm^−1^ derived from the C=N bond, and distinctive pyrrole signals (broad singlet or triplet) at 6.26‒6.35 ppm and 7.41‒7.68 ppm in ^1^H NMR. 

**Figure 3:**
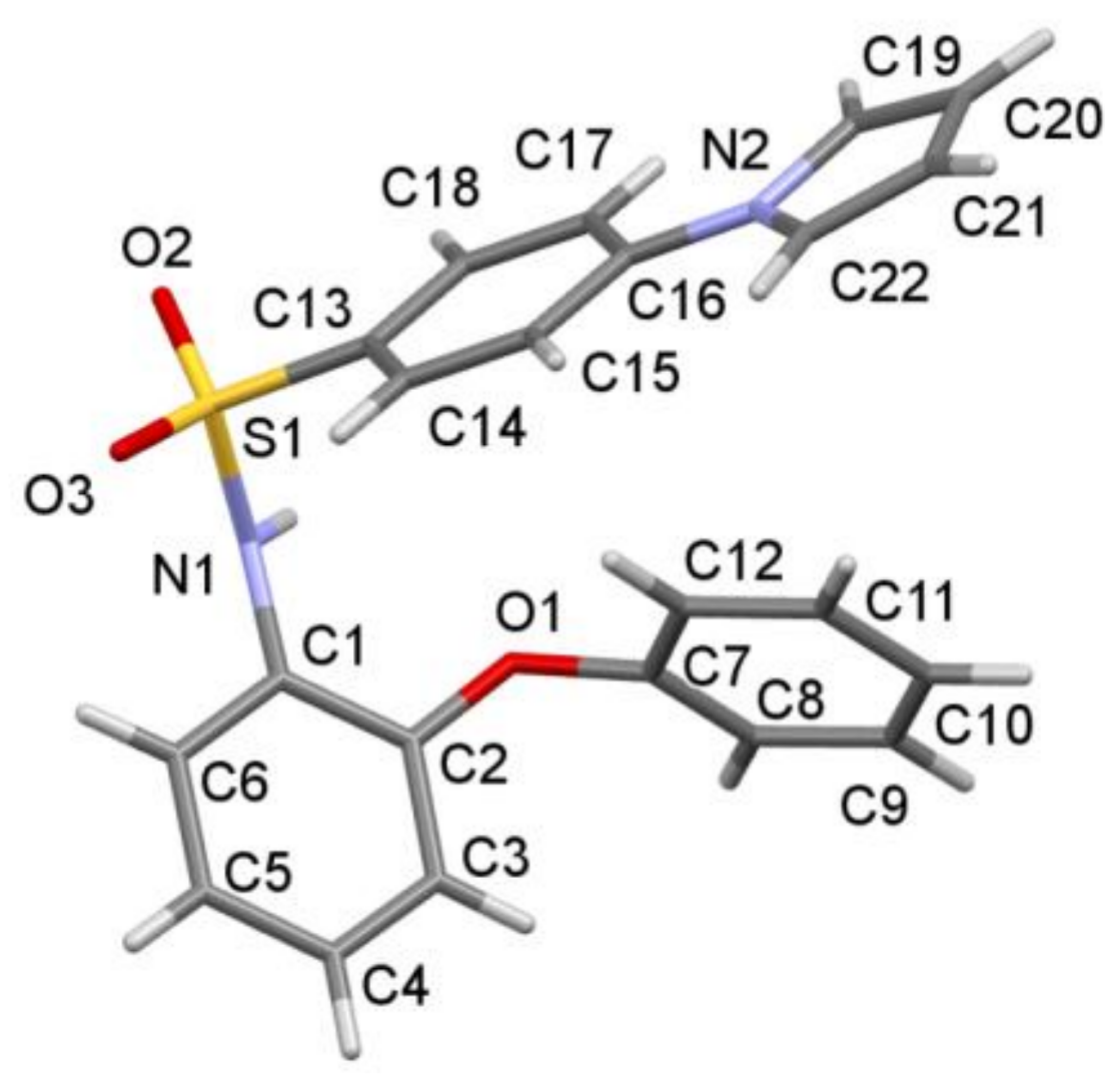
Atom numbering scheme for **25**, hydrogen atoms not labelled for clarity. Selected bond lengths [Å] and angles [°]: S1–O2 1.4287(14), S1–O3 1.4336(14), S1–N1 1.6300(17), S1–C13 1.7646(19), N1–C1 1.427(3), C2–O1 1.392(2), C7–O1 1.406(2); O2–S1–O3 120.24(9), O2–S1–N1 105.80(9), O3–S1–N1 107.69(8), S1–N1–C1 123.68(13), C13–S1–O2 107.80(8), C13–S1–O3 107.79(8), C13–S1–N1 106.83(8), C2–O1–C7 115.53(14).

Details on data collection, structure solution, and refinement are given in Table 1. Compound **25** crystallized in the monoclinic system, and the space group *P*2_1_/*n*, with one molecule in the asymmetric unit, and four in the unit cell, *Z* = 4. The atom numbering scheme is presented in Figure 3. The sulfur atom coordination is close to tetrahedral. The amide group arrangement S(1)–N(1)–C(1) and phenyl ring C1–C6 are not in the same plane, suggesting a lack of coupling between the nitrogen lone pair and aromatic electrons. The phenyl ring C1–C6 and its etheric counterpart C7–C12 are inclined at a dihedral angle of 86.96° (almost perpendicular), while the angle at O1 is rather typical, at 116°. The two other rings, C13–C18 and N2–C19–C22, lay in one plane (the mean planes are inclined at 3.54°). The amide hydrogen atom, found in the Fourier map, forms a slightly pyramidal (not plane) conformation at the nitrogen atom with the sum of angles about N1 equal to 353.2 (<360°).

**Figure 4:**
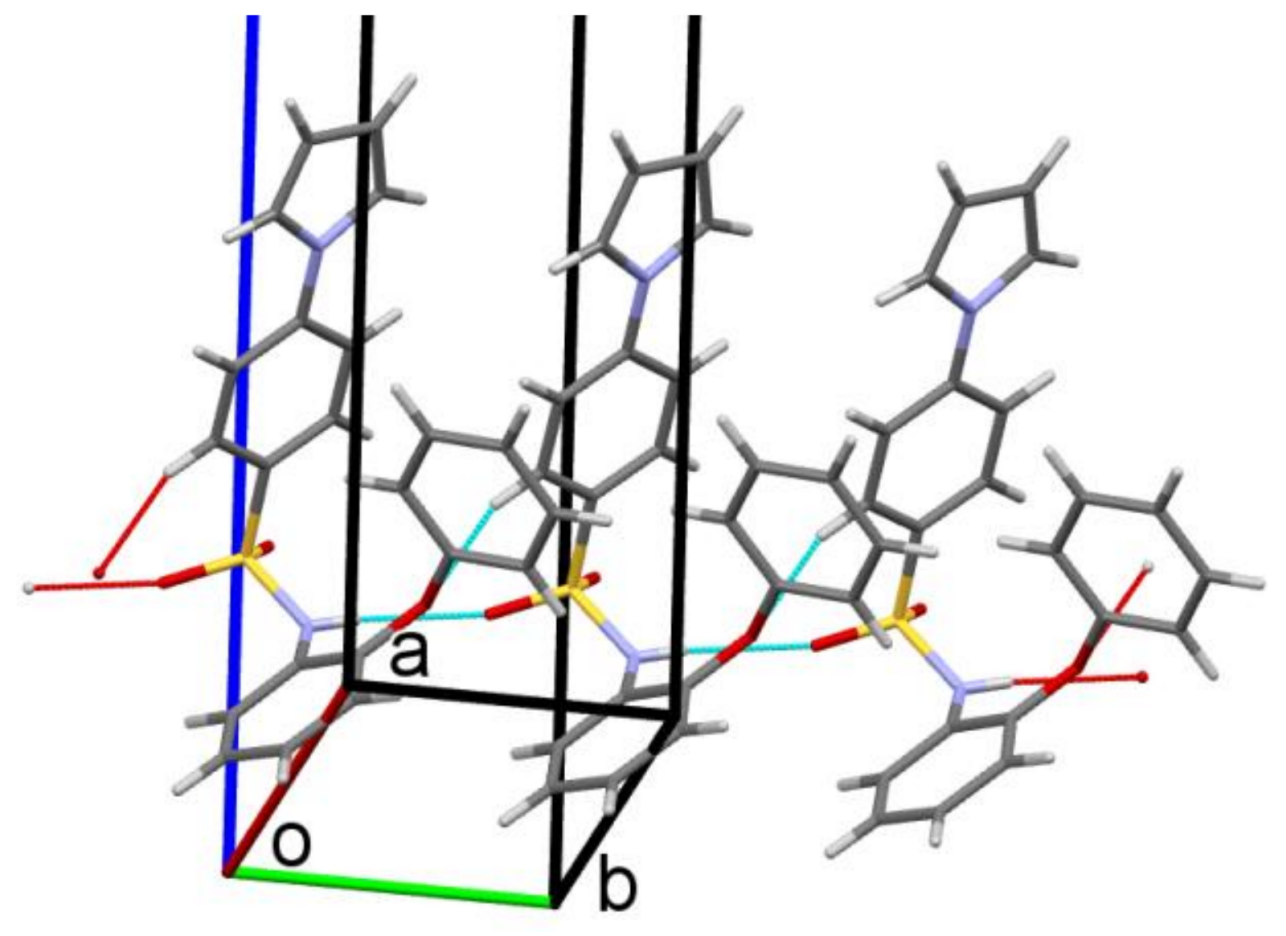
Chain of molecules linked by hydrogen bonding NH···O and CH···O, spreading along the **b** axis with no internal symmetry.

**Figure 5:**
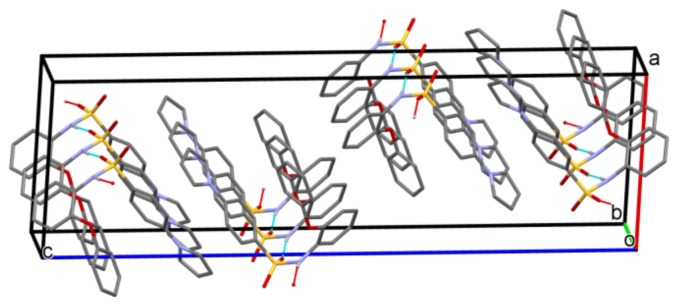
Crystal packing diagram for **25**, hydrogen atoms omitted.

Because of the substitution of the sulfonamide group, only one N–H hydrogen-bond donor is available. The intermolecular hydrogen bonding, of the N–H···O type, forms infinite chains, spreading parallel to the **b** axis (Figure 4, Table 2). The oxygen atom O(3) involved in the interaction is a little more distant from S(1) than the other oxygen atom O(2) (1.4336(14) vs. 1.4287(14) Å), which is expected and confirms hydrogen bonding. The chain has no additional symmetry so it is described by the simplest *p*1 (R1) rod group symmetry [22], and its topology is characterized as a chain with four atoms in the links, graph set symbol C(4) [23]. A weak hydrogen bond of the CH...O type could be also found, which operates within the main chains and may induce the actual conformation of aromatic rings (Table 2). The ring-ring stacking interaction does not play a significant role, as the shortest distance between the ring’s centroids is large (4.47 Å between C7–C12 and C13–C18). The crystal packing diagram suggests the chains are simply packed to maximize the solid density and van der Waals interactions (Figure 5).

### 2.2. Screening for Anticancer Agents

Compounds **23**–**40** were evaluated in vitro for their effects on the viability of three human cancer cell lines: HCT-116 (colon cancer), HeLa (cervical cancer), and MCF-7 (breast cancer). Analysis was performed using the MTT assay after 72 h of incubation, and the calculated IC_50_ values (a concentration required for 50% inhibition of cell viability) are given in Table 3. 

The results of the MTT tests indicated that 4-(1*H*-pyrrol-1-yl)benzenesulfonamide derivatives containing a quinolin-8-yl substituent (**28**–**30**) were the most potent anticancer agents. Among these compounds, **28** exhibited significant cytotoxic activity with 3, 7, and 5 µM IC_50_ values for HCT-116, HeLa, and MCF-7, respectively. It is notable that the presence of a 7-methylquinolin-8-yl substituent in the structure (**30**) caused moderate cytotoxic action against all three lines in the range of IC_50_ = 19–50 µM. On the other hand, the isomeric substituent 2-methylquinolin-8-yl in compound **29** substantially decreased activity in the MCF-7 cells, but retained high activity for the HCT-116 cells (IC_50_ = 9 µM), and reasonable action for the HeLa cells (IC_50_ = 25 µM). In contrast, the *N*-substitution of 4-(1*H-*pyrrol-1-yl)benzenesulfonamide with a 2-methylquinolin-6-yl group (**31**) did not affect the viability of the tested cells (IC_50_ > 100 µM). It is worth noting that incorporation of another bulky aromatic substituent such as benzo-2,1,3-thiadiazol-4-yl moiety (**33**) affected the moderate anticancer properties in the HCT-116 and MCF-7 cell lines, with IC_50_ = 23 µM.

Insertion of a substituted benzene ring (**23**–**25**), as well as nitrogen-containing heterocycles such as pyridine (**26**–**27**, **35**), pyrimidine (**36**, **38**–**39**), and pyridazine (**40**) at location Ar/Het caused decreased activity. The exception was compound **37**, for which the IC_50_ value was obtained in the HCT-116 cells at the concentration of 46 µM. A lack of anticancer activity was also noted for compounds with five-membered rings like 1,2,4-triazole (**32**) and isoxazole (**34**).

In spite of our observed structure activity relationships, for a more objective correlation between structure and activity the QSAR methodology was applied.

### 2.3. QSAR Study

We decided to apply an OPLS-based approach that was successful in our previous studies [17,19,20,21]. Three dimensional structures of all compounds were prepared by Gaussian software by density functional theory (DFT), which is a computational quantum mechanical modeling method. Next, in order to obtain easily interpretable QSAR models, we carefully selected molecular descriptors with clear definition directly reflecting the chemical structures. 

**Figure 6:**
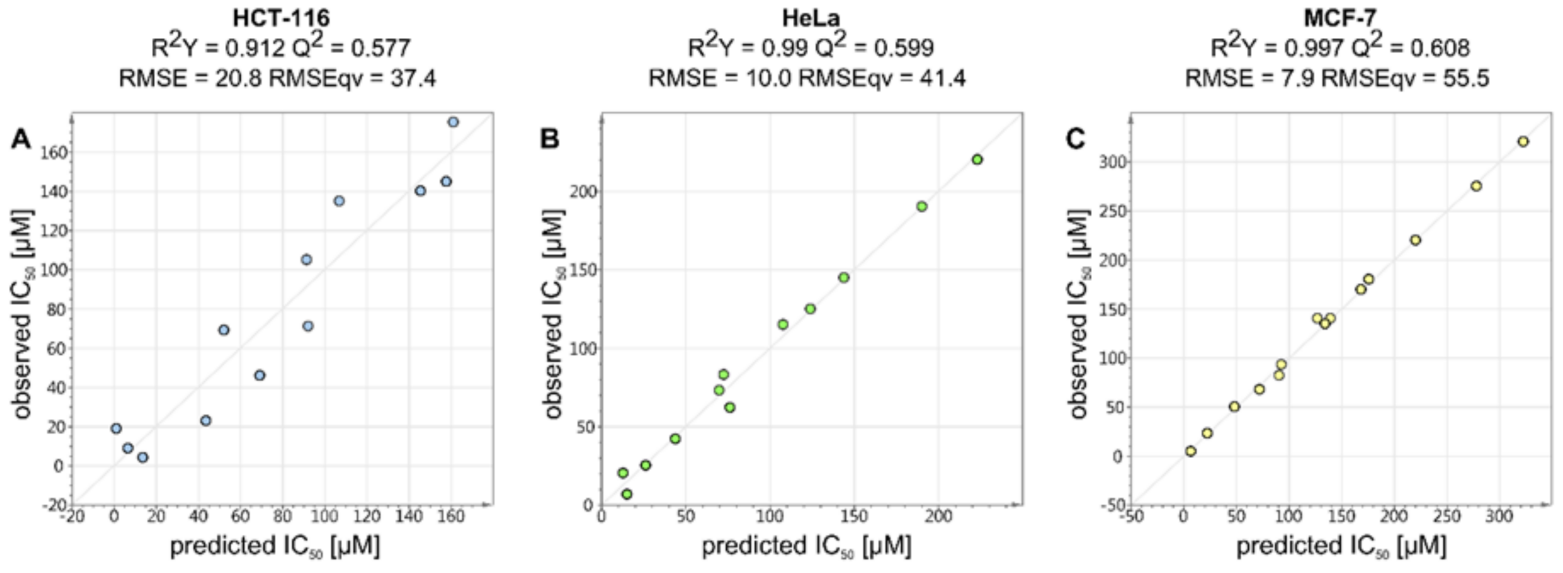
Observed versus predicted values for OPLS-based QSAR models for activity against (**A**) HCT-116; (**B**) HeLa; and (**C**) MCF-7 cell line. R^2^Y—coefficient of determination, Q^2^—cross-validated coefficient of determination, RMSE—root mean squared error, RMSEqv—root mean squared error for cross-validation procedure.

Before application of the regression technique, outlying IC_50_ values were discarded from further analysis. The OPLS calculations, as well as leave-one-out cross validation of the obtained model, were performed by SIMCA. We were able to achieve significant models for all tested cell lines (Figure 6).

The interpretation of the obtained model was based on the calculated VIP (variable importance for projection) values, which sort molecular descriptors by their relative importance for prediction of the IC_50_. The table of VIP values for all descriptors is included in the supplementary material (Table S1). The supplementary material also contains the values of the selected descriptors (Tables S2–S4). 

In the HCT-116 model, the F08[C-O] descriptor was the most influential. This descriptor counts the occurrence of C-O atom pairs in the topological distance 8. The preferred value of this descriptor is low (4 in this set of data). A similar relationship was observed for the B09[C-O] and F09[C-O] descriptors, with a preferred value equal to 0. Additionally, high values for the RBN (rotatable bond number) and RBF (rotatable bond fraction) are related to decreased activity. The same observation was found for the NRS descriptor that counts the number of ring systems. 

The activity of novel sulfonamide derivatives against the HeLa cell line was related to the presence of a hydrogen bond donor and lipophilic center in a topological distance 7 (CATS2D_07_DL descriptor). Such a pair of pharmacophore elements is unprofitable for cytotoxic activity. Similarly, the presence of C and O atoms at a topological distance 9 caused a decrease of activity (B09[C-O] and F09[C-O] descriptors). Analogous to the HCT-116 cell line, RBN and NRS values show that a more rigid structure has higher cytotoxic potency. 

The model derived for the MCF-7 cell line shows the disadvantageous effect of functional groups containing an oxygen atom. This finding is supported by high VIP values for many descriptors, including nO (number of oxygen atoms) and O-060 (number of O-alkyl, and the O-aryl or O-vinyl moieties), among others. The QSAR analysis shows that the presence of O, as well as its pairing with C, N, S and other O atoms at certain topological distances, is unfavorable for cytotoxic activity. 

### 2.4. Apoptosis

#### 2.4.1. Cytotoxic Activity

**Figure 7:**
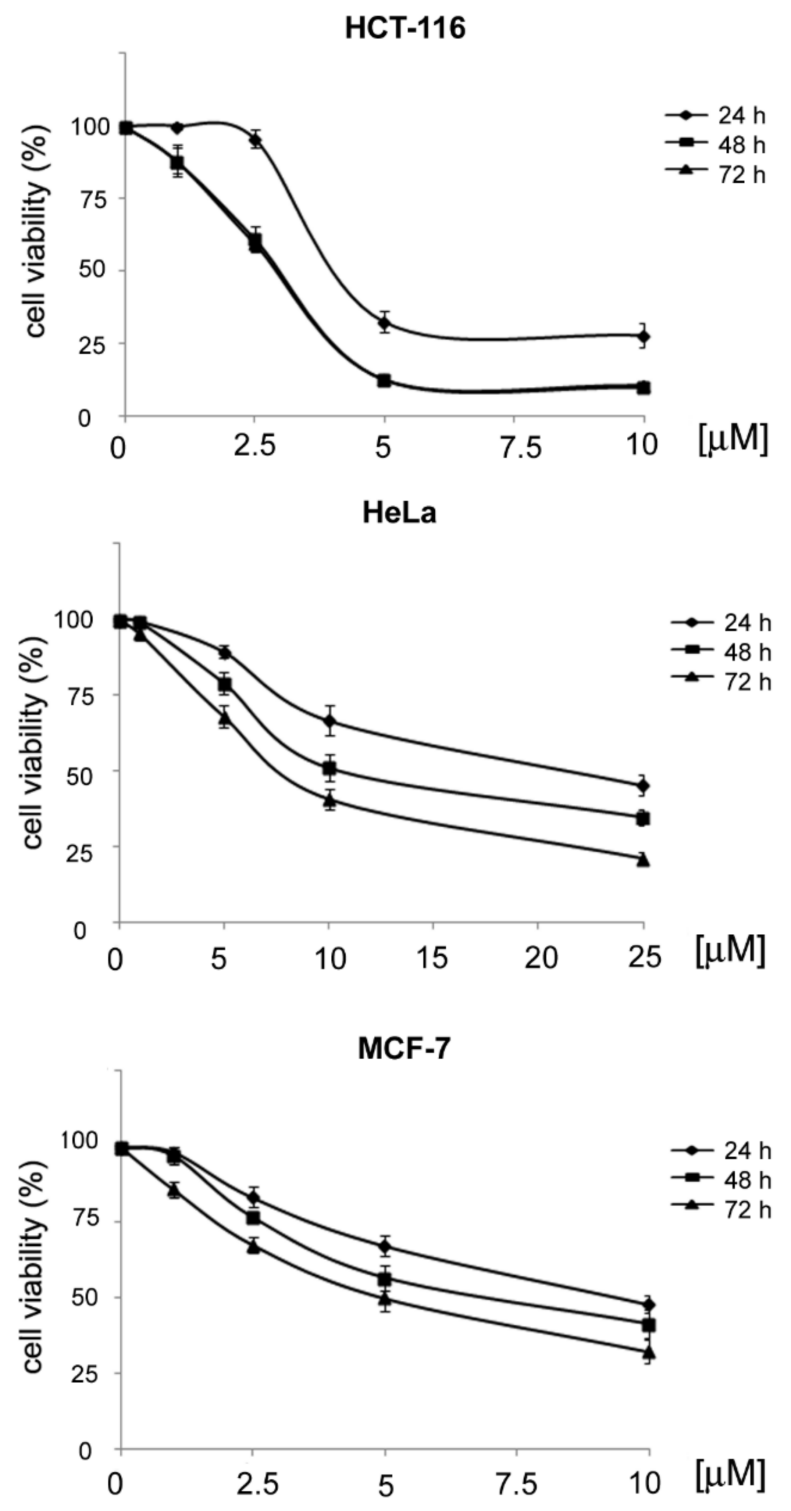
Effects of **28** on the viability of HCT-116, HeLa, and MCF-7 cells. The cells were treated with **28** in the concentration range of 1–25 µM. After 24, 48, and 72 h of incubation, cell viability was assessed with the MTT assay. Values represent mean ± SD of three independent experiments.

On the basis of the results of the cytotoxic screening, compound **28** was chosen to investigate apoptosis-inducing activity in cancer cells. First, the cytotoxic activity of **28** was determined in a time-dependent manner towards HCT-116, HeLa, and MCF-7 cells with the MTT assay (Figure 7). Cells were treated with **28** in the concentration range of 1‒25 µM. For the HCT-116 cells after a 24 h incubation, the IC_50_ value was reached at 4 µM, and a further 24 h incubation decreased this value to 3 µM. It did not decrease further with further treatment. In the case of the HeLa cells, the IC_50_ values were 22, 10, and 7 µM after 24, 48, and 72 h, respectively. The cytotoxic activity of **28** also increased in a concentration-dependent manner towards the MCF-7 cells. After 24 h of incubation, the IC_50_ value was 9.5 µM, further decreased to 7 µM after 48 h, and after 72 h of incubation the IC_50_ value was 5 µM.

#### 2.4.2. Apoptosis Induction

**Figure 8:**
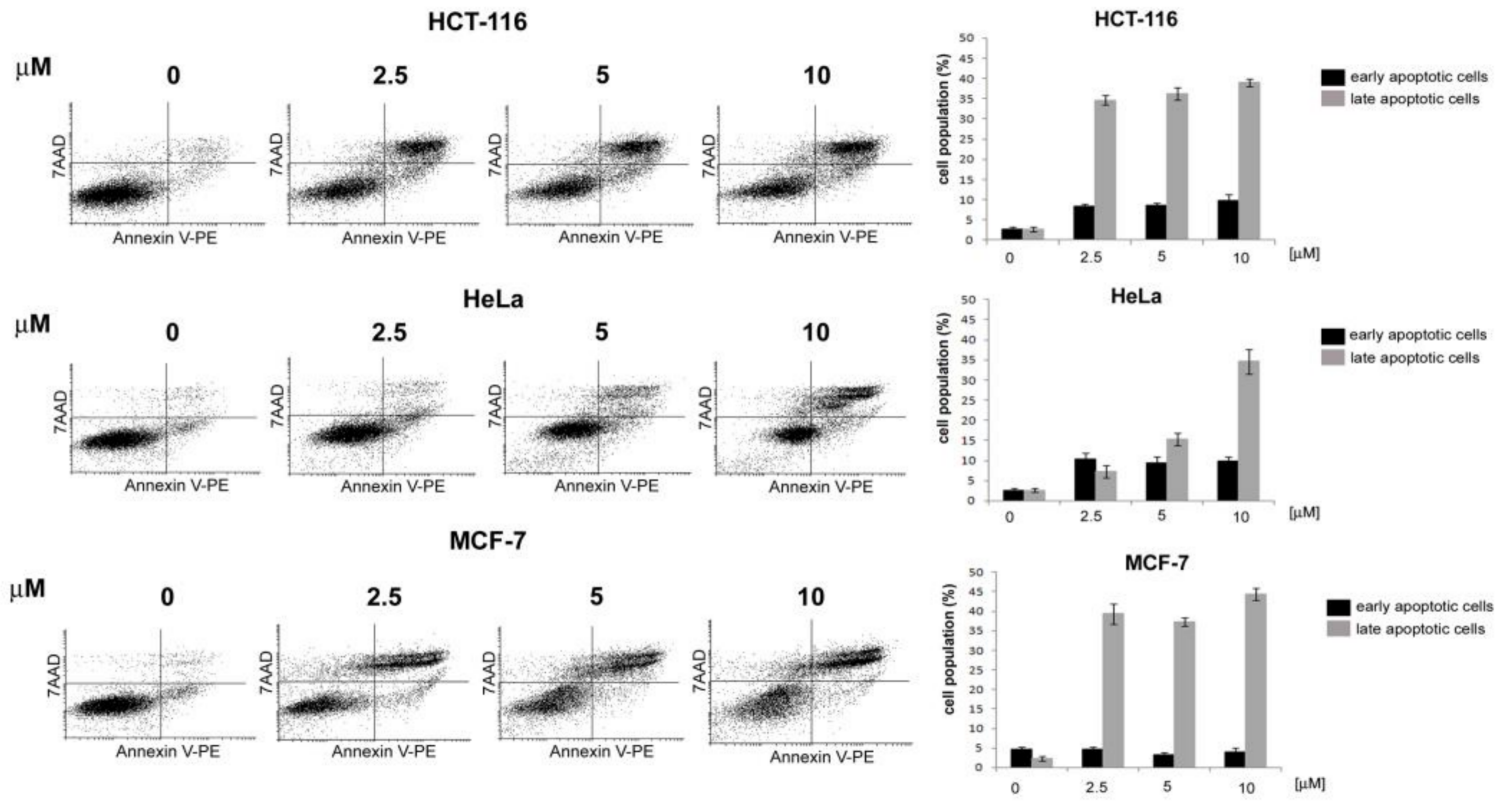
Induction of apoptosis by **28** in HCT-116, HeLa, and MCF-7 cells. Cells were treated with the indicated concentrations of **28** for 48 h (HCT-116) and 72 h (HeLa and MCF-7). Cells were stained with Annexin V-PE and 7-AAD and analyzed with flow cytometry. Dotplots show early apoptotic (bottom right quadrant), late apoptotic (upper right quadrant), viable (lower left quadrant), and necrotic cell populations (upper left quadrant). Values represent mean ± SD of three independent experiments.

In apoptotic cells, the membrane phospholipid, phosphatidylserine, is exposed on the outer leaflet of the plasma membrane and can be detected by fluorochrome-conjugated phosphatidylserine-binding proteins such as Annexin V. In order to quantitatively determine the percentage of cells within a population that are actively undergoing apoptosis, phosphatidylserine externalization induced by **28** was examined by flow cytometric analysis. Cells were treated with 2.5, 5, and 10 µM of **28** for 48 h (HCT-116) and 72 h (HeLa and MCF-7), and stained with Annexin V-PE and 7-AAD. The results shown in Figure 8 indicate that compound **28** induced an increase in the population of early apoptotic cells (~10%) and also significantly increased the percentage of late apoptotic cells (35–40%) in HTC-116 at a concentration range of 2.5–10 µM. Furthermore, treatment of MCF-7 cells with **28** resulted in an increase of cells in the late stage of apoptosis (~40%). In the case of the HeLa cell line, early apoptotic cells (10%) were visible from a concentration of 2.5 µM, while late apoptotic cells increased visibly in a concentration-dependent manner from 7–35%. 

#### 2.4.3. Caspase Activation

**Figure 9:**
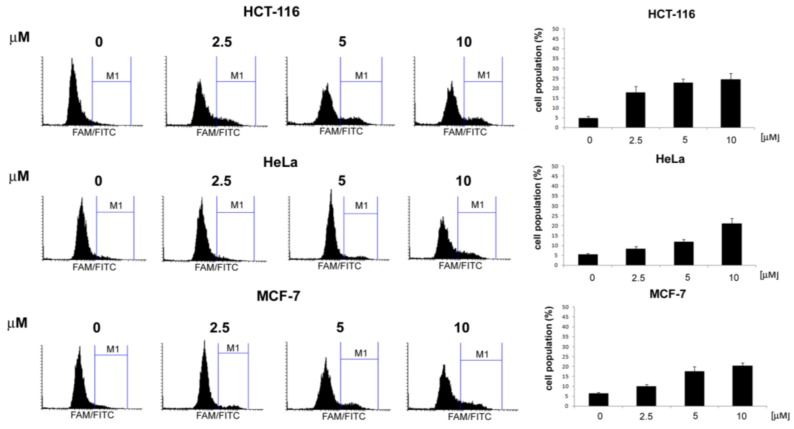
Induction of caspase activity by **28** in HCT-116, HeLa, and MCF-7 cells. Cells were treated with the indicated concentrations of **28** for 24 h (HCT-116) and 48 h (HeLa and MCF-7). Enzyme activity was determined using flow cytometry with the use of a caspase inhibitor, FAM-VAD-FMK. Values represent mean ± SD of three independent experiments.

Apoptotic cells were also identified by determination of levels of active caspases in the cells. Increased caspase activity is caused in response to pro-apoptotic signals and ensures the execution of apoptosis in the cell. Caspase activity induction was determined with the use of a fluorescently labelled caspase inhibitor—FAM-VAD-FMK (a carboxyfluorescein derivative of valylalanylaspartic acid fluoromethyl ketone)—that, through covalently binding with an active enzyme, allowed us to detect the fluorescent enzyme-inhibitor complex using flow cytometry. Increased caspase activation is shown by the increased fluorescence of the caspase inhibitor in the cell population, as indicated by the marker M1 in Figure 9. The results shown in Figure 9 indicate that **28** induced caspase activity in all tested cells in a dose-dependent manner, confirming apoptosis induction by this compound in HCT-116, HeLa, and MCF-7 cells.

#### 2.4.4. Cell Cycle Distribution

**Figure 10:**
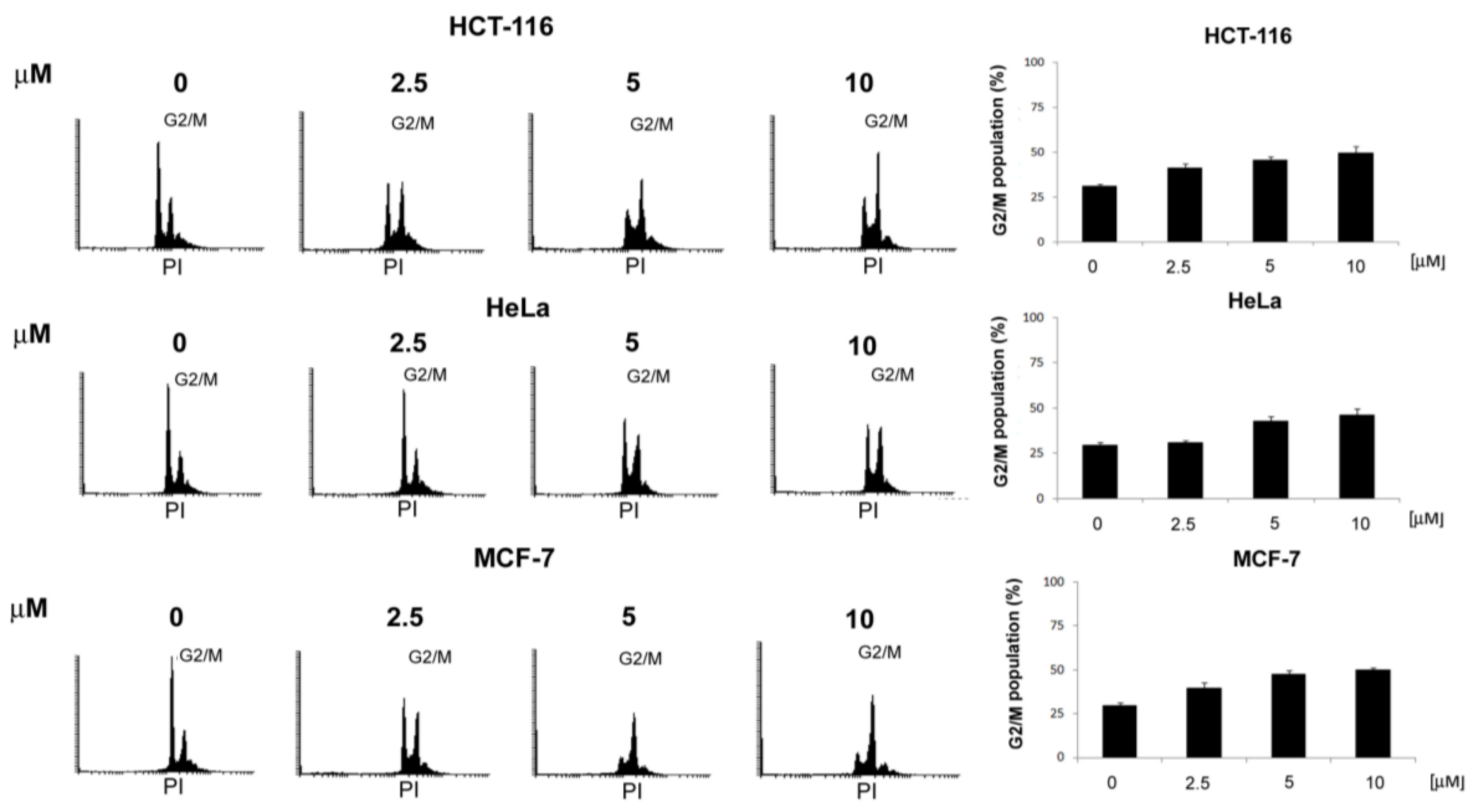
Effects of **28** on G2/M arrest in HCT-116, HeLa, and MCF-7 cells. Cells were treated with the indicated concentrations of **28** for 48 h (HCT-116) and 72 h (HeLa and MCF-7), and cell cycle distribution was analyzed using flow cytometry. Values represent mean ± SD of three independent experiments.

The influence of **28** on changes in cell cycle phase distribution was analyzed with flow cytometry. HCT-116 cells were treated for 48 h with 2.5, 5, and 10 μM (**28**), whereas the HeLa and MCF-7 cells were treated with corresponding concentrations of **28** for 72 h. The results presented in the histograms in Figure 10 show the effects of **28** on cell population distribution in different phases of the cell cycle. Treatment of cells with **28** induced a concentration-dependent increase of the percentage of cells in the G2/M phase of the cell cycle. The increase in G2/M was accompanied by a decrease in the population of cells in the G0/G1 phase of the cell cycle. These results indicate that **28** promotes cell cycle arrest at G2/M in cancer cells.

## 3. Materials and Methods

### 3.1. General Information

Melting points were measured using a Thermogalen (Leica, Vienna, Austia) apparatus and were uncorrected. IR spectra were obtained using a Nicolet iS5 FTIR spectrometer (Thermo Fisher Scientific, Waltham, MA, USA) and spectra were measured in KBr pellets; the absorption range was 400–4000 cm^−1^. ^1^H NMR and ^13^C NMR spectra were obtained using a Varian Gemini 200 apparatus (Varian, Palo Alto, CA, USA) or Varian Unity Plus 500 apparatus. Chemical shifts are expressed as δ values, and resonance multiplicity is described as s (singlet), br. s (broad singlet), d (doublet), dd (doublet of doublets), t (triplet), and m (multiplet). Elemental analyses were obtained using a PerkinElmer 2400 Series II CHN Elemental Analyzer (PerkinElmer, Shelton, CT, USA), and the results indicated by the symbols of the elements that were within ±0.4% of the theoretical values. Thin-layer chromatography (TLC), used for monitoring reactions and for the qualitative analysis of reaction products, was performed on Merck Kieselgel 60 F254 plates (Merck, Darmstadt, Germany) and visualized with UV. Gravity liquid chromatography was conducted using silica gel with a pore size of 60 Å, 220–440 mesh particle size, and 35–75 μm particle size.

The starting compounds of 2-methylquinolin-8-amine [24], and 7-methylquinolin-8-amine [25] were prepared using the reported methods. 4-Amino-*N*-(5-methylisoxazol-3-yl)benzenesulfonamide (**I**), 4-amino-*N*-(pyridin-2-yl)benzenesulfonamide (**II**), 4-amino-*N*-(pyrimidin-2-yl)benzenesulfonamide (**III**), 4-amino-*N*-(4-methylpyrimidin-2-yl)benzenesulfonamide (**IV**), 4-amino-*N*-(4,6-dimethylpyrimidin-2-yl)benzenesulfonamide (**V**), 4-amino-*N*-(2,6-dimethoxypyrimidin-4-yl)benzenesulfonamide (**VI**), and 4-amino-*N*-(6-methoxypyridazin-3-yl)benzenesulfonamide (**VII**) were commercially available. For compounds **36**, **37**, and **38**, only CAS number is available (**36**: 431930-40-4; **37**:371125-83-6; **38**: 431933-86-7). The NMR spectra of the newly synthesized compounds **3**–**5**, **8**, **16**, **19**, **23**, **25**, **27**, and **30** are given in the supplementary material.

### 3.2. Synthesis

#### 3.2.1. General Procedures for the Preparation of 4-Acetamido-*N*-(aryl/heteroaryl)Benzenesulfonamides

*Method A.* To a solution of aminocomponent (3 mmol) in dry pyridine (3 mL), 4-acetamidobenzene-1-sulfonyl chloride (3 mol, 701 mg) was added in portions over 5 min, and reaction mixture was stirred for 2–20 h at room temperature or 105 °C. The reaction mixture was poured into ice-cooled water and stirred for 30 min. The mixture was acidified with dilute hydrochloric acid (1 M) to pH ~ 3–3.5 and stirred for 1 h at room temperature (compd **1**), or with concentrated hydrochloric acid (11.8 M) to pH ~ 5.6–6 and stirred for overnight at room temperature (compds **9**, **10**, **11**), respectively. The precipitated solid was filtered, washed with water (3 × 1 mL), methanol (2 × 1 mL), and dried.

*Method B.* To a solution of aminocomponent (3 mmol) in acetone (3 mL) and pyridine (0.9 mL), 4-acetamidobenzene-1-sulfonyl chloride (3 mmol, 701 mg) was added, and reaction mixture was stirred for 18‒48 h at room temperature. Afterwards, reaction solvents were evaporated under reduced pressure, and the residue was suspended in ice slush (40 mL). Obtained mixture was acidified with dilute hydrochloric acid (1 M) to pH ~ 2 and stirred for 1 h at room temperature. The precipitated solid was filtered, washed with water, and dried. Pure compounds were obtained after crystallization from ethanol.

*4-Acetamido-N-(4-chlorophenyl)benzenesulfonamide* (**1**). *Method A.* Starting from 4-chloroaniline (383 mg), and after 2 h at 105 °C the title compound **1** was obtained (711 mg, 73%): m.p. 195‒196.5 °C (ref. 196‒197.5 °C [26]); IR (KBr) v_max_ 3360, 3226 (NH), 2921 (CH_3_), 1689 (C=O), 1592, 1530 (NH _def_), 1490 (C=C), 1329 (SO_2 asym_), 1150 (SO_2 sym_) cm^−1^; anal. C 51.77, H 4.03, N 8.63% calcd. for C_14_H_13_ClN_2_O_3_S, C 51.70, H 4.00, N 8.62%.

*4-Acetamido-N-[2-(phenylthio)phenyl]benzenesulfonamide* (**2**). *Method B.* Starting from 2-(phenylthio)aniline (604 mg), and after 48 h the title compound **2** was obtained (997 mg, 83%): m.p. 141–144 °C; IR (KBr) v_max_ 3316, 3278, 3247, 3179 (NH), 2917, 2851 (CH_3_), 1662 (C=O), 1594, 1523 (NH _def_), 1474 (C=C), 1336 (SO_2 asym_), 1164 (SO_2 sym_) cm^−1^; ^1^H NMR (500 MHz, DMSO-*d*_6_) δ 2.06 (s, 3H, CH_3_), 7.02 (d, *J* = 8.3 Hz, 1H, arom.), 7.07‒7.13 (m, 4H, arom.), 7.20 (t, 1H, arom.), 7.28‒7.29 (m, 3H, arom.), 7.61 (d, *J* = 8.8 Hz, 2H, arom.), 7.66 (d, *J* = 8.8 Hz, 2H, arom.), 9.61 (s, 1H, NH), 10.29 (s, 1H, NH) ppm; anal. C 60.28, H 4.55, N 7.03% calcd. for C_20_H_18_N_2_O_3_S_2_, C 60.38, H 4.57, N 7.06%.

*4-Acetamido-N-(2-phenoxyphenyl)benzenesulfonamide* (**3**). *Method B.* Starting from 2-(phenoxy)aniline (556 mg), and after 48 h the title compound **3** was obtained (966 mg, 84%): m.p. 161.5‒162.5 °C (ref. 162 °C [27]); IR (KBr) v_max_ 3332, 3289 (NH), 2924 (CH_3_), 1676 (C=O), 1592, 1510 (NH _def_), 1491 (C=C), 1335 (SO_2 asym_), 1160 (SO_2 sym_) cm^−1^; ^1^H NMR (500 MHz, DMSO-*d*_6_) δ 2.07 (s, 3H, CH_3_), 6.65 (d, *J* = 7.9 Hz, 2H, arom.), 6.69 (d, *J* = 7.8 Hz, 1H, arom.), 7.02‒7.10 (m, 3H, arom.), 7.27 (t, 2H, arom.), 7.35 (d, *J* = 7.8 Hz, 1H, arom.), 7.62 (s, 4H, arom.), 9.77 (s, 1H, NH), 10.26 (s, 1H, NH) ppm; ^13^C NMR (125 MHz, DMSO-d_6_) δ 24.8, 119.1, 119.2, 124.1, 124.2, 126.1, 127.1, 128.5, 128.6, 130.4, 134.5, 143.7, 150.2, 156.8, 169.6 ppm; anal. C 60.81, H 4.74, N 7.33% calcd. for C_20_H_18_N_2_O_4_S, C 60.52, H 4.74, N 7.30%.

*4-Acetamido-N-[2-(phenylthio)pyridin-3-yl]benzenesulfonamide* (**4**). *Method B.* Starting from 2-(phenylthio)pyridin-3-amine (607 mg), and after 48 h compound **4** was obtained (902 mg, 75%): m.p. 160‒161 °C; IR (KBr) v_max_ 3366, 3262 (NH), 2923, 2889 (CH_3_), 1692 (C=O), 1592, 1530 (NH _def_), 1441 (C=C), 1328 (SO_2 asym_), 1156 (SO_2 sym_) cm^−1^; ^1^H NMR (500 MHz, DMSO-*d*_6_) δ 2.08 (s, 3H, CH_3_), 7.11 (dd, *J* = 4.9, 7.8 Hz, 1H, arom.), 7.23 (d, *J* = 5.4 Hz, 2H, arom.), 7.28‒7.39 (m, 4H, arom.), 7.66 (d, *J* = 8.3 Hz, 2H, arom.), 7.73 (d, *J* = 8.8 Hz, 2H, arom.), 8.14 (s, 1H, arom.), 10.00 (s, 1H, NH), 10.34 (s, 1H, NH) ppm; ^13^C NMR (125 MHz, DMSO-d_6_) δ 24.8, 119.2, 121.8, 128.8, 129.1, 129.7, 130.9, 131.4, 134.4, 135.2, 135.3, 144.0, 148.3, 156.9, 169.7 ppm; anal. C 57.12, H 4.29, N 10.52% calcd. for C_19_H_17_N_3_O_3_S_2_, C 57.00, H 4.21, N 10.49%.

*4-Acetamido-N-(2-phenoxypyridin-3-yl)benzenesulfonamide* (**5**). *Method B.* Starting from 2-phenoxypyridin-3-amine (559 mg), and after 30 h the title compound **5** was obtained (972 mg, 85%): m.p. 202.1–204.4 °C; IR (KBr) v_max_ 3316, 3275 (NH), 2924, 2853 (CH_3_), 1674 (C=O), 1592, 1512 (NH _def_), 1451 (C=C), 1334 (SO_2 asym_), 1161 (SO_2 sym_) cm^−1^; ^1^H NMR (500 MHz, DMSO-*d*_6_) δ 2.08 (s, 3H, CH_3_), 6.73 (d, *J* = 7.8 Hz, 2H, arom.), 7.08 (dd, *J* = 7.8, 4.9 Hz, 1H, arom.), 7.15 (t, 1H, arom.), 7.30 (t, 2H, arom.), 7.66‒7.70 (m, 4H, arom.), 7.73 (dd, *J* = 7.9, 1.5 Hz, 1H, arom.), 7.83 (dd, *J* = 4.9, 1.5 Hz, 1H, arom.), 10.06 (s, 1H, NH), 10.32 (s, 1H, NH) ppm; ^13^C NMR (125 MHz, DMSO-*d*_6_) δ 24.6, 119.0, 119.6, 121.7, 121.9, 124.9, 128.4, 129.7, 134.0, 134.8, 143.7, 143.9, 153.7, 156.6, 169.5 ppm; anal. C 59.52, H 4.47, N 10.96% calcd. for C_19_H_17_N_3_O_4_S, C 59.37, H 4.49, N 10.93%.

*4-Acetamido-N-(quinolin-8-yl)benzenesulfonamide* (**6**). *Method B.* Starting from quinolin-8-amine (433 mg), and after 25 h the title compound **6** was obtained (800 mg, 78%): m.p. 193‒194 °C (ref. 194 °C [28]); IR (KBr) v_max_ 3365, 3243 (NH), 2926, 2851 (CH_3_), 1691 (C=O), 1597, 1533 (NH _def_), 1504, 1470 (C=N, C=C), 1335 (SO_2 asym_), 1170 (SO_2 sym_) cm^−1^; ^1^H NMR (500 MHz, DMSO-*d*_6_) δ 2.00 (s, 3H, CH_3_), 7.50 (t, 1H, arom.), 7.57 (dd, *J* = 8.3, 3.9 Hz, 1H, arom.), 7.61‒7.68 (m, 4H, arom.), 7.83 (d, *J* = 8.8 Hz, 2H, arom.), 8.34 (dd, *J* = 8.3, 1.5 Hz, 1H, arom.), 8.84 (dd, *J* = 3.9, 1.5 Hz, 1H, arom.), 9.79 (s, 1H, NH), 10.24 (s, 1H, NH) ppm; anal. C 59.81, H 4.43, N 12.31% calcd. for C_17_H_15_N_3_O_3_S, C 59.71, H 4.30, N 12.29%.

*4-Acetamido-N-(2-methylquinolin-8-yl)benzenesulfonamide* (**7**). *Method B.* Starting from 2-methylquinolin-8-amine (475 mg), and after 18 h the title compound **7** was obtained (778 mg, 73%): m.p. 210‒213 °C (ref. 200 °C [29]); IR (KBr) v_max_ 3352, 3243 (NH), 2925 (CH_3_), 1694 (C=O), 1591, 1534 (NH _def_), 1508, 1473 (C=N, C=C), 1342 (SO_2 asym_), 1159 (SO_2 sym_) cm^−1^; ^1^H NMR (500 MHz, DMSO-*d*_6_) δ 2.01 (s, 3H, CH_3_), 2.67 (s, 3H, CH_3_), 7.40‒7.46 (m, 2H, arom.), 7.57‒7.64 (m, 4H, arom.), 7.83 (d, *J* = 8.8 Hz, 2H, arom.), 8.20 (d, *J* = 8.3 Hz, 1H, arom.), 9.60 (s, 1H, NH), 10.24 (s, 1H, NH) ppm; anal. C 60.83, H 4.82, N 11.82% calcd. for C_18_H_17_N_3_O_3_S, C 60.49, H 5.16, N 11.53%.

*4-Acetamido-N-(7-methylquinolin-8-yl)benzenesulfonamide* (**8**). *Method B.* Starting from 7-methylquinolin-8-amine (475 mg), and after 22 h the title compound **8** (778 mg, 73%) was obtained: m.p. 232‒235 °C; IR (KBr) v_max_ 3361, 3224 (NH), 2928 (CH_3_), 1692 (C=O), 1598, 1541 (NH _def_), 1502, 1464 (C=N, C=C), 1333 (SO_2 asym_), 1170 (SO_2 sym_) cm^−1^; ^1^H NMR (500 MHz, DMSO-*d*_6_) δ 2.04 (s, 3H, CH_3_), 2.55 (s, 3H, CH_3_), 7.32 (dd, *J* = 8.3, 4.4 Hz, 1H, arom.), 7.44 (d, *J* = 8.8 Hz, 2H, arom.), 7.48‒7.53 (m, 3H, arom.), 7.77 (d, *J* = 8.3 Hz, 1H, arom.), 8.22 (dd, *J* = 8.3, 1.5 Hz, 1H, arom.), 8.41 (dd, *J* = 3.9, 1.4 Hz, 1H, arom.), 9.49 (s, 1H, NH), 10.18 (s, 1H, NH) ppm; ^13^C NMR (125 MHz, DMSO-*d*_6_) δ 19.93, 24.6, 118.1, 121.1, 126.8, 127.1, 128.6, 130.3, 131.6, 134.8, 136.2, 138.5, 143.0, 144.4, 149.6, 169.3 ppm; anal. C 60.83, H 4.82, N 11.82% calcd. for C_18_H_17_N_3_O_3_S, C 60.66, H 4.71, N 11.82%.

*4-Acetamido-N-(2-methylquinolin-6-yl)benzenesulfonamide* (**9**). *Method A.* Starting from 2-methylquinolin-6-amine (475 mg), and after 20 h at room temperature the title compound **9** was obtained (938 mg, 88%): m.p. 265‒267 °C; IR (KBr) v_max_ 3353, 3236 (NH), 2921 (CH_3_), 1673 (C=O), 1611, 1591 (NH _def_), 1545, 1515, 1459 (C=N, C=C), 1320 (SO_2 asym_), 1155 (SO_2 sym_) cm^−1^; ^1^H NMR (500 MHz, DMSO-*d*_6_) δ 2.02 (s, 3H, CH_3_), 2.58 (s, 3H, CH_3_), 7.33 (d, 1H, arom.), 7.43 (dd, 1H, arom.), 7.53 (s, 1H, arom.), 7.66 (d, *J* = 8.8 Hz, 2H, arom.), 7.72 (d, *J* = 8.8 Hz, 2H, arom.), 7.77 (d, 1H, arom.), 8.12 (d, 1H, arom.), 10.26 (s, 1H, NH), 10.47 (s, 1H, NH) ppm; anal. C 60.83, H 4.82, N 11.82% calcd. for C_18_H_17_N_3_O_3_S, C 60.53, H 4.02, N 11.45%.

*4-Acetamido-N-(4H-1,2,4-triazol-4-yl)benzenesulfonamide* (**10**). *Method A.* Starting from 4H-1,2,4-triazol-4-amine (475 mg), and after 3 h at 105 °C the title compound **10** was obtained (472 mg, 56%): m.p. 227–228 °C (205 °C (decomp.) [30] ; IR (KBr) v_max_ 3296, 3251 (NH), 2931, 2861 (CH_3_), 1673 (C=O), 1604, 1590 (NH _def_), 1536, 1497 (C=N, C=C), 1334 (SO_2 asym_), 1163 (SO_2 sym_) cm^−1^; ^1^H NMR (500 MHz, DMSO-d_6_) δ 2.09 (s, 3H, CH_3_), 7.61 (d, *J* = 8.8 Hz, 2H, arom.), 7.81 (d, *J* = 8.8 Hz, 2H, arom.), 8.27 (s, 2H, arom.), 10.45 (s, 1H, NH), 11.80 (s, 1H, NH) ppm; ^13^C NMR (125 MHz, DMSO-*d*_6_) δ 24.9, 119.6, 129.1, 129.9, 143.5, 145.4, 170.0 ppm; anal. C 42.70, H 3.94, N 24.90% calcd. for C_10_H_11_N_5_O_3_S, C 42.38, H 3.71, N 24.50%.

*4-Acetamido-N-(benzo[c][1,2,5]thiadiazol-4-yl)benzenesulfonamide* (**11**). *Method A.* Starting from benzo[c][1,2,5]thiadiazol-4-amine (454 mg), and after 18 h at room temperature the title compound **11** was obtained (562 mg, 54%): m.p. 206‒207 °C; IR (KBr) v_max_ 3376, 3222 (NH), 2921 (CH_3_), 1676 (C=O), 1612, 1587 (NH _def_), 1543, 1493 (C=N, C=C), 1347 (SO_2 asym_), 1158 (SO_2 sym_) cm^−1^; ^1^H NMR (500 MHz, DMSO-*d*_6_) δ 2.04 (s, 3H, CH_3_), 7.40 (d, 1H, arom.), 7.61 (t, 1H, arom.), 7.67 (d, *J* = 8.8 Hz, 2H, arom.), 7.75 (d, 1H, arom.), 7.81 (d, *J* = 8.8 Hz, 2H, arom.), 10.29 (s, 1H, NH), 10.87 (s, 1H, NH) ppm; anal. C 48.26, H 3.47, N 16.08% calcd. for C_14_H_12_N_4_O_3_S_2_, C 48.58, H 3.21, N 16.30%.

#### 3.2.2. General Procedures for the Synthesis of 4-Amino-*N*-(aryl/heteroaryl)Benzenesulfonamides (**12**–**22**)

*Method A:* The 4-acetamido-*N*-(aryl/heteroaryl)benzenesulfonamide (2.5 mmol) was heated at 100 °C with a solution of NaOH (25 mmol) in water (11.5 mL) for 1 h; then, the solution was cooled and acidified to pH ~ 5 with 50% acetic acid. The solid was filtered off, washed with water (3 × 5 mL), 50% methanol (2 × 2.5 mL), and methanol (2.5 mL), and dried. 

*Method B*: The 4-acetamido-*N*-(aryl/heteroaryl)benzenesulfonamide (2.5 mmol) was heated at 100 °C with hydrochloric acid (4 mL, 36%) in ethanol (10 mL) for 1 h, than the solution was cooled, treated with cold water (20 mL), and basified with ammonia to pH ~ 8–9. The solid was filtered off, washed with water (3 × 10 mL), and purified as indicated below. 

*4-Amino-N-(4-chlorophenyl)benzenesulfonamide* (**12**). Hydrolysis according to *Method A*. Starting from **1** (812 mg), compound **12** (664 mg, 94%) was obtained: m.p. 195.5‒196 °C (ref. 194‒195 °C [26]); IR (KBr) v_max_ 3412, 3346 (NH), 2923, 2855 (CH_3_), 1635, 1597 (NH _def_), 1493 (C=C), 1314 (SO_2 asym_), 1152 (SO_2 sym_) cm^−1^; anal. C 50.97, H 3.92, N 9.91% calcd. for C_12_H_11_ClN_2_O_2_S, C 60.00, H 3.93, N 9.94%.

*4-Amino-N-[2-(phenylthio)phenyl]benzenesulfonamide* (**13**). Hydrolysis according to *Method B*. Starting from **2** (996 mg) and after purification on silica gel using benzene as the eluent, the title compound **13** (838 mg, 94%) was obtained as an oil; IR (NaCl plates) v_max_ 3479, 3377, 3304, 3228 (NH), 1626, 1594 (NH _def_), 1478 (C=C), 1316 (SO_2 asym_), 1151 (SO_2 sym_) cm^−1^; ^1^H NMR (500 MHz, DMSO-*d*_6_) δ 6.01 (s, 2H, NH_2_), 6.51 (d, *J* = 7.8 Hz, 2H, arom.), 7.06‒7.12 (m, 4H, arom.), 7.16‒7.24 (m, 2H, arom.), 7.28‒7.34 (m, 5H, arom.), 9.03 (s, 1H, NH) ppm; ^13^C NMR (125 MHz, DMSO-*d*_6_) δ 113.2, 124.8, 125.1, 126.9, 128.0, 129.1, 129.6, 130.2, 130.5, 131.3, 133.7, 135.6, 137.7, 153.7 ppm.

*4-Amino-N-(2-phenoxyphenyl)benzenesulfonamide* (**14**). Hydrolysis according to *Method B*. Starting from **3** (956 mg) and after crystallization from ethanol, the title compound **14** (817 mg, 96%) was obtained: m.p. 142 °C (ref. 149 °C [27]; IR (KBr) v_max_ 3498, 3394, 3305 (NH), 1630, 1596 (NH _def_), 1490 (C=C), 1315 (SO_2 asym_), 1147 (SO_2 sym_) cm^−1^; ^1^H NMR (500 MHz, DMSO-*d*_6_) δ 6.00 (br. s, 2H, NH_2_), 6.49 (d, *J* = 8.3 Hz, 2H, arom.), 6.68‒6.74 (m, 3H, arom.), 7.00‒7.04 (m, 2H, arom.), 7.10 (t, 1H, arom.), 7.28‒7.38 (m, 5H, arom.), 9.36 (s, 1H, NH) ppm; anal. C 63.51, H 4.74, N 8.23% calcd. for C_18_H_16_N_2_O_3_S, C 63.57, H 4.60, N 8.21%.

*4-Amino-N-[2-(phenylthio)pyridin-3-y]benzenesulfonamide* (**15**). Hydrolysis according to *Method B*. Starting from **4** (999 mg) and after crystallization from ethanol, the title compound **15** (742 mg, 83%) was obtained: m.p. 146.8–147.6 °C dec.; IR (KBr) v_max_ 3451, 3362, 3268 (NH), 1635, 1593 (NH _def_), 1500, 1444 (C=N, C=C), 1312 (SO_2 asym_), 1156 (SO_2 sym_) cm^−1^; ^1^H NMR (500 MHz, DMSO-*d*_6_) δ 6.00 (br. s, 2H, NH_2_), 6.57 (d, *J* = 8.3 Hz, 2H, arom.), 7.10 (dd, *J* = 8.1, 4.7 Hz, 1H, arom.), 7.27‒7.31 (m, 3H, arom.), 7.35‒7.37 (m, 5H, arom.), 8.12 (dd, *J* = 4.4, 1.5 Hz, 1H, arom.), 9.53 (s, 1H, NH) ppm; anal. C 57.12, H 4.23, N 11.76% calcd. for C_17_H_15_N_3_O_2_S_2_, C 56.89, H 4.07, N 11.60%.

*4-Amino-N-(2-phenoxypyridin-3-yl)benzenesulfonamide* (**16**). Hydrolysis according to *Method B*. Starting from **5** (959 mg) and after crystallization from ethanol, the title compound **16** (725 mg, 85%) was obtained: m.p. 163.1–164.7 °C; IR (KBr) v_max_ 3474, 3350, 3237 (NH), 1635, 1594 (NH _def_), 1492, 1448 (C=N, C=C), 1325 (SO_2 asym_), 1157 (SO_2 sym_) cm^−1^; ^1^H NMR (500 MHz, DMSO-*d*_6_) δ 6.03 (s, 2H, NH_2_), 6.54 (d, *J* = 8.3 Hz, 2H, arom.), 6.82 (d, *J* = 7.9 Hz, 2H, arom.), 7.05 (dd, *J* = 7.9, 4.9 Hz, 1H, arom.), 7.16 (t, 1H, arom.), 7.34 (t, 2H, arom.), 7.38 (d, *J* = 8.8 Hz, 2H, arom.), 7.71 (dd, *J* = 7.8, 1.5 Hz, 1H, arom.), 7.79 (dd, *J* = 4.4, 1.5 Hz, 1H, arom.), 9.66 (s, 1H, NH) ppm; ^13^C NMR (125 MHz, DMSO-*d*_6_) δ 113.0, 119.5, 121.9, 122.7, 124.8, 125.1, 129.2, 129.7, 133.2, 143.0, 153.6, 153.9, 156.1 ppm; anal. C 59.81, H 4.43, N 12.31% calcd. for C_17_H_15_N_3_O_3_S, C 59.58, H 4.41, N 12.28%.

*4-Amino-N-(quinolin-8-yl)benzenesulfonamide* (**17**). Hydrolysis according to *Method B*. Starting from **6** (853 mg) the title compound **17** (696 mg, 93%) was obtained: m.p. 193‒193.3 °C (ref. 193 °C [31]); IR (KBr) v_max_ 3468, 3375, 3252 (NH), 1628, 1593 (NH _def_), 1504, 1472 (C=N, C=C), 1326 (SO_2 asym_), 1153 (SO_2 sym_) cm^−1^; ^1^H NMR (500 MHz, DMSO-*d*_6_) δ 6.00 (s, 2H, NH_2_), 6.46 (d, *J* = 8.8 Hz, 2H, arom.), 7.49‒7.53 (m, 3H, arom.), 7.58‒7.61 (m, 2H, arom.), 7.64 (d, *J* = 7.8 Hz, 1H, arom.), 8.35 (d, *J* = 8.3 Hz, 1H, arom.), 8.86 (d, *J* = 4.4 Hz, 1H, arom.), 9.36 (s, 1H, NH) ppm; anal. C 60.18, H 4.38, N 14.04% calcd. for C_15_H_13_N_3_O_2_S, C 60.12, H 4.41, N 13.98%.

*4-Amino-N-(2-methylquinolin-8-yl)benzenesulfonamide* (**18**). Hydrolysis according to *Method B*. Starting from **7** (888 mg) and after crystallization from ethanol, the title compound **18** (689 mg, 88%) was obtained: m.p. 156‒158 °C (ref. m.p. was not determined [29]); IR (KBr) v_max_ 3482, 3382, 3265 (NH), 1620, 1593 (NH _def_), 1506, 1472 (C=N, C=C), 1345 (SO_2 asym_), 1155 (SO_2 sym_) cm^−1^; ^1^H NMR (500 MHz, DMSO-*d*_6_) δ 2.68 (s, 3H, CH_3_), 6.00 (s, 2H, NH_2_), 6.47 (d, *J* = 8.8 Hz, 2H, arom.), 7.41 (t, 1H, arom.), 7.46 (d, *J* = 8.3 Hz, 1H, arom.), 7.51‒7.57 (m, 4H, arom.), 8.21 (d, *J* = 8.3 Hz, 1H, arom.), 9.23 (s, 1H, NH) ppm; anal. C 61.32, H 4.82, N 13.41% calcd. for C_16_H_15_N_3_O_2_S, C 61.25, H 4.89, N 13.21%.

*4-Amino-N-(7-methylquinolin-8-yl)benzenesulfonamide* (**19**). Hydrolysis according to *Method B*. Starting from **8** (888 mg) and after crystallization from ethanol, the title compound **19** (705 mg, 90%) was obtained: m.p. 202‒203 °C; IR (KBr) v_max_ 3477, 3378, 3239 (NH), 1630, 1595 (NH _def_), 1503, 1465 (C=N, C=C), 1316 (SO_2 asym_), 1153 (SO_2 sym_) cm^−1^; ^1^H NMR (500 MHz, DMSO-*d*_6_) δ 2.55 (s, 3H, CH_3_), 5.79 (s, 2H, NH_2_), 6.31 (d, *J* = 8.8 Hz, 2H, arom.), 7.12 (d, *J* = 8.7 Hz, 2H, arom.), 7.36 (dd, *J* = 8.1, 4.2 Hz, 1H, arom.), 7.48 (d, *J* = 8.3 Hz, 1H, arom.), 7.73 (d, *J* = 8.8 Hz, 1H, arom.), 8.22 (d, *J* = 7.8 Hz, 1H, arom.), 8.55 (d, *J* = 3.9 Hz, 1H, arom.), 9.01 (s, 1H, NH) ppm; ^13^C NMR (125 MHz, DMSO-*d*_6_) δ 20.0, 112.2, 121.1, 125.6, 126.2, 126.9, 129.4, 130.3, 132.1, 136.2, 137.4, 144.1, 149.6, 152.9 ppm; anal. C 61.32, H 4.82, N 13.41% calcd. for C_16_H_15_N_3_O_2_S, C 61.24, H 4.76, N 13.40%. 

*4-Amino-N-(2-methylquinolin-6-yl)benzenesulfonamide* (**20**). Hydrolysis according to *Method A*. Starting from **9** (888 mg) and after crystallization from acetone, the title compound **20** (548 mg, 70%) was obtained: m.p. 249‒252 °C (ref. 252 °C [32]); IR (KBr) v_max_ 3474, 3308, 3235, 3176 (NH), 1638, 1597 (NH _def_), 1507, 1429 (C=N, C=C), 1335 (SO_2 asym_), 1149 (SO_2 sym_) cm^−1^; anal. C 61.32, H 4.82, N 13.41% calcd. for C_16_H_15_N_3_O_2_S, C 61.56, H 4.98, N 13.52%. 

*4-Amino-N-(4H-1,2,4-triazol-4-yl)benzenesulfonamide* (**21**). Hydrolysis according to *Method A*. Starting from **10** (703 mg) the title compound **21** (532 mg, 89%) was obtained: m.p. 231‒232 °C (ref. 237 °C [33]); IR (KBr) v_max_ 3415, 3337, 3213, 3148 (NH), 1658, 1595 (NH _def_), 1529, 1457 (C=N, C=C), 1348 (SO_2 asym_), 1164 (SO_2 sym_) cm^−1^; anal. C 40.16, H 3.79, N 29.27% calcd. for C_8_H_9_N5O_2_S, C 40.48, H 3.96, N 28.77%.

*4-Amino-N-(benzo[c][1,2,5]thiadiazol-4-yl)benzenesulfonamide* (**22**). Hydrolysis according to *Method A*. Starting from **11** (871 mg) the title compound **22** (659 mg, 86%) was obtained: m.p. 206‒207 °C (ref. m.p. was not determined [34]); IR (KBr) v_max_ 3468, 3379, 3329, 3201 (NH), 1626, 1591 (NH _def_), 1541, 1496, 1449 (C=N, C=C), 1342 (SO_2 asym_), 1158 (SO_2 sym_) cm^−1^; anal. C 47.04, H 3.29, N 18.29% calcd. for C_12_H_10_N_4_O_2_S_2_, C 46.77, H 2.96, N 17.91%.

#### 3.2.3. General Procedures for the Synthesis of *N*-(aryl/heteroaryl)-4-(1-*H*-pyrrol-1-yl)Benzenesulfonamides (**23**–**40**)

*Method A.* A mixture of 2,5-dimethoxytetrahydrofuran (198 mg, 1.5 mmol), *p*-dioxane (1.5 mL), glacial acetic acid (0.75 mL), and the corresponding 4-amino-*N*-(aryl/heteroaryl)benzenesulfonamide (**13**–**19**, **I**, **II**) (1.45 mmol) was stirred at reflux for 24 h. After cooling to room temperature, the mixture was evaporated in vacuum to dryness, and a residue was dissolved in boiling ethanol. After standing overnight, the precipitate was collected by filtration, washed with ethanol (2 × 2 mL), and dried at 105 °C.

*Method B.* A mixture of 2,5-dimethoxytetrahydrofuran (198 mg, 1.5 mmol), *p*-dioxane (1 mL), glacial acetic acid (2 mL), and the corresponding 4-amino-*N*-(heteroaryl)benzenesulfonamide (**20**, **21**, **VII**) (1.4 mmol) was stirred at reflux for 24 h. After cooling to room temperature and standing overnight, the precipitate was collected by filtration, washed successively with *p*-dioxane (3 × 1.5 mL), water (4 × 2 mL) and ethanol (3 × 1.5 mL), and dried at temperatures gradually increasing to 105 °C. 

*Method C.* A mixture of 2,5-dimethoxytetrahydrofuran (203 mg, 1.54 mmol), *p*-dioxane (2 mL), glacial acetic acid (1 mL), and the corresponding 4-amino-*N*-(heteroaryl)benzenesulfonamide (**22**, **III**–**VI**) (1.45 mmol) was stirred at reflux for 26 h. After cooling to room temperature and standing overnight, the precipitate was collected by filtration, washed successively with *p*-dioxane (2 × 2 mL), water (4 × 2 mL), and ethanol (3 × 3 mL), and dried at temperatures gradually increasing to 110 °C. 

*N-(4-Chlorophenyl)-4-(1H-pyrrol-1-yl)benzenesulfonamide* (**23**). A mixture of 2,5-dimethoxytetrahydrofuran (198 mg, 1.5 mmol), *p*-dioxane (1 mL), glacial acetic acid (2 mL), and 4-amino-*N*-(4-chlorophenyl)benzenesulfonamide **12** (410 mg, 1.4 mmol) was stirred at reflux for 24 h. After cooling to room temperature and standing overnight, the small amount (8 mg) of insoluble side products was filtered out, and the filtrate was concentrated in vacuum. To the residue water, 5 mL was added and stirred at room temperature for 6 h. The precipitate was collected by filtration, washed successively with water (6 × 1 mL) and ethanol (3 × 1 mL), and dried at temperatures gradually increasing to 105 °C. Yield for **23** (434 mg, 90%): m.p. 141‒142 °C; IR (KBr) 3255 (NH), 1596 (NH _def_), 1507, 1489, 1436 (C=N, C=C), 1333 (SO_2 asym_), 1157 (SO_2 sym_) cm^−1^; ^1^H NMR (500 MHz, DMSO-*d*_6_) δ 6.30 (t, 2H, H-3 and H-4, pyrrole), 7.11 (d, *J* = 8.8 Hz, 2H, H-3 and H-5, 4-ClPh), 7.30 (d, *J* = 8.8 Hz, 2H, H-2 and H-6, 4-ClPh), 7.47 (t, 2H, H-2 and H-5, pyrrole), 7.77 (s, 4H, PhSO_2_), 10.43 (s, 1H, SO_2_NH) ppm; ^13^C NMR (125 MHz, DMSO-*d*_6_) δ 112.4, 119.7, 119.8, 122.4, 129.0, 129.2, 129.9, 135.6, 137.4, 143.6 ppm; anal. C 57.74, H 3.93, N 8.41% calcd. for C_16_H_13_ClN_2_O_2_S, C 57.80, H 4.02, N 8.43%.

*N-(2-(Phenylthio)phenyl)-4-(1H-pyrrol-1-yl)benzenesulfonamide* (**24**). *Method A.* Starting from 4-amino-*N*-[2-(phenylthio)phenyl]benzenesulfonamide **13** (517 mg), the title compound **24** was obtained (413 mg, 70%): m.p. 121.8‒125.0 °C; IR (KBr) 3231 (NH), 1596 (NH _def_), 1509, 1475, 1440 (C=N, C=C), 1337 (SO_2 asym_), 1168 (SO_2 sym_) cm^−1^; ^1^H NMR (500 MHz, DMSO-*d*_6_) δ 6.32 (t, 2H, H-3 and H-4, pyrrole), 7.02‒7.07 (m, 3H, arom.), 7.15‒7.18 (m, 2H, arom.), 7.21‒7.25 (m, 4H, arom.), 7.48 (t, 2H, H-2 and H-5, pyrrole), 7.73 (s, 4H, PhSO_2_), 9.86 (s, 1H, SO_2_NH) ppm; anal. C 65.00, H 4.46, N 6.89% calcd. for C_22_H_18_N_2_O_2_S_2_, C 64.69, H 4.58, N 6.88%. 

*N-(2-Phenoxyphenyl)-4-(1H-pyrrol-1-yl)benzenesulfonamide* (**25**). *Method A.* Starting from 4-amino-*N*-(2-phenoxyphenyl)benzenesulfonamide **14** (554 mg), the title compound **25** was obtained (396 mg, 70%): m.p. 146 °C; IR (KBr) 3309 (NH), 1598 (NH _def_), 1510, 1491, 1421 (C=N, C=C), 1338 (SO_2 asym_), 1168 (SO_2 sym_) cm^−1^; ^1^H NMR (500 MHz, DMSO-*d*_6_) δ 6.31 (t, 2H, H-3 and H-4, pyrrole), 6.63 (d, *J* = 7.8 Hz, 2H, arom.), 6.70 (d, *J* = 7.3 Hz, 1H, arom.), 7.00 (t, 1H, arom.), 7.06‒7.13 (m, 2H, arom.), 7.19 (t, 2H, arom.), 7.40 (d, *J* = 7.3 Hz, 1H, arom.), 7.44 (t, 2H, H-2 and H-5, pyrrole), 7.66 (d, *J* = 8.8 Hz, 2H, PhSO_2_), 7.72 (d, *J* = 8.8 Hz, 2H, PhSO_2_), 9.96 (s, 1H, SO_2_NH); ^13^C NMR (125 MHz, DMSO-*d*_6_) δ 112.2, 119.0, 119.2, 119.5, 119.8, 124.0, 124.3, 126.7, 127.5, 128.4, 129.1, 130.3, 136.9, 143.4, 150.3, 156.7 ppm; anal. C 67.67, H 4.65, N 7.17% calcd. for C_22_H_18_N_2_O_3_S, C 67.66, H 4.54, N 7.27%.

*N-[2-(Phenylthio)pyridin-3-y]-4-(1H-pyrrol-1-yl)benzenesulfonamide* (**26**). *Method A.* Starting from 4-amino-*N*-[2-(phenylthio)pyridin-3-y]benzenesulfonamide **15** (518 mg), the title compound **26** was obtained (236 mg, 40%): m.p. 113.4‒113.8 °C; IR (KBr) 3253 (NH), 1598 (NH _def_), 1510, 1475, 1439 (C=N, C=C), 1337 (SO_2 asym_), 1160 (SO_2 sym_) cm^−1^; ^1^H NMR (500 MHz, DMSO-*d*_6_) δ 6.33 (t, 2H, H-3 and H-4, pyrrole), 7.15 (dd, *J* = 8.1, 4.7 Hz, 1H, arom.), 7.20 (d, *J* = 7.8 Hz, 2H, arom.), 7.27‒7.32 (m, 3H, arom.), 7.39 (d, *J* = 8.3 Hz, 1H, arom.), 7.51 (t, 2H, H-2 and H-5, pyrrole), 7.76 (d, *J* = 8.8 Hz, 2H, PhSO_2_), 7.81 (d, *J* = 8.8 Hz, 2H, PhSO_2_), 8.17 (d, *J* = 4.9 Hz, 1H, arom.), 10.19 (s, 1H, SO_2_NH) ppm; anal. C 61.89, H 4.20, N 10.31% calcd. for C_21_H_17_N_3_O_2_S_2_, C 61.71, H 4.13, N 10.23%.

*N-(2-Phenoxypyridin-3-yl)-4-(1H-pyrrol-1-yl)benzenesulfonamide* (**27**). *Method A.* Starting from 4-amino-*N*-(2-phenoxypyridin-3-yl)benzenesulfonamide **16** (495 mg), the title compound **27** was obtained (369 mg, 65%): m.p. 163.7‒165.8 °C; IR (KBr) 3295 (NH), 1598 (NH _def_), 1512, 1489, 1451 (C=N, C=C), 1336 (SO_2 asym_), 1167 (SO_2 sym_) cm^−1^; ^1^H NMR (500 MHz, DMSO-*d*_6_) δ 6.33 (t, 2H, H-3 and H-4, pyrrole), 6.72 (d, *J* = 7.8 Hz, 2H, arom.), 7.07‒7.13 (m, 2H, arom.), 7.23 (t, 2H, arom.), 7.46 (t, 2H, H-2 and H-5, pyrrole), 7.73‒7.80 (m, 5H, arom.), 7.86 (dd, *J* = 4.9, 1.5 Hz, 1H, arom.), 10.23 (s, 1H, SO_2_NH); ^13^C NMR (125 MHz, DMSO-*d*_6_) δ 112.1, 119.5, 119.6, 119.7, 121.6, 121.6, 124.9, 129.0, 129.7, 135.4, 136.4, 143.4, 144.2, 153.7, 156.7 ppm; anal. C 64.43, H 4.38, N 10.73% calcd. for C_21_H_17_N_3_O_3_S, C 64.09, H 4.41, N 10.66%.

*4-(1H-Pyrrol-1-yl)-N-(quinolin-8-yl)benzenesulfonamide* (**28**). *Method A.* Starting from 4-amino-*N*-(quinolin-8-yl)benzenesulfonamide **17** (434 mg), the title compound **28** was obtained (258 mg, 51%): m.p. 151.9‒153.0 °C dec.; IR (KBr) 3254 (NH), 1597 (NH _def_), 1505, 1471 (C=N, C=C), 1335 (SO_2 asym_), 1163 (SO_2 sym_) cm^−1^; ^1^H NMR (500 MHz, DMSO-*d*_6_) δ 6.26 (t, 2H, H-3 and H-4, pyrrole), 7.41 (t, 2H, H-2 and H-5, pyrrole), 7.53 (t, 1H, arom.), 7.56 (dd, *J* = 8.3, 3.9 Hz, 1H, arom.), 7.66 (d, *J* = 8.3 Hz, 1H, arom.), 7.69 (d, *J* = 8.8 Hz, 2H, PhSO_2_), 7.72 (d, *J* = 7.9 Hz, 1H, arom.), 7.96 (d, *J* = 8.8 Hz, 2H, PhSO_2_), 8.33 (dd, *J* = 8.3, 2 Hz, 1H, arom.), 8.86 (dd, *J* = 4.4, 1.9 Hz, 1H, arom.), 10.06 (s, 1H, SO_2_NH); ^13^C NMR (125 MHz, DMSO-*d*_6_) δ 112.9, 117.3, 119.3, 119.5, 122.8, 123.6, 127.2, 128.6, 129.3, 134.1, 135.7, 136.0, 139.3, 143.4, 149.9 ppm; anal. C 65.31, H 4.33, N 12.03% calcd. for C_19_H_15_N_3_O_2_S, C 65.07, H 4.20, N 12.00%.

*N-(2-Methylquinolin-8-yl)-4-(1H-pyrrol-1-yl)benzenesulfonamide* (**29**). *Method A.* Starting from 4-amino-*N*-(2-methylquinolin-8-yl)benzenesulfonamide **18** (454 mg), the title compound **29** was obtained (316 mg, 60%): m.p. 167.6‒168.7 °C; IR (KBr) 3225 (NH), 1596 (NH _def_), 1506, 1472 (C=N, C=C), 1327 (SO_2 asym_), 1166 (SO_2 sym_) cm^−1^; ^1^H NMR (500 MHz, DMSO-*d*_6_) δ 2.66 (s, 3H, CH_3_), 6.27 (t, 2H, H-3 and H-4, pyrrole), 7.40‒7.46 (m, 4H, arom. and H-2 and H-5, pyrrole), 7.60 (d, *J* = 7.8 Hz, 1H, arom.), 7.64 (d, *J* = 7.3 Hz, 1H, arom.), 7.69 (d, *J* = 8.8 Hz, 2H, PhSO_2_), 7.94 (d, *J* = 8.8 Hz, 2H, PhSO_2_), 8.20 (d, *J* = 8.8 Hz, 1H, arom.), 9.83 (s, 1H, SO_2_NH) ppm; anal. C 66.10, H 4.71, N 11.56% calcd. for C_20_H_17_N_3_O_2_S, C 66.02, H 4.69, N 11.56%.

*N-(7-Methylquinolin-8-yl)-4-(1H-pyrrol-1-yl)benzenesulfonamide* (**30**). *Method A.* Starting from 4-amino-*N*-(7-methylquinolin-8-yl)benzenesulfonamide **19** (454 mg), the title compound **30** was obtained (343 mg, 65%): m.p. 142‒145 °C; IR (KBr) 3235 (NH), 1599 (NH _def_), 1516, 1503, 1466 (C=N, C=C), 1335 (SO_2 asym_), 1167 (SO_2 sym_) cm^−1^; ^1^H NMR (500 MHz, DMSO-*d*_6_) δ 2.59 (s, 3H, CH_3_), 6.29 (t, 2H, H-3 and H-4, pyrrole), 7.29 (dd, *J* = 8.3, 3.9 Hz, 1H, arom.), 7.41 (t, 2H, H-2 and H-5, pyrrole), 7.53 (d, *J* = 8.3 Hz, 1H, arom.), 7.56 (br. s, 4H, arom.), 7.78 (d, *J* = 8.3 Hz, 1H, arom.), 8.21 (d, *J* = 8.3 Hz, 1H, arom.), 8.39 (d, *J* = 3.9 Hz, 1H, arom.), 9.74 (s, 1H, SO_2_NH); ^13^C NMR (125 MHz, DMSO-*d*_6_) δ 19.9, 111.9, 118.5, 119.5, 121.1, 127.0, 127.1, 129.3, 130.3, 131.5, 136.2, 137.3, 138.9, 142.5, 144.5, 149.7 ppm; anal. C 66.10, H 4.71, N 11.56% calcd. for C_20_H_17_N_3_O_2_S, C 65.82, H 4.68, N 11.51%.

*N-(2-Methylquinolin-6-yl)-4-(1H-pyrrol-1-yl)benzenesulfonamide* (**31**). *Method B.* Starting from 4-amino-*N*-(2-methylquinolin-6-yl)benzenesulfonamide **20** (439 mg), the title compound **31** was obtained (280 mg, 55%): m.p. 286‒288 °C; IR (KBr) 3265, 3140 (SO_2_NH), 1596 (NH _def_), 1510, 1475 (C=N, C=C), 1340 (SO_2 asym_), 1160 (SO_2 sym_) cm^−1^; ^1^H NMR (500 MHz, DMSO-*d*_6_) δ 2.57 (s, 3H, CH_3_), 6.26 (br. s, 2H, H-3 and H-4, pyrrole), 7.33 (d, *J* = 6.8 Hz, 1H, H-3, quinoline), 7.42-7.46 (m, 3H, arom.), 7.59 (s, 1H, H-5, quinoline), 7.73-7.96 (m, 5H, arom.), 8.14 (d, *J* = 6.4 Hz, 1H, H-8, quinoline), 10.58 (s, 1H, SO_2_NH) ppm; ^13^C NMR (125 MHz, DMSO-*d*_6_) δ 24.7, 111,9, 116.3, 119.3, 122.9, 124.0, 126.7, 128.8, 129.6, 135.4, 143.1, 144.7, 158.0 ppm; anal. C 66.09, H 4.71, N 11.56% calcd. for C_20_H_17_N_3_O_2_S, C 66.12, H 4.81, N 11.55%.

*4-(1H-pyrrol-1-yl)-N-(4H-1,2,4-triazol-4-yl)benzenesulfonamide* (**32**). *Method B.* Starting from 4-amino-*N*-(4*H*-1,2,4-triazol-4-yl)benzenesulfonamide **21** (335 mg), the title compound **32** was obtained (219 mg, 54%): m.p. 251‒252 °C dec.; IR (KBr) 3150 (NH), 1595 (NH _def_), 1515, 1475, 1425 (C=N, C=C), 1340 (SO_2 asym_), 1165 (SO_2 sym_) cm^−1^; ^1^H NMR (500 MHz, DMSO-*d*_6_) δ 6.35 (br. s, 2H, H-3 and H-4, pyrrole), 7.57 (br. s, 2H, H-2 and H-5, pyrrole), 7.70 (d, *J* = 8.3 Hz, 2H, H-3 and H-5, PhSO_2_), 7.88 (d, *J* = 8.3 Hz, 2H, H-2 and H-6, PhSO_2_), 8.37 (s, 2H, H-3 and H-5, 1,2,4-triazole), 11.90 (br. s, 1H, SO_2_NH) ppm; ^13^C NMR (DMSO-*d*_6_) δ 112.7, 119.9, 120.0, 130.4, 143.5, 144.7 ppm; anal. C 49.82, H 3.83, N 24.20% calcd. for C_12_H_11_N_5_O_2_S, C 49.80, H 3.93, N 24.19%.

*N-(Benzo-2,1,3-thiadiazol-4-yl)-4-(1H-pyrrol-1-yl)benzenesulfonamide* (**33**). *Method C.* Starting from 4-amino-*N*-(benzo-2,1,3-thiadiazol-4-yl)benzenesulfonamide **22** (444 mg), the title compound **33** was obtained (336 mg, 65%): m.p. 325‒327 °C dec.; IR (KBr) 3320, 3225 (NH), 1595 (NH _def_), 1540, 1510, 1495 (C=N, C=C), 1340 (SO_2 asym_), 1170 (SO_2 sym_) cm^−1^; ^1^H NMR (220 MHz, DMSO-*d*_6_) δ 6.30 (br. s, 2H, H-3 and H-4, pyrrole), 7.46 (br. s, 2H, H-2 and H-5, pyrrole), 7.64 (t, *J* = 7.8 Hz, 1H, H-6, benzothiadizole), 7.68-7.80 (m, 4H, H-3 and H-5, PhSO_2_ and H-5 and H-7 benzothiadiazole), 7.90 (d, *J* = 8.0 Hz, 2H, H-2 and H-6 PhSO_2_), 11.0 (br. s, 1H, SO_2_NH) ppm; ^13^C NMR (DMSO-*d*_6_) δ 111.9, 117.2, 118.1, 119.2, 119.4, 129.0, 129.7, 130.7, 136.0, 143.2, 149.0, 155.1 ppm; anal. C 53.91, H 3.39, N 15.72% calcd. for C_16_H_12_ N_4_O_2_S_2_, C 54.02, H 3.48, N 15.81%.

*N-(5-Methylisoxazol-3-yl)-4-(1H-pyrrol-1-yl)-benzenesulfonamide* (**34**). *Method A.* Starting from 4-amino-*N*-(5-methylisoxazol-3-yl)benzenesulfonamide **I** (367 mg), the title compound **34** was obtained (387 mg, 88%): m.p. 203‒204.8 °C; IR (KBr) 3235 (NH), 1599 (NH _def_), 1516, 1503, 1466 (C=N, C=C), 1337 (SO_2 asym_), 1168 (SO_2 sym_) cm^−1^; ^1^H NMR (500 MHz, DMSO-*d*_6_) δ 2.29 (s, 3H, CH_3_), 6.17 (s, 1H, arom.), 6.32 (s, 2H, H-3 and H-4, pyrrole), 7.49 (s, 2H, H-2 and H-5, pyrrole), 7.82 (d, *J* = 8.8 Hz, 2H, H-2 and H-6 PhSO_2_), 7.89 (d, *J* = 8.8 Hz, 2H, H-2 and H-6 PhSO_2_.), 11.46 (s, 1H, SO_2_NH); ^13^C NMR (125 MHz, DMSO-*d*_6_) δ 12.7, 96.1, 112.4, 119.8, 119.9, 129.3, 135.9, 143.9, 158.2, 171.2 ppm; anal. C 55.43, H 4.32, N 13.85% calcd. for C_14_H_13_N_3_O_3_S, C 54.96, H 4.24, N 13.75%.

*N-(Pyridin-2-yl)-4-(1H-pyrrol-1-yl)benzenesulfonamide* (**35**). *Method A.* Starting from 4-amino-*N*-(pyridin-2-yl)benzenesulfonamide **II** (361 mg), the title compound **35** was obtained (391 mg, 90%): m.p. 244‒246.4 °C; IR (KBr) 3138 (NH), 1634 (NH _def_), 1535, 1512, 1466 (C=N, C=C), 1359 (SO_2 asym_), 1142 (SO_2 sym_) cm^−1^; ^1^H NMR (500 MHz, DMSO-*d*_6_) δ 6.29 (s, 2H, H-3 and H-4, pyrrole), 6.86 (t, 1H, arom.), 7.17 (d, 1H, arom.), 7.45 (s, 2H, H-2 and H-5, pyrrole), 7.71 (d, 1H, arom.), 7.74 (d, *J* = 8.8 Hz, 2H, H-2 and H-6 PhSO_2_), 7.90 (d, *J* = 8.8 Hz, 2H, H-2 and H-6 PhSO_2_.), 8.00 (d, 1H, arom.), 12.06 (s, 1H, SO_2_NH); ^13^C NMR (125 MHz, DMSO-*d*_6_) δ 112.2, 114.6, 116.1, 119.6, 119.8, 129.0, 138.8, 141.4, 143.0, 153.9 ppm; anal. C 60.18, H 4.38, N 14.04% calcd. for C_15_H_13_N_3_O_2_S, C 60.00, H 4.19, N 13.88%.

*N-(Pyrimidin-2-yl)-4-(1H-pyrrol-1-yl)benzenesulfonamide* (**36**). *Method C.* Starting from 4-amino-*N*-(pyrimidin-2-yl)benzenesulfonamide **III** (363 mg), the title compound **36** was obtained (379 mg, 87%): m.p. 260‒261°C dec.; IR (KBr) 3150 (NH), 1600 (NH _def_), 1585, 1510, 1445 (C=N, C=C), 1345 (SO_2 asym_), 1160 (SO_2 sym_) cm^−1^; ^1^H NMR (200 MHz, DMSO-*d*_6_) δ 6.30 (br. s, 2H, H-3 and H-4, pyrrole), 7.04 (t, *J* = 4.9 Hz, 1H, H-5, pyrimidine), 7.48 (br. s, 2H, H-2 and H-5, pyrrole), 7.85 (d, *J* = 8.8 Hz, 2H, H-3 and H-5, PhSO_2_), 8.01 (d, *J* = 8.8 Hz, 2H, H-2 and H-6, PhSO_2_), 8.50 (d, *J* = 4.9 Hz, 2H, H-4 and H-6, (pyrimidine), 11.88 (br. s, 1H, SO_2_NH) ppm; ^13^C NMR (50 MHz, DMSO-*d*_6_) δ 112.3, 119.4, 119.8, 130.1, 136.9, 143.5, 157.5, 159.0 ppm; anal. C 55.99, H 4.02, N 18.65% calcd. for C_14_H_12_N_4_O_2_S, C 56.20, H 4.22, N 18.72%.

*N-(4-Methylpyrimidin-2-yl)-4-(1H-pyrrol-1-yl)benzenesulfonamide* (**37**). *Method C.* Starting from 4-amino-*N*-(4-methylpyrimidin-2-yl)benzenesulfonamide **IV** (383 mg) the title compound 3**7** was obtained (392 mg, 86%): m.p. 256‒257 °C dec.; IR (KBr) 3235 (NH), 1600 (NH _def_), 1565, 1510, 1405 (C=N, C=C), 1345 (SO_2 asym_), 1150 (SO_2 sym_) cm^−1^; ^1^H NMR (200 MHz, DMSO-*d*_6_) δ 2.31 (s, 3H CH_3_), 6.30 (br. s, 2H, H-3 and H-4, pyrrole), 6.90 (d, *J* = 4.9 Hz, 1H, H-5, pyrimidine), 7.47 (br. s, 2H, H-2 and H-5, pyrrole), 7.77 (d, *J* = 8.3 Hz, 2H, H-3 and H-5, PhSO_2_), 8.01 (d, *J* = 8.3 Hz, 2H, H-2 and H-6, PhSO_2_), 8.32 (d, *J* = 4.9 Hz, 1H, H-6, pyrimidine), 11.82 (br. s, 1H, SO_2_NH) ppm; ^13^C NMR (50 MHz, DMSO-*d*_6_) δ 23.9, 112.3, 119.2, 119.8, 130.3, 137.2, 143.4, 157.2 ppm; anal. C 57.31, H 4.49, N 17.82% calcd. for C_15_H_14_N_4_O_2_S, C 57.45, H 4.66, N 17.90%.

*N-(4,6-Dimethylpyrimidin-2-yl)-4-(1H-pyrrol-1-yl)benzenesulfonamide* (**38**). *Method C.* Starting from 4-amino-*N*-(4,6-dimethylpyrimidin-2-yl)benzenesulfonamide **V** (404 mg), the title compound **38** was obtained (376 mg, 79%): m.p. 224‒227 °C dec.; IR (KBr) 3245 (NH), 1600 (NH _def_), 1585, 1510, 1475 (C=N, C=C), 1335 (SO_2 asym_), 1160 (SO_2 sym_) cm^−1^; ^1^H NMR (200 MHz, DMSO-*d*_6_) δ 2.24 (s, 6H, 2 × CH_3_), 6.29 (br. s, 2H, H-3 and H-4, pyrrole), 6.74 (s, 1H, H-5, pyrimidine), 7.46 (br. s, 2H, H-2 and H-5, pyrrole), 7.76 (d, *J* = 8.3 Hz, 2H, H-3 and H-5, PhSO_2_), 8.02 (d, *J* = 8.3 Hz, 2H, H-2 and H-6, PhSO_2_), 11.86 (br. s, 1H, SO_2_NH) ppm; ^13^C NMR (50 MHz, DMSO-*d*_6_) δ 23.5, 112.2, 119.0, 119.8, 130.5, 143.2, 156.8 ppm; anal. C 58.51, H 4.91, N 17.06% calcd. for C_16_H_16_N_4_O_2_S, C 58.85, H 5.30, N 17.52%.

*N-(2,6-Dimethoxypirimidin-4-yl)-4-(1H-pyrrol-1-yl)benzenesulfonamide* (**39**). *Method C.* Starting from 4-amino-*N*-(2,6-dimethoxypirimidin-4-yl)benzenesulfonamide **VI** (450 mg) the title compound **39** was obtained (423 mg, 81%): m.p. 162‒163 °C dec.; IR (KBr) 3240, 3150 (NH), 1595 (NH _def_), 1510, 1490, 1455 (C=N, C=C), 1335 (SO_2 asym_), 1160 (SO_2 sym_) cm^−1^; ^1^H NMR (200 MHz, DMSO-*d*_6_) δ 3.78 (s, 3H CH_3_O, pyrimidine), 3.81 (s, 3H, CH_3_O-2, pyrimidine), 5.99 (s, 1H, H-5, pyrimidine), 6.34 (br. s, 2H, H-3 and H-4, pyrrole), 7.51 (br. s, 2H, H-2 and H-5, pyrrole), 7.84 (d, *J* = 8.4 Hz, 2H, H-3 and H-5, PhSO_2_), 8.00 (d, *J* = 8.4 Hz, 2H, H-2 and H-6, PhSO_2_), 11.86 (br. s, 1H, SO_2_NH) ppm; ^13^C NMR (50 MHz, DMSO-*d*_6_) δ 54.1, 54.8, 85.0, 112.0, 119.3, 119.5, 129.3, 136.0, 143.4, 160.1, 164.2, 171.9 ppm; anal. C 53.32, H 4.47, N 15.54% calcd. for C_16_H_16_N_4_O_4_S, C 53.48, H 4.60, N 15.70%.

*N-(6-Methoxypyridazin-3-yl)-4-(1H-pyrrol-1-yl)benzenesulfonamide* (**40**). *Method B.* Starting from 4-amino-*N*-(6-methoxypyridazin-3-yl)benzenesulfonamide **VII** (392 mg), the title compound **40** was obtained (241 mg, 52%): 191‒192 °C; IR (KBr) 3215 (NH), 1645 (NH _def_), 1525, 1510, 1470 (C=N, C=C), 1335 (SO_2 asym_), 1145 (SO_2 sym_) cm^−1^; ^1^H NMR (200 MHz, DMSO-*d*_6_) δ 3.83 (s, 3H, CH_3_O), 6.30 (br. s, 2H, H-3 and H-4, pyrrole), 7.37 (d, *J* = 7.9 Hz, 1H, H-5, pyridazine), 7.48 (br. s, 2H, H-2 and H-5, pyrrole), 7.72‒7.81 (m, 3H, H-3 and H-5, PhSO_2_, and H-4, pyridazine), 7.87 (d, *J* = 8.6 Hz, 2H, H-2 and H-6, PhSO_2_), 13.00 (br. s, 1H, SO_2_NH) ppm; ^13^C NMR (50 MHz, DMSO-*d*_6_) δ 53.54, 110.9, 117.8, 118.5, 119.2, 125.0, 128.2, 140.5, 144.3, 158.7, 159.9 ppm; anal. C 54.53, H 4.27, N 16.96% calcd. for C_15_H_14_N_4_O_3_S, C 54.52, H 4.30, N 16.95%.

### 3.3. X-ray Structure Determination

X-Ray diffraction data were collected on the IPDS 2T dual-beam diffractometer (STOE & Cie GmbH, Darmstadt, Germany) at 120.0(2) K with Cu-Kα radiation of a microfocus X-ray source (GeniX 3D Cu High Flux, Xenocs, Sassenage, France, 50 kV, 0.6 mA, λ = 154.186 pm). During the experiment, the crystal was kept in nitrogen stream at 120 K using CryoStream-800 device (Oxford CryoSystem, Oxford, UK). Control of data collection and data reduction was performed by X-Area 1.75 program [35]. An absorption correction was based on the integrated reflections using a combination of reflection scaling, frame scaling, and a spherical absorption correction. Blessing’s method was used to reject outliers [36]. Numerical absorption correction was performed after the optimization of the crystal-shape description by Herrendorf’s method [37].

The structure was solved using direct methods with SHELXS-13 program and refined by SHELXL-2013 [38] program run under control of WinGx [39]. Positions of the C-H hydrogen atoms were calculated geometrically and taken into account with isotropic temperature factors. The NH hydrogen atom was found in the Fourier residual electron density map and was refined without constraints. 

Crystallographic data for structure of **25** has been deposited with the Cambridge Crystallographic Data Centre, No. CCDC 1825991. Copies of this information can be obtained free of charge on application to CCDC, 12 Union Road, Cambridge, CB21EZ, UK (Fax: +44-1223-336033; E-mail: deposit@ccdc.cam.ac.uk or Available online: http://www.ccdc.cam.ac.uk).

### 3.4. Cell Culture and Cell Viability Assay

All chemicals were purchased from Sigma-Aldrich (St. Louis, MO, USA). The MCF-7 and HeLa cell lines were obtained from Cell Lines Services (Eppelheim, Germany), and the HCT-116 cell line was ordered from ATCC (ATCC-No: CCL-247). Cells were cultured in Dulbecco’s modified Eagle’s medium (DMEM). Medium was supplemented with 10% fetal bovine serum, 100 units/mL penicillin, 2 mM glutamine, and 100 μg/mL streptomycin. Cultures were held in an incubator (HeraCell, Heraeus, Langenselbold, Germany) in a humidified atmosphere with 5% CO_2_ at 37 °C.

***Screening for cytotoxic activity:*** The MTT (3-(4,5-dimethylthiazol-2-yl)-2,5-diphenyltetrazoliumbromide) assay was used to determine cell viability. Stock solutions of different compounds were obtained by dissolving them in 100% DMSO. To prepare working solutions samples, stock solutions were diluted with DMEM medium. The contents of DMSO in the treated samples did not exceed 0.5%. 96-Well plates were inoculated with cells at a density of 5 × 10^3^ cells/well; then, medium containing the test compound at different concentrations (1, 10, 25, 50, and 100 μM) was added to each well and incubated for 72 h. After treatment, cells were incubated for 2 h with MTT (0.5 mg/mL) at 37 °C. Subsequently, cells were lysed with DMSO, and absorbance of the formazan solution was measured at 550 nm (1420 multilabel counter, Victor, Jügesheim, Germany). Data were expressed as the mean ± SD of at least three independent experiments carried out in triplicate.

***Time-dependent cytotoxicity assay:*** Viability of cells was evaluated using the MTT (3-(4,5-dimethylthiazol-2-yl)-2,5-diphenyltetrazoliumbromide) assay. Cells were seeded in 96-well plates at a density of 5 × 10^3^ cells/well and treated for 24, 48, and 72 h with the examined compound in the concentration range 1–25 μM. The following steps of the experiment were carried out as indicated above in *Screening for cytotoxic activity* section.

***Detection of apoptosis by Annexin V-PE and 7-AAD staining:*** Apoptosis induction was detected with an Annexin V-PE Apoptosis Detection Kit I (BD Biosciences, Erembodegem, Belgium) according to the manufacturer’s instructions. Cells were treated with **28** (2.5, 5, and 10 µM) for 48 h (HCT-116) and 72 h (HeLa and MCF-7). Following treatment cells were collected, washed with Annexin-binding buffer, and stained with Annexin V-phycoerythrin (PE) and 7-amino-actinomycin (7-AAD) for 15 min at RT in the dark. Acquisition was performed on a FACSCalibur cytometer (BD) and data were analyzed with Flowing software (version 2.5) (Turku, Finland).

***Caspase Activity Determination:*** The FLICA Apoptosis Detection Kit (Immunochemistry Technologies, Bloomington, IN, USA) was used to determined caspase activity. Cultured cells were treated with **28** (2.5, 5, and 10 µM) for 24 h (HCT-116) and 48 h (HeLa and MCF-7). After collection of cells, the caspase inhibitor—a carboxyfluorescein-labeled fluoromethyl ketone peptide—was added. Cells were incubated at 37 °C in a humidified 5% CO_2_ incubator for 1 h and washed with washing buffer. Flow cytometry (BD FACSCalibur, BD Bioscience, San Jose, CA, USA) was used to determine the amount of fluorescence emitted from inhibitors bound with the caspases corresponding to their activity.

***Cell Cycle Distribution Analysis:*** The effects of **28** on cell cycle distribution in HCT-116, HeLa, and MCF-7 cells were determined with flow cytometry analysis. Cells were treated with **28** (2.5, 5, and 10 µM) for 48 h (HCT-116) and 72 h (HeLa and MCF-7), after which they were fixed in cold 70% ethanol for 24 h. Fixed cells were treated with 100 µg/mL RNAse (Invitrogen, Darmstadt, Germany) and stained with 10 µg/mL PI (Invitrogen) for 30 min at RT. Acquisition was performed on a FACSCalibur cytometer (BD), and data were analyzed with Flowing software (version 2.5).

### 3.5. Molecular Modeling and QSAR Study

In order to find the lowest energy geometry of the studied compounds, before starting molecular modeling algorithms, the two-dimensional structure of molecules were created using of Gaussian software (Gaussian G09, v D.01, Gaussian Inc., Wallingford, CT, USA). The obtained structures were optimized by means of density functional theory (DFT) algorithm using B3LYP/6-31G(d) basis set. 

The developed three-dimensional structures were submitted to descriptor calculations using Dragon software (Dragon v. 7.0.6, Kode srl, Pisa, Italy). Only a selected block of descriptors were calculated: constitutional descriptors, ring descriptors, functional group counts, atom-centered fragments, CASTS2D descriptors, 2D atom pairs, charge descriptors, and molecular properties, in order to enable easier interpretation of the obtained models based on chemical structure of the proposed benzenesulfonamides. 

Activity dataset was evaluated before QSAR analysis in terms of the presence of possible outliers. OPLS calculations, as well as a validation study, were performed using SIMCA (SIMCA v. 13.0.3.0, Umeå, Sweden). Descriptors were used as independent variables, and IC_50_ was used as a dependent variable. 

## 4. Conclusions

We have synthesized a series of *N*-(aryl/heteroaryl)-4-(1*H*-pyrrol-1-yl)benzenesulfonamide derivatives from 4-amino-*N*-(aryl/heteroaryl)benzenesulfonamides and 2,5-dimethoxytetrahydrofuran. The molecular structures of novel compounds were confirmed by elemental analyses and NMR and IR spectroscopic methods. For representative compound **25**, an X-ray structure was determined. 

All compounds were tested for their in vitro cytotoxic activity against HCT-116, HeLa, and MCF-7 cell lines. It has been found that compounds bearing a quinolin-8y-l moiety showed the best anticancer potential. The most prominent compound **28** exhibited significant cytotoxic activity with IC_50_ values of 3, 5, and 7 µM for HCT-116, MCF-7, and HeLa, respectively. Due to high cytotoxic activity, compound **28** was investigated for apoptosis-inducing activity in cancer cells. Appearance of an increased population of early and late apoptotic cells, induction of caspase activity, and cell cycle arrest at G2/M phase confirmed apoptosis of HCT-116, HeLa, and MCF-7 cells in the presence of compound **28**.

The applied QSAR approach based on OPLS and corresponding VIP values allowed one to recognize chemical sub-structures related to increased or decreased biological activity. Especially, more rigid Ar/Het substituents are beneficial for activity. We also observed decreased activity related with the presence of oxygen-containing substituents. Those indications can be used for a rational plan of further synthesis. 

## Figures and Tables

**Table 1:** Crystal data and structure refinement details for **25**.

Empirical Formula	C_22_H_18_N_2_O_3_S
Formula weight	390.44
Temperature/K	120 (2)
Wavelength/Å	1.54186
Crystal system	monoclinic
Space group	*P*2_1_/*n*
Unit cell dimensions	
*a*/Å	10.0024 (16)
*b*/Å	5.4137 (9)
*c*/Å	32.784 (5)
*α*/°	90
*β*/°	92.318 (12)
*γ*/°	90
Volume/Å^3^	1773.8 (5)
*Z*	4
Density (calculated)/Mgm^−^^3^	1.462
Absorption coefficient/mm^−1^	1.85
*F*(000)	816
Crystal size/mm^3^	0.041 × 0.12 × 0.58
*θ* range for data collection/°	4.7–66.7
Index ranges	−8 ≤ h ≤ 11, −6 ≤ k ≤ 6, −35 ≤ l ≤ 38
Reflections collected	10,220
Independent reflections	3004, (*R*_int_ = 0.0495)
Completeness to *θ* = 66.69°	98.2%
Absorption correction	Integration
Refinement method	Full-matrix least-squares on *F*^2^
Data/restraints/parameters	3004/0/257
Goodness of fit on *F*^2^	1.024
Final *R* indices [I > 2σ(I)]	*R*_1_ = 0.0410, wR_2_ = 0.1171
*R* indices (all data)	*R*_1_ = 0.0461, wR_2_ = 0.1246
Largest diff. peak and hole/e Å^−3^	0.54, −0.49

**Table 2:** Hydrogen-bond geometry (Å, °) for **25**.

*D*–H···*A*	*D*–H	H···*A*	*D*···*A*	*D*–H···*A*
N1–H1···O3 ^i^	0.81 (2)	2.38 (2)	3.190 (2)	174 (2)
C14–H14···O1 ^ii^	0.95	2.42	3.296 (2)	153

Symmetry codes: ^i^ *x*, *y* + 1, *z*; ^ii^ *x*, *y* − 1, *z*.

**Table 3:** IC_50_ values for compounds **23**–**40** assessed by the MTT tests ^a^.

Compound	IC_50_ (μM)		
	HCT-116	HeLa	MCF-7
**23**	71 ± 1.4	73 ± 1	82 ± 2
**24**	140 ± 3	125 ± 2	140 ± 8
**25**	145 ± 4	145 ± 6	180 ± 9
**26**	*	780 ± 15	*
**27**	175 ± 4	220 ± 2	320 ± 10
**28**	**3 ± 0.1**	**7 ± 0.2**	**5 ± 0.2**
**29**	**9 ± 0.1**	**25 ± 1**	93 ± 5
**30**	**19 ± 0.5**	**20 ± 0.2**	**50 ± 2**
**31**	105 ± 2	190 ± 6	220 ± 9
**32**	380 ± 22	*	170 ± 10
**33**	**23 ± 0.2**	**42 ± 2**	**23 ± 1**
**34**	*	*	275 ± 14
**35**	135 ± 6	115 ± 3	140 ± 4
**36**	*	*	*
**37**	**46 ± 2**	62 ± 1	68 ± 3
**38**	69 ± 1	83 ± 2	135 ± 4
**39**	330 ± 3	340 ± 17	410 ± 8
**40**	390 ± 11	480 ± 19	*

^a^ Analysis was performed using the MTT assay after 72 h of incubation. Values are expressed as the mean ± SD of at least three independent experiments. Cytotoxic activity values below 50 µM is bolded. * Viability of cell lines at 100 μM of tested compounds was approximately 100%.

## Supplementary Materials

Supplementary materials can be accessed online at http://www.mdpi.com/1422-0067/19/5/1482/s1.
