# Supplementary material for: Synthesis, Molecular Structure, Anticancer Activity, and QSAR Study of N-(aryl/heteroaryl)-4-(1H-pyrrol-1-yl)Benzenesulfonamide Derivatives
Source: Int J Mol Sci. 2018 May 16;19(5):1482. doi: 10.3390/ijms19051482 (PMC5983619; doi:10.3390/ijms19051482)
Supplement: Supplementary file 1 [file ijms-19-01482-s001.pdf]

Synthesis, molecular structure, anticancer activity and QSAR study of *N*-(aryl/heteroaryl)-4-(1*H*-pyrrol-1-yl)benzenesulfonamide derivatives

Beata Żołnowska [1*], Jarosław Sławiński [1], Zdzisław Brzozowski [1], Anna Kawiak [2,3], Mariusz Belka [4], Joanna Zielińska [4], Tomasz Bączek [4] and Jarosław Chojnacki [5]**

[1] Department of Organic Chemistry, Medical University of Gdańsk, Al. Gen. J. Hallera 107, 80-416 Gdańsk, Poland; brzozowskigumed@gmail.com (Z.B.)

[2] Department of Biotechnology, Intercollegiate Faculty of Biotechnology, University of Gdańsk and Medical University of Gdańsk, ul. Abrahama 58, 80-307 Gdańsk, Poland; kawiak@biotech.ug.edu.pl (A.K.)

[3] Laboratory of Human Physiology, Medical University of Gdańsk, ul. Tuwima 15, 80-210 Gdańsk, Poland

[4] Department of Pharmaceutical Chemistry, Medical University of Gdańsk, Al. Gen. J. Hallera 107, 80-416 Gdańsk, Poland; mariusz.belka@gumed.edu.pl (M.B.); joanna.zielinska@gumed.edu.pl (J.Z.); tomasz.baczek@gumed.edu.pl (T.B.)

[5] Department of Inorganic Chemistry, Gdańsk University of Technology, ul. Narutowicza 11/12, 80-233 Gdańsk, Poland; jarekch@pg.gda.pl (J.Ch.)

* Correspondence: beata.zolnowska@gumed.edu.pl (B.Ż.); jaroslaw.slawinski@gumed.edu.pl (J.S.); Tel.: +48-58-349-1098; +48-58-349-1229 (B.Ż. & J.S.); Fax: +48-58-349-1277 (B.Ż. & J.S.)

Table of contents

Table S1. The most influential molecular descriptors based on VIP values derived from OPLS models

Table S2. Values of descriptors characterized by high VIP values for OPLS model for HCT-116 cell line. Table is sorted with descending activity towards HCT-116.

Table S3. Values of descriptors characterized by high VIP values for OPLS model for HeLa cell line. Table is sorted with descending activity towards HeLa.

Table S4. Values of descriptors characterized by high VIP values for OPLS model for MCF-7 cell line. Table is sorted with descending activity towards MCF-7.

Spectrum 1. ^{1}H NMR of compd **3** (500 MHz, DMSO-d_6).

Spectrum 2. ^{13}C NMR of compd **3** (125 MHz, DMSO-d_6).

Spectrum 3. ^{1}H NMR of compd **4** (500 MHz, DMSO-d_6).

Spectrum 4. ^{13}C NMR of compd **4** (125 MHz, DMSO-d_6).

Spectrum 5. ^{1}H NMR of compd **5** (500 MHz, DMSO-d_6).

Spectrum 6. ^{13}C NMR of compd **5** (125 MHz, DMSO-d_6).

Spectrum 7. ^{1}H NMR of compd **8** (500 MHz, DMSO-d_6).

Spectrum 8. ^{13}C NMR of compd **8** (125 MHz, DMSO-d_6).

Spectrum 9. ^{1}H NMR of compd **16** (500 MHz, DMSO-d_6).

Spectrum 10. ^{13}C NMR of compd **16** (125 MHz, DMSO-d_6).

Spectrum 11. ^{1}H NMR of compd **19** (500 MHz, DMSO-d_6).

Spectrum 12. ^{13}C NMR of compd **19** (125 MHz, DMSO-d_6).

Spectrum 13. ^{1}H NMR of compd **23** (500 MHz, DMSO-d_6).

Spectrum 14. ^{13}C NMR of compd **23** (125 MHz, DMSO-d_6).

Spectrum 15. ^{1}H NMR of compd **25** (500 MHz, DMSO-d_6).

Spectrum 16. ^{13}C NMR of compd **25** (125 MHz, DMSO-d_6).

Spectrum 17. ^{1}H NMR of compd **27** (500 MHz, DMSO-d_6).

Spectrum 18. ^{13}C NMR of compd **27** (125 MHz, DMSO-d_6).

Spectrum 19. ^{1}H NMR of compd **30** (500 MHz, DMSO-d_6).

Spectrum 20. ^{13}C NMR of compd **30** (125 MHz, DMSO-d_6).

Table S1

The most influential molecular descriptors based on VIP values derived from OPLS models

Molecular descriptor	VIP value
**HCT-116**	
F08[C-O]	2.04000
RBF	2.00536
CATS2D_07_DL	1.96385
B09[C-O]	1.96385
F09[C-O]	1.96385
RBN	1.91183
NRS	1.91183
CATS2D_06_DL	1.91183
F03[C-N]	1.89564
CATS2D_08_AL	1.76264
RFD	1.73683
RCI	1.73683
Rbrid	1.73397
NNRS	1.73397
C-028	1.63053
**HeLa**	
CATS2D_07_DL	2.35529
B09[C-O]	2.35529
F09[C-O]	2.35529
F08[C-O]	2.17873
CATS2D_08_AL	2.01828
B08[C-S]	1.88821
RBN	1.75819
NRS	1.75819
CATS2D_06_DL	1.75819
**MCF-7**	
nO	2.30514
O-060	2.30514
T(O..O)	2.30514
B01[C-O]	2.30514
B04[O-S]	2.30514
B05[O-O]	2.30514
B09[N-O]	2.30514
B10[C-O]	2.30514
F04[O-S]	2.30514
F05[O-O]	2.30514
F09[N-O]	2.30514
F10[C-O]	2.30514
B03[N-O]	2.28874

Table S2

Values of descriptors characterized by high VIP values for OPLS model for HCT-116 cell line. Table is sorted with descending activity towards HCT-116.

ID	HCT116 IC50	F08[C-O]	RBF	CATS2D_07_DL	B09[C-O]	F09[C-O]	RBN	NRS	CATS2D_06_DL	F03[C-N]	CATS2D_08_AL	RFD	RCI	Rbrid	NNRS	C-028
**28**	4	4	0.093	0	0	0	4	3	0	9	5	0.095	1.095	1	0.75	1
**29**	9	4	0.087	0	0	0	4	3	0	9	5	0.095	1.095	1	0.75	2
**30**	19	4	0.087	0	0	0	4	3	0	10	5	0.095	1.095	1	0.75	1
**33**	23	4	0.103	0	0	0	4	3	0	10	7	0.1	1.1	1	0.75	2
**37**	46	4	0.105	0	0	0	4	3	0	8	4	0	1	0	1	1
**38**	69	4	0.098	0	0	0	4	3	0	8	4	0	1	0	1	2
**23**	71	4	0.108	0	0	0	4	3	0	6	6	0	1	0	1	0
**31**	105	6	0.087	1	1	2	4	3	0	9	8	0.095	1.095	1	0.75	2
**35**	135	4	0.111	0	0	0	4	3	0	7	5	0	1	0	1	0
**24**	140	8	0.122	1	1	2	6	4	2	6	9	0	1	0	1	0
**25**	145	9	0.122	1	1	2	6	4	2	6	9	0	1	0	1	0
**27**	175	9	0.125	1	1	2	6	4	2	7	9	0	1	0	1	0

Table S3

Values of descriptors characterized by high VIP values for OPLS model for HeLa cell line. Table is sorted with descending activity towards HeLa.

ID	HeLa IC50	CATS2D_07_DL	B09[C-O]	F09[C-O]	F08[C-O]	CATS2D_08_AL	B08[C-S]	RBN	NRS	CATS2D_06_DL
**28**	7	0	0	0	4	5	0	4	3	0
**30**	20	0	0	0	4	5	0	4	3	0
**29**	25	0	0	0	4	5	0	4	3	0
**33**	42	0	0	0	4	7	1	4	3	0
**37**	62	0	0	0	4	4	0	4	3	0
**23**	73	0	0	0	4	6	0	4	3	0
**38**	83	0	0	0	4	4	0	4	3	0
**35**	115	0	0	0	4	5	0	4	3	0
**24**	125	1	1	2	8	9	1	6	4	2
**25**	145	1	1	2	9	9	1	6	4	2
**31**	190	1	1	2	6	8	1	4	3	0
**27**	220	1	1	2	9	9	1	6	4	2

Table S4

Values of descriptors characterized by high VIP values for OPLS model for MCF-7 cell line. Table is sorted with descending activity towards MCF-7.

ID	MCF-7	nO	O-060	T(O..O)	B01[C-O]	B04[O-S]	B05[O-O]	B09[N-O]	B10[C-O]	F04[O-S]	F05[O-O]	F09[N-O]	F10[C-O]	B03[N-O]
**28**	4.5	2	0	2	0	0	0	0	0	0	0	0	0	0
**33**	23	2	0	2	0	0	0	0	0	0	0	0	0	0
**30**	50	2	0	2	0	0	0	0	0	0	0	0	0	0
**37**	68	2	0	2	0	0	0	0	0	0	0	0	0	0
**23**	82	2	0	2	0	0	0	0	0	0	0	0	0	0
**29**	93	2	0	2	0	0	0	0	0	0	0	0	0	0
**38**	135	2	0	2	0	0	0	0	0	0	0	0	0	0
**24**	140	2	0	2	0	0	0	0	0	0	0	0	0	0
**35**	140	2	0	2	0	0	0	0	0	0	0	0	0	0
**32**	170	2	0	2	0	0	0	0	0	0	0	0	0	1
**25**	180	3	1	12	1	1	1	1	1	1	2	1	2	1
**31**	220	2	0	2	0	0	0	0	0	0	0	0	0	0
**34**	275	3	1	12	1	1	1	1	1	1	2	1	2	1
**27**	320	3	1	12	1	1	1	1	1	1	2	1	2	1

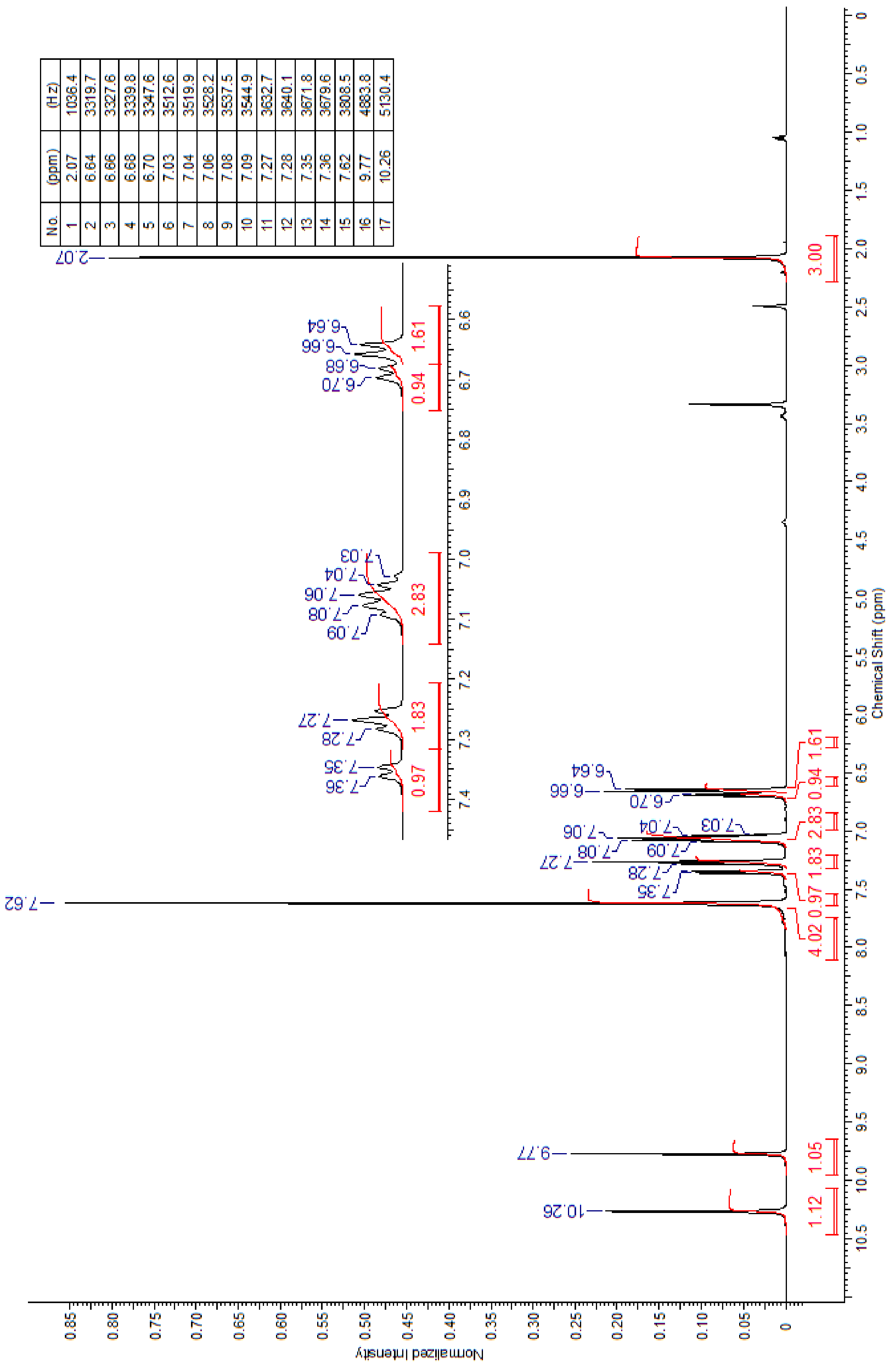

Spectrum 1. ^{1}H NMR of compd **3** (500 MHz, DMSO-d_6).

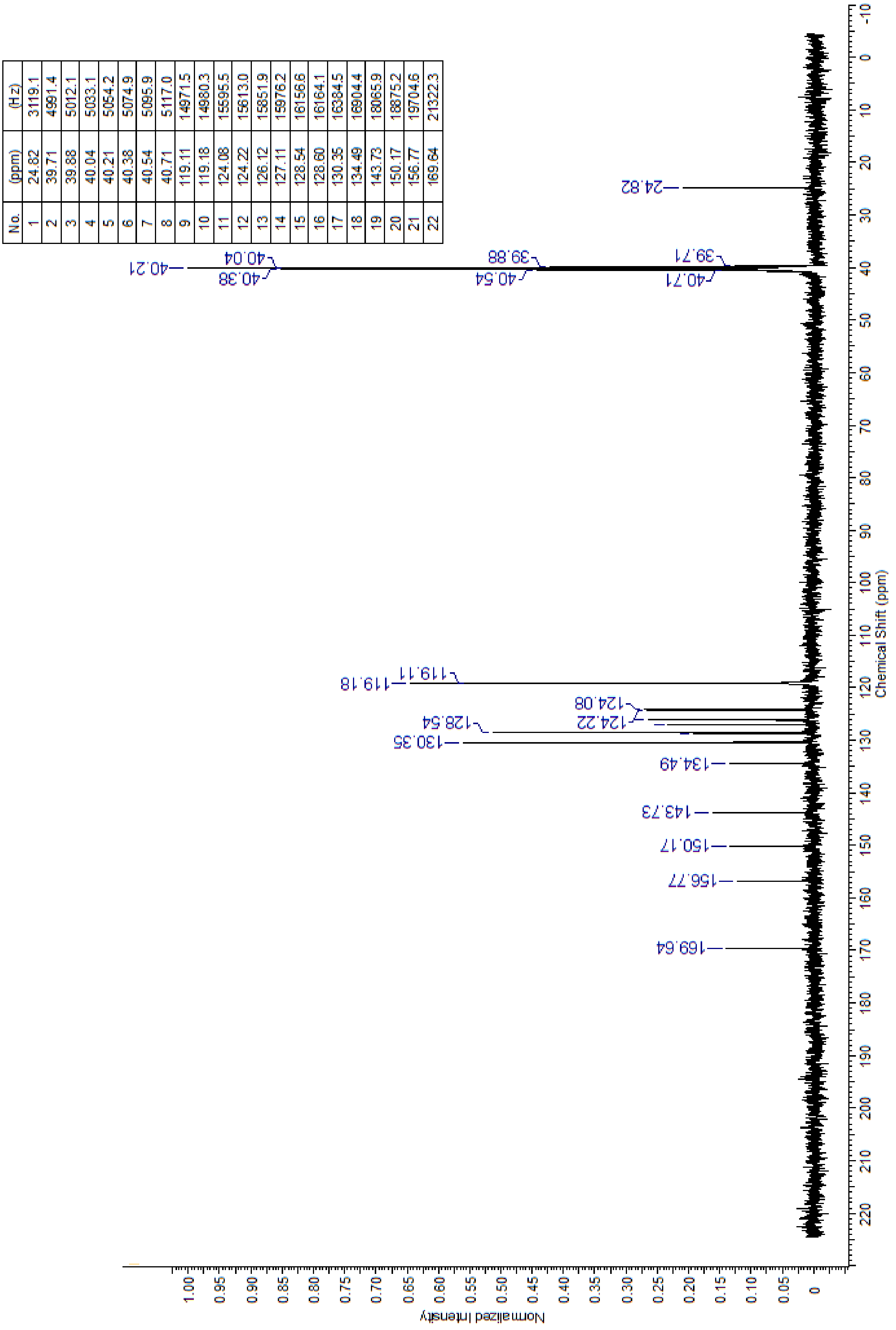

Spectrum 2. ^{13}C NMR of compd **3** (125 MHz, DMSO-d_6).

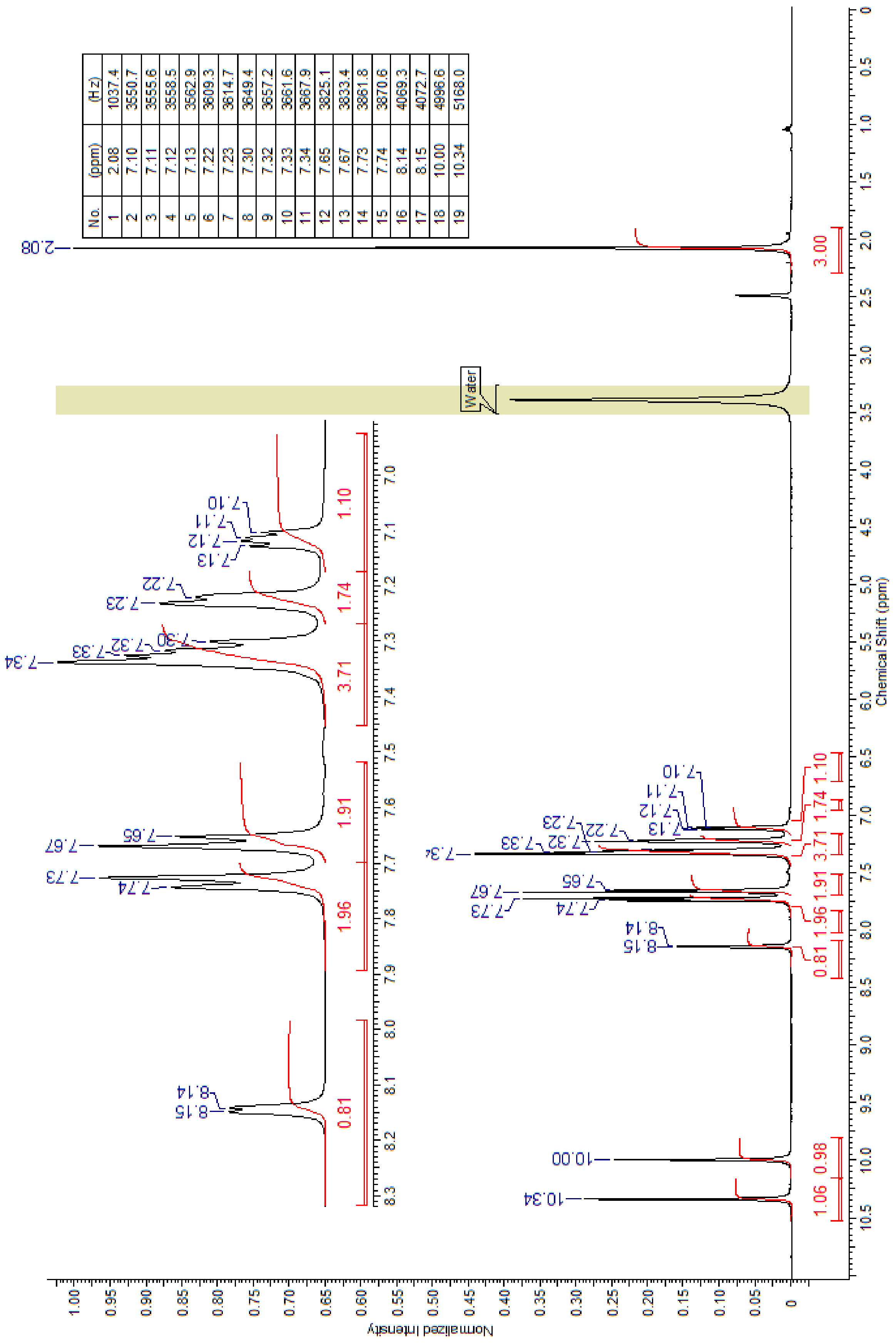

Spectrum 3. ^{1}H NMR of compd **4** (500 MHz, DMSO-d_6).

No.	(ppm)	(Hz)
1	24.84	3121.7
2	39.68	4987.5
3	39.84	5008.1
4	40.01	5029.2
5	40.18	5050.3
6	40.34	5070.9
7	40.52	5092.4
8	40.68	5113.1
9	119.21	14984.2
10	121.76	15304.3
11	128.81	16190.0
12	129.10	16227.3
13	129.70	16302.0
14	130.87	16448.6
15	131.35	16509.2
16	134.41	16894.3
17	135.16	16988.7
18	135.32	17008.1
19	144.01	18100.6
20	148.27	18636.3
21	156.92	19724.0
22	169.74	21335.0

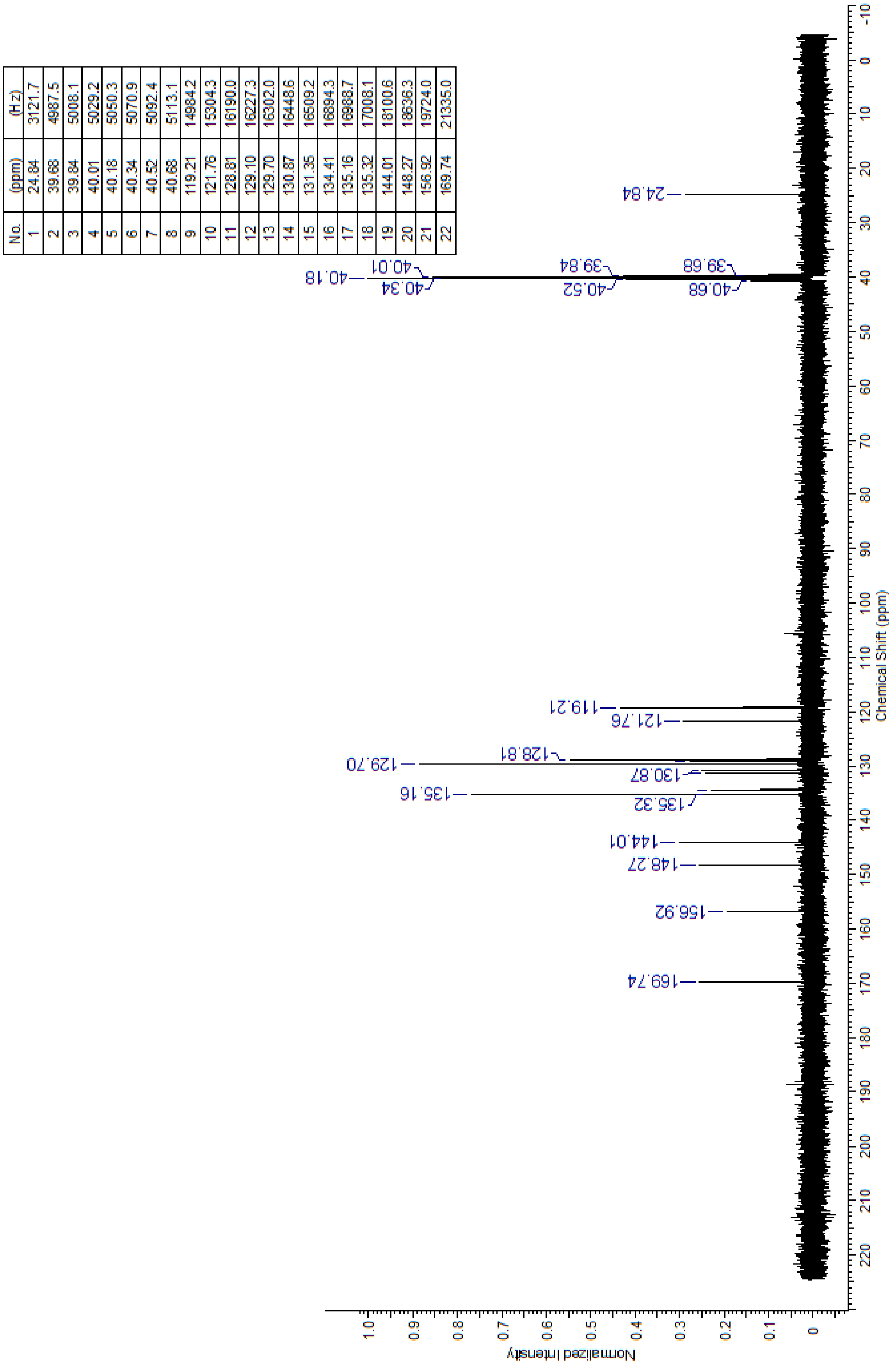

Spectrum 4. ^{13}C NMR of compd **4** (125 MHz, DMSO-d_6).

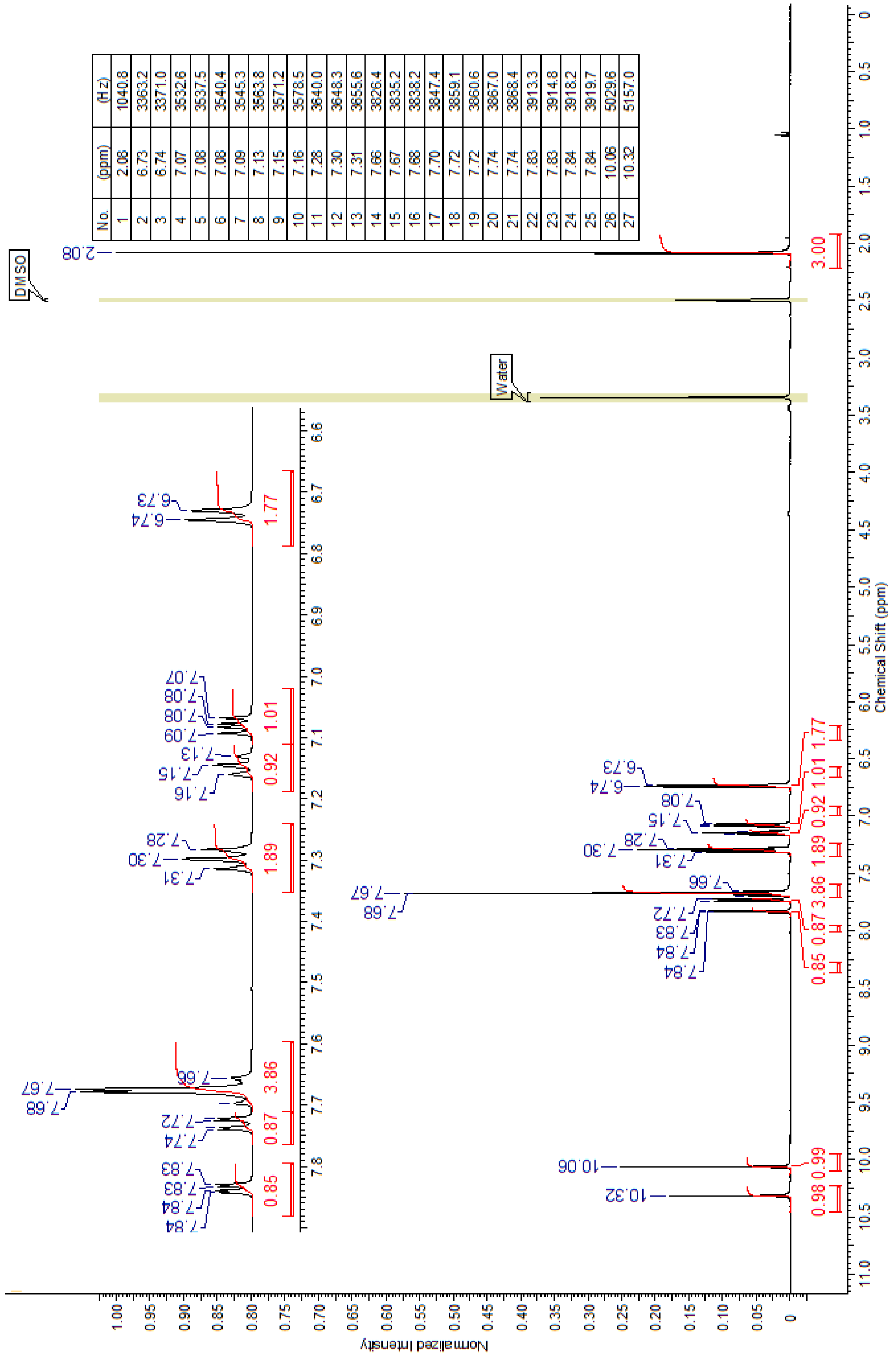

No.	(ppm)	(Hz)
1	2.08	1040.8
2	6.73	3363.2
3	6.74	3371.0
4	7.07	3532.6
5	7.08	3537.5
6	7.08	3540.4
7	7.09	3545.3
8	7.13	3563.8
9	7.15	3571.2
10	7.16	3578.5
11	7.28	3640.0
12	7.30	3648.3
13	7.31	3655.6
14	7.66	3826.4
15	7.67	3835.2
16	7.68	3838.2
17	7.70	3847.4
18	7.72	3859.1
19	7.72	3860.6
20	7.74	3867.0
21	7.74	3868.4
22	7.83	3913.3
23	7.83	3914.8
24	7.84	3918.2
25	7.84	3919.7
26	10.06	5029.6
27	10.32	5157.0

Spectrum 5. ^{1}H NMR of compd **5** (500 MHz, DMSO-d_6).

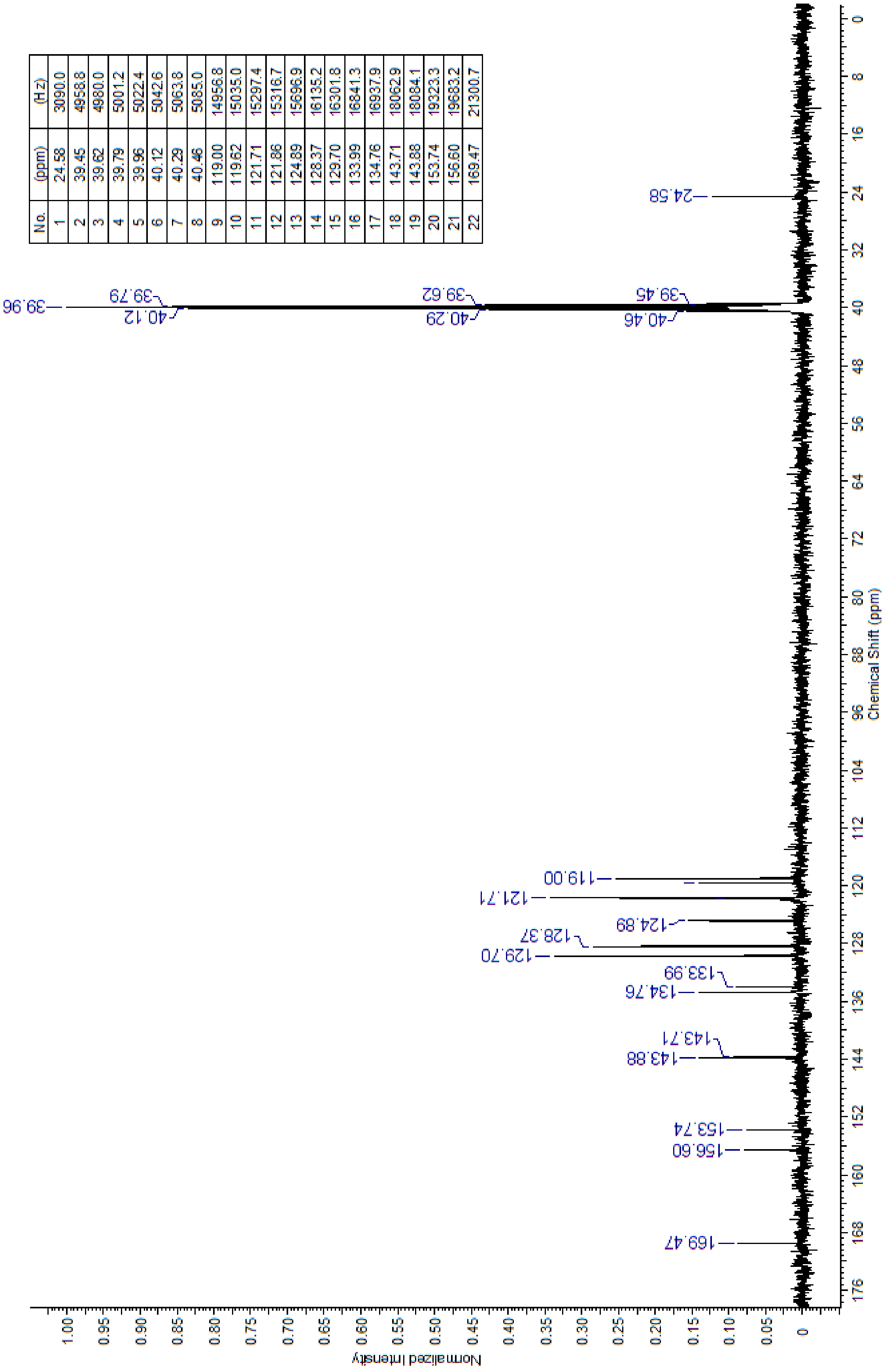

No.	(ppm)	(Hz)
1	24.58	3090.0
2	39.45	4958.8
3	39.62	4980.0
4	39.79	5001.2
5	39.96	5022.4
6	40.12	5042.6
7	40.29	5063.8
8	40.46	5085.0
9	119.00	14956.8
10	119.62	15035.0
11	121.71	15297.4
12	121.86	15316.7
13	124.89	15696.9
14	128.37	16135.2
15	129.70	16301.8
16	133.99	16841.3
17	134.76	16937.9
18	143.71	18062.9
19	143.88	18084.1
20	153.74	19323.3
21	156.60	19683.2
22	169.47	21300.7

Spectrum 6. ^{13}C NMR of compd **5** (125 MHz, DMSO-d_6).

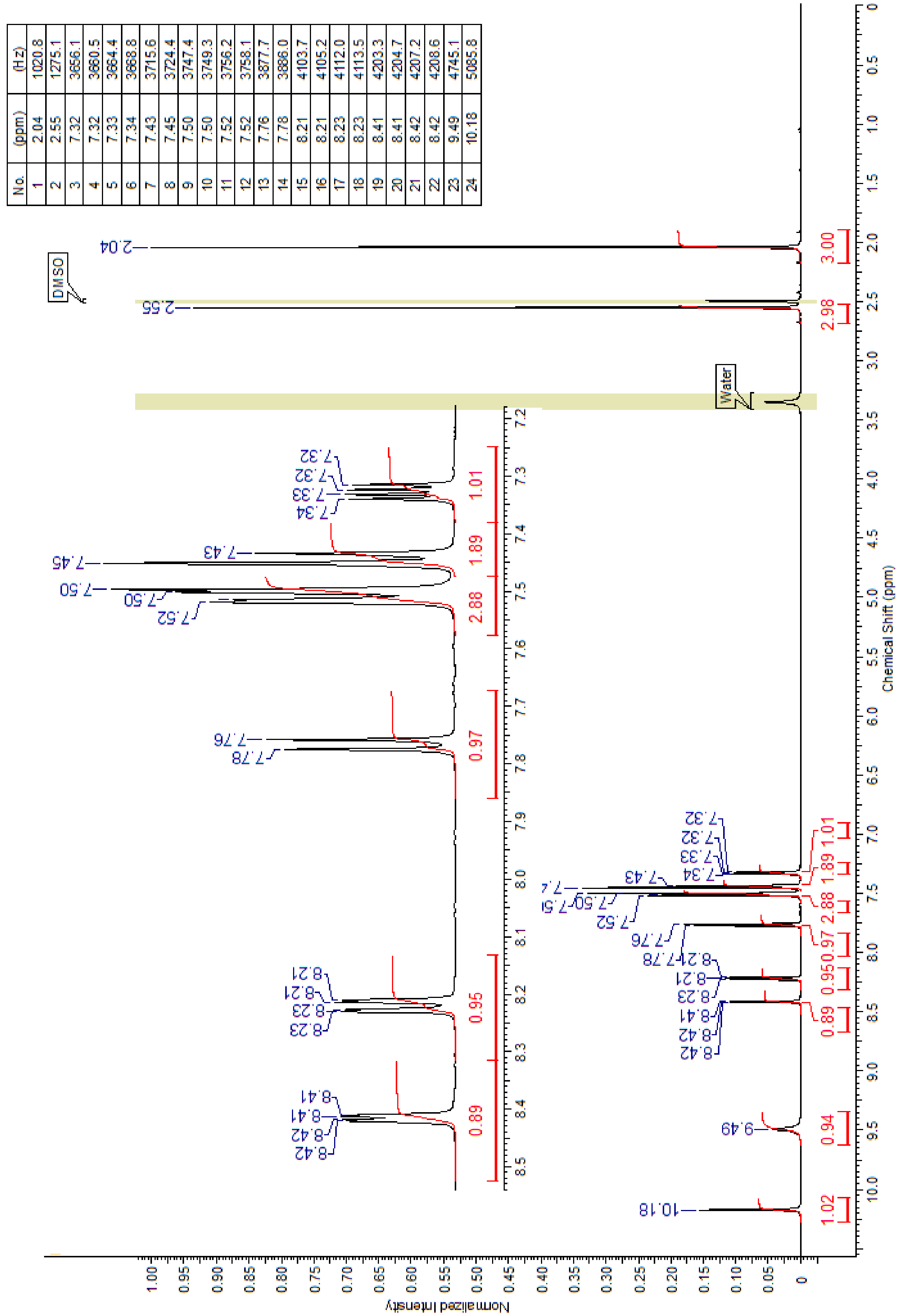

No.	(ppm)	(Hz)
1	2.04	1020.8
2	2.55	1275.1
3	7.32	3656.1
4	7.32	3660.5
5	7.33	3664.4
6	7.34	3668.8
7	7.43	3715.6
8	7.45	3724.4
9	7.50	3747.4
10	7.50	3749.3
11	7.52	3756.2
12	7.52	3758.1
13	7.76	3877.7
14	7.78	3886.0
15	8.21	4103.7
16	8.21	4105.2
17	8.23	4112.0
18	8.23	4113.5
19	8.41	4203.3
20	8.41	4204.7
21	8.42	4207.2
22	8.42	4208.6
23	9.49	4745.1
24	10.18	5085.8

Spectrum 7. ^{1}H NMR of compd **8** (500 MHz, DMSO-d_6).

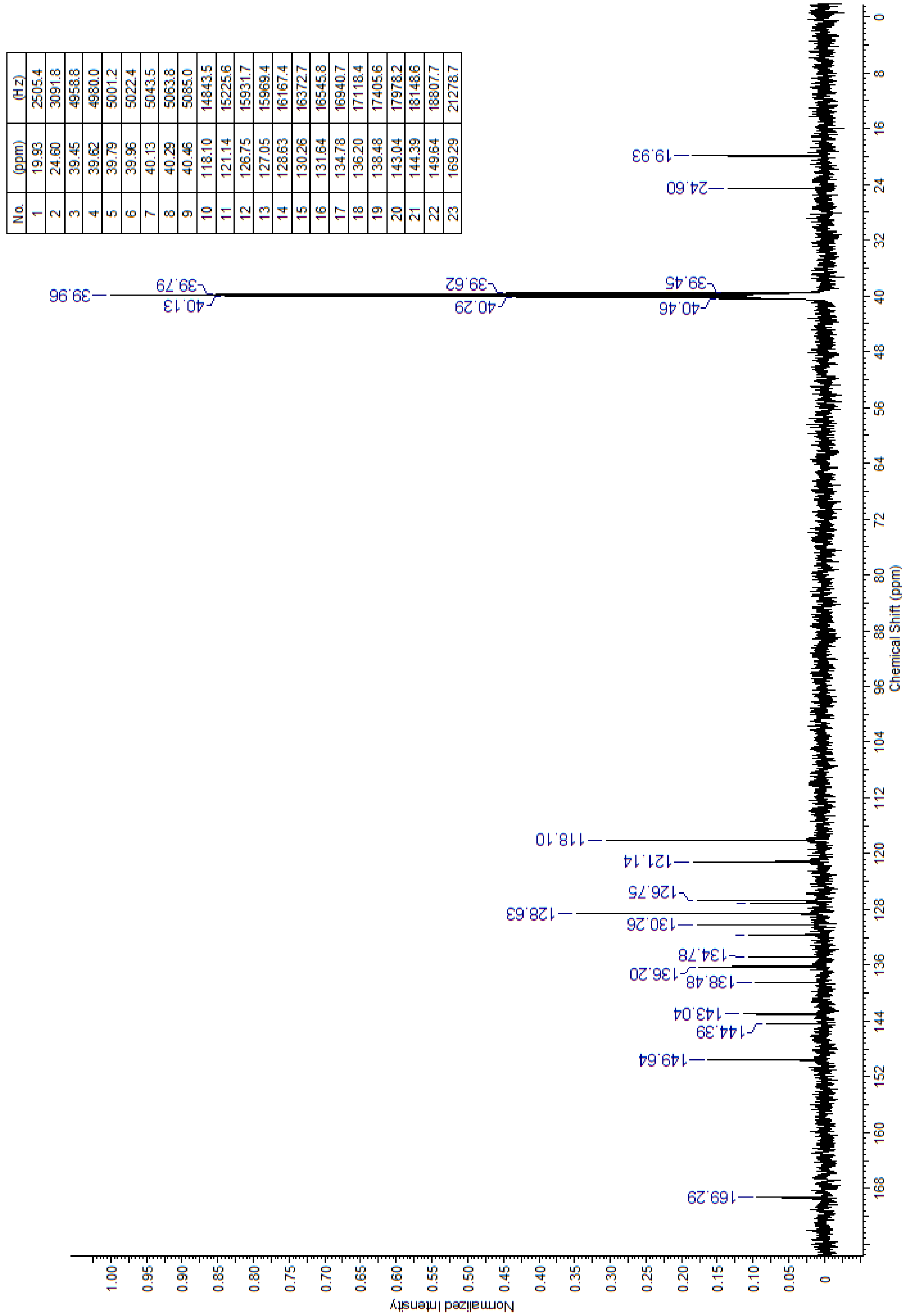

No.	(ppm)	(Hz)
1	19.93	2505.4
2	24.60	3091.8
3	39.45	4958.8
4	39.62	4980.0
5	39.79	5001.2
6	39.96	5022.4
7	40.13	5043.5
8	40.29	5063.8
9	40.46	5085.0
10	118.10	14843.5
11	121.14	15225.6
12	126.75	15931.7
13	127.05	15969.4
14	128.63	16167.4
15	130.26	16372.7
16	131.64	16545.8
17	134.78	16940.7
18	136.20	17118.4
19	138.48	17405.6
20	143.04	17978.2
21	144.39	18148.6
22	149.64	18807.7
23	169.29	21278.7

Spectrum 8. ^{13}C NMR of compd **8** (125 MHz, DMSO-d_6).

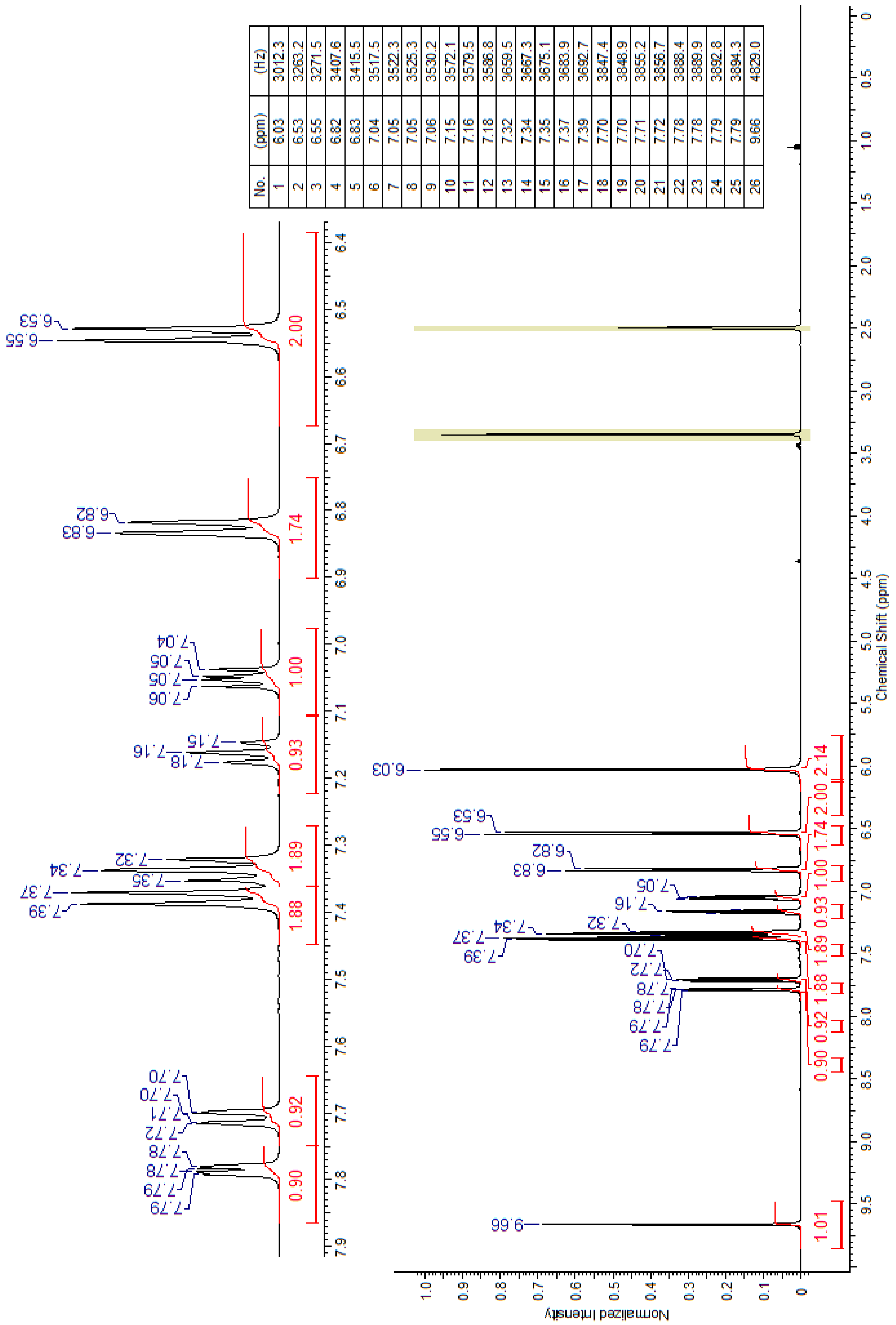

No.	(ppm)	(Hz)
1	6.03	3012.3
2	6.53	3263.2
3	6.55	3271.5
4	6.82	3407.6
5	6.83	3415.5
6	7.04	3517.5
7	7.05	3522.3
8	7.05	3525.3
9	7.06	3530.2
10	7.15	3572.1
11	7.16	3579.5
12	7.18	3586.8
13	7.32	3659.5
14	7.34	3667.3
15	7.35	3675.1
16	7.37	3683.9
17	7.39	3692.7
18	7.70	3847.4
19	7.70	3848.9
20	7.71	3855.2
21	7.72	3856.7
22	7.78	3888.4
23	7.78	3889.9
24	7.79	3892.8
25	7.79	3894.3
26	9.66	4829.0

Spectrum 9. ^{1}H NMR of compd **16** (500 MHz, DMSO-d_6).

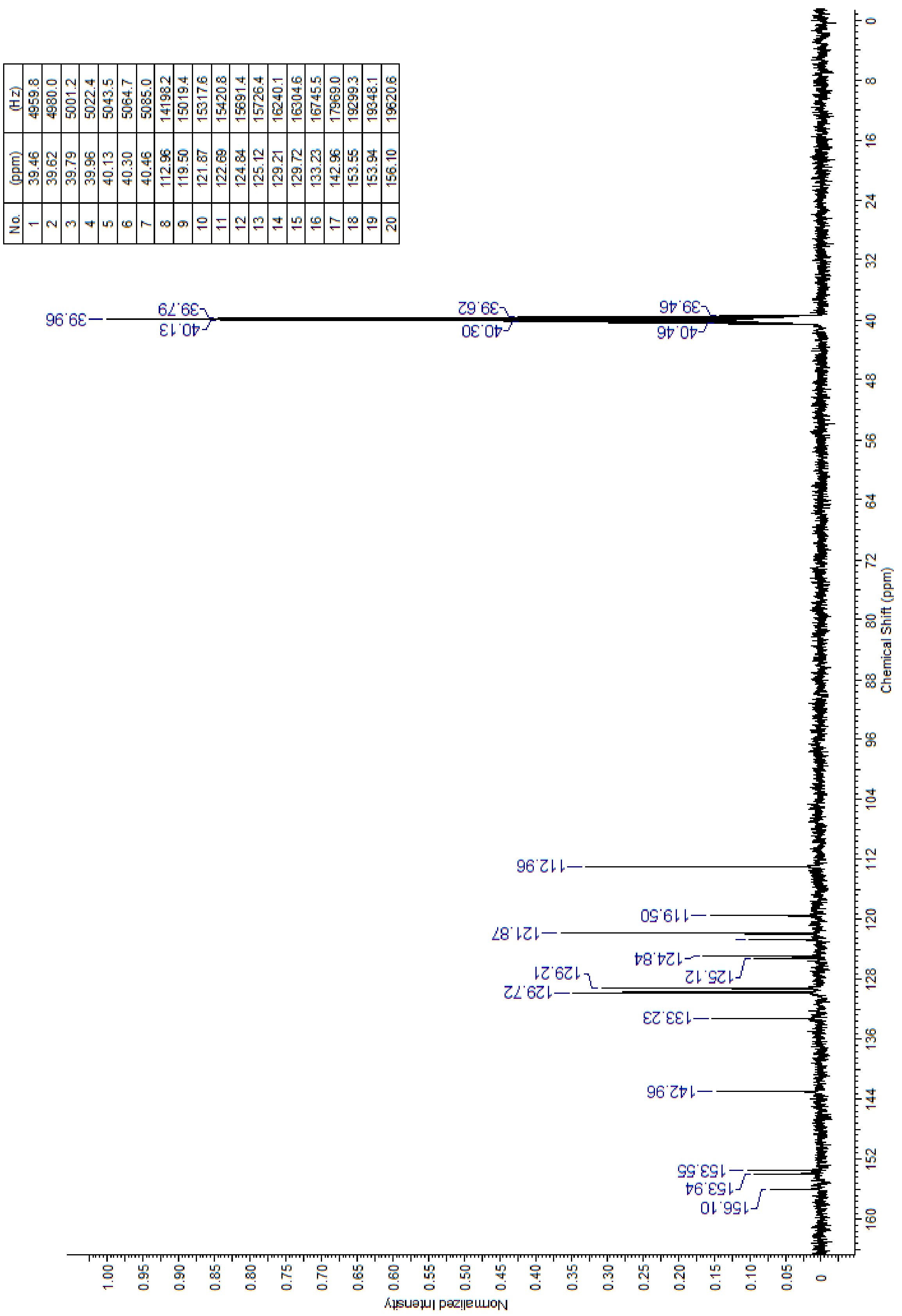

Spectrum 10. ^{13}C NMR of compd **16** (125 MHz, DMSO-d_6).

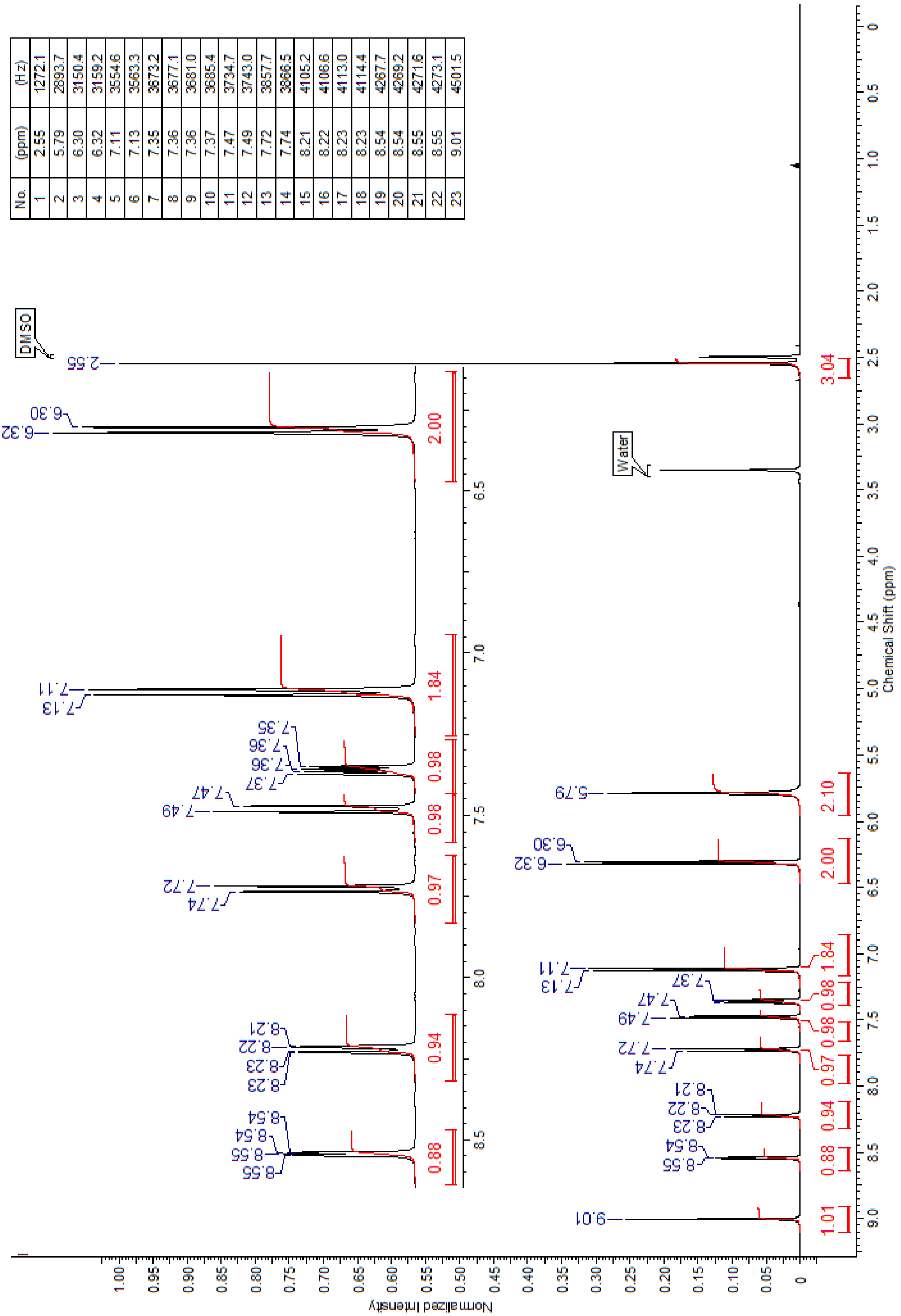

No.	(ppm)	(Hz)
1	2.55	1272.1
2	5.79	2893.7
3	6.30	3150.4
4	6.32	3159.2
5	7.11	3554.6
6	7.13	3563.3
7	7.35	3673.2
8	7.36	3677.1
9	7.36	3681.0
10	7.37	3685.4
11	7.47	3734.7
12	7.49	3743.0
13	7.72	3857.7
14	7.74	3866.5
15	8.21	4105.2
16	8.22	4106.6
17	8.23	4113.0
18	8.23	4114.4
19	8.54	4267.7
20	8.54	4269.2
21	8.55	4271.6
22	8.55	4273.1
23	9.01	4501.5

Spectrum 11. ^{1}H NMR of compd **19** (500 MHz, DMSO-d_6).

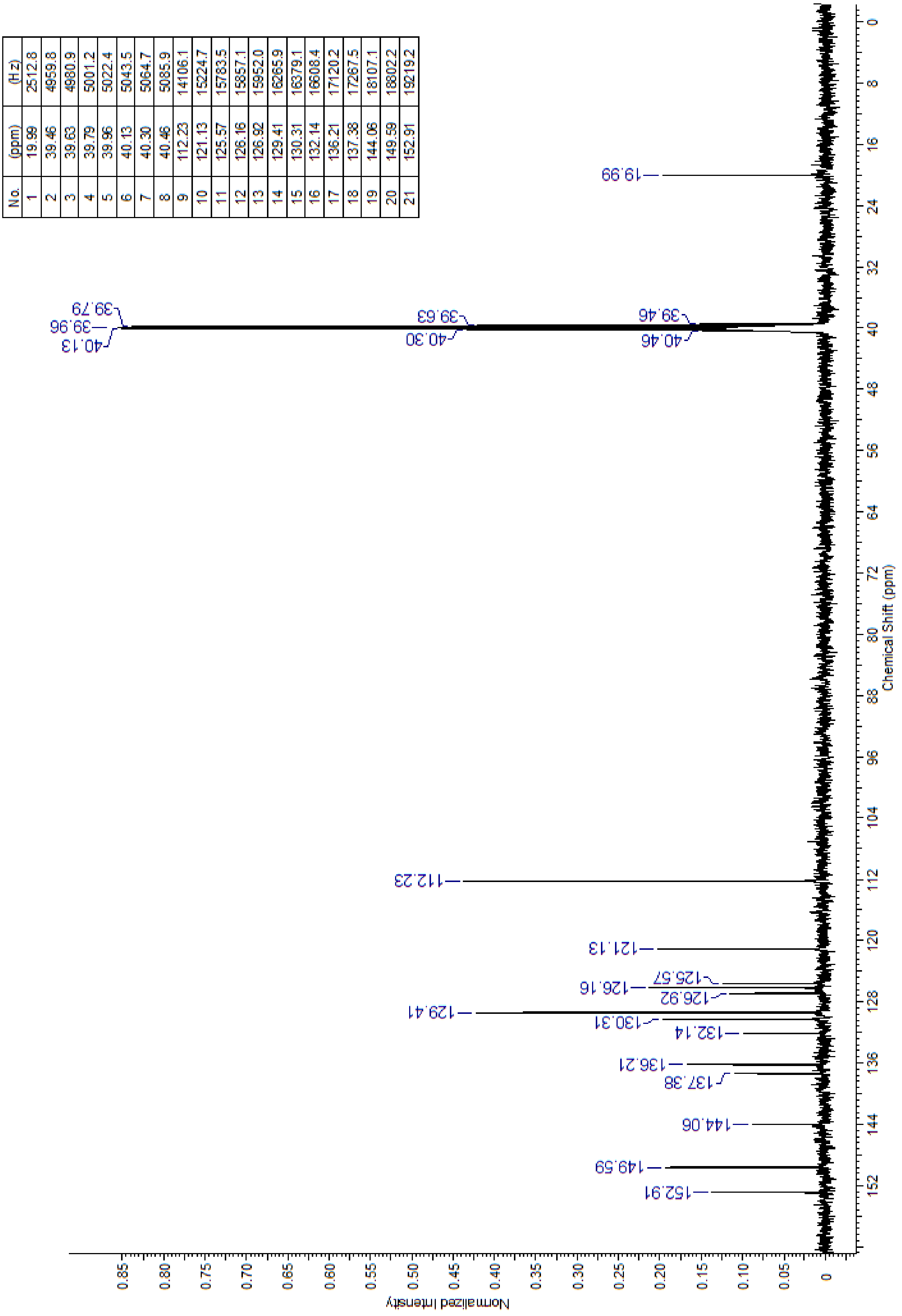

No.	(ppm)	(Hz)
1	19.99	2512.8
2	39.46	4959.8
3	39.63	4980.9
4	39.79	5001.2
5	39.96	5022.4
6	40.13	5043.5
7	40.30	5064.7
8	40.46	5085.9
9	112.23	14106.1
10	121.13	15224.7
11	125.57	15783.5
12	126.16	15857.1
13	126.92	15952.0
14	129.41	16265.9
15	130.31	16379.1
16	132.14	16608.4
17	136.21	17120.2
18	137.38	17267.5
19	144.06	18107.1
20	149.59	18802.2
21	152.91	19219.2

Spectrum 12. ^{13}C NMR of compd **19** (125 MHz, DMSO-d_6).

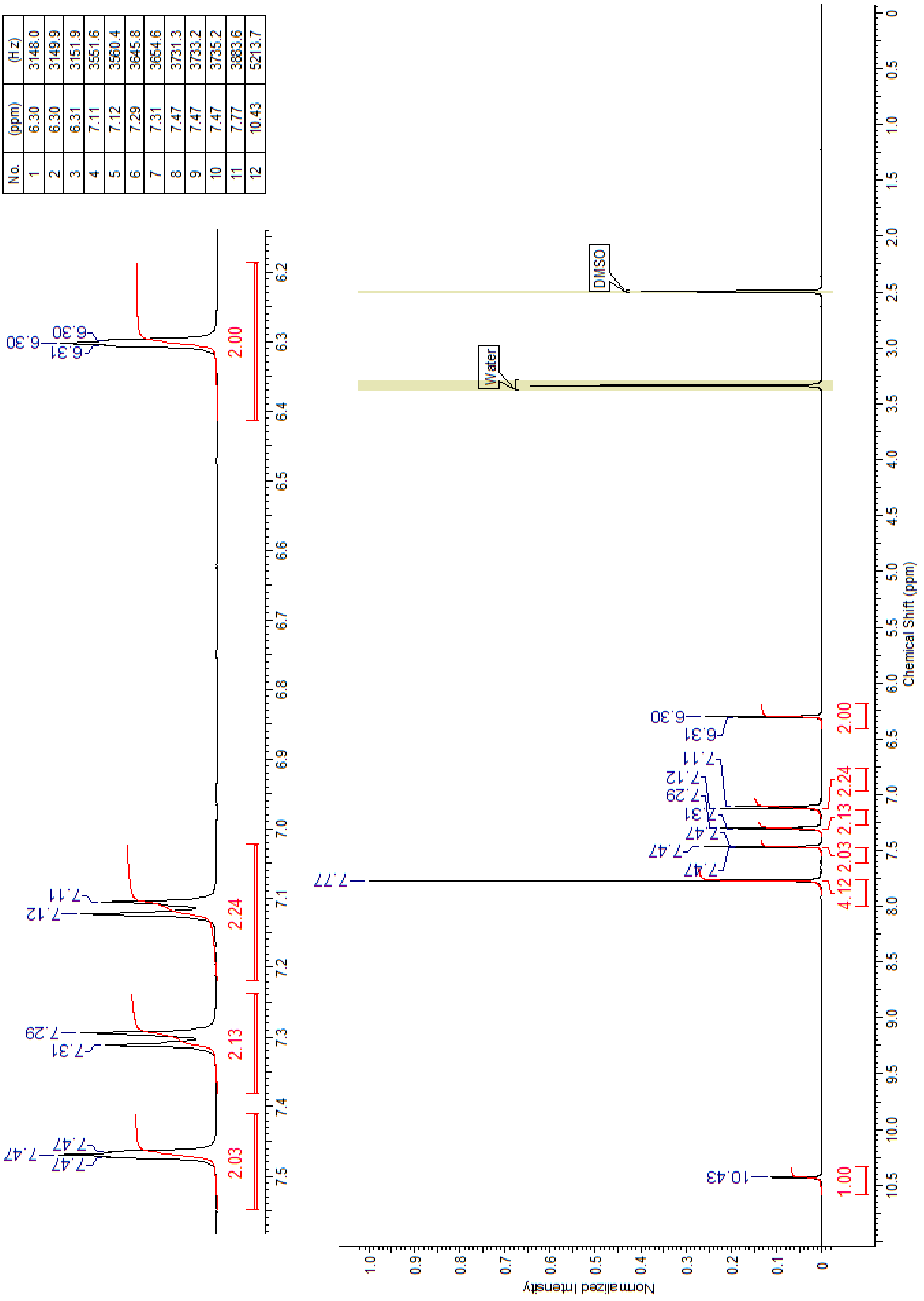

No.	(ppm)	(Hz)
1	6.30	3148.0
2	6.30	3149.9
3	6.31	3151.9
4	7.11	3551.6
5	7.12	3560.4
6	7.29	3645.8
7	7.31	3654.6
8	7.47	3731.3
9	7.47	3733.2
10	7.47	3735.2
11	7.77	3883.6
12	10.43	5213.7

Spectrum 13. ^{1}H NMR of compd **23** (500 MHz, DMSO-d_6).

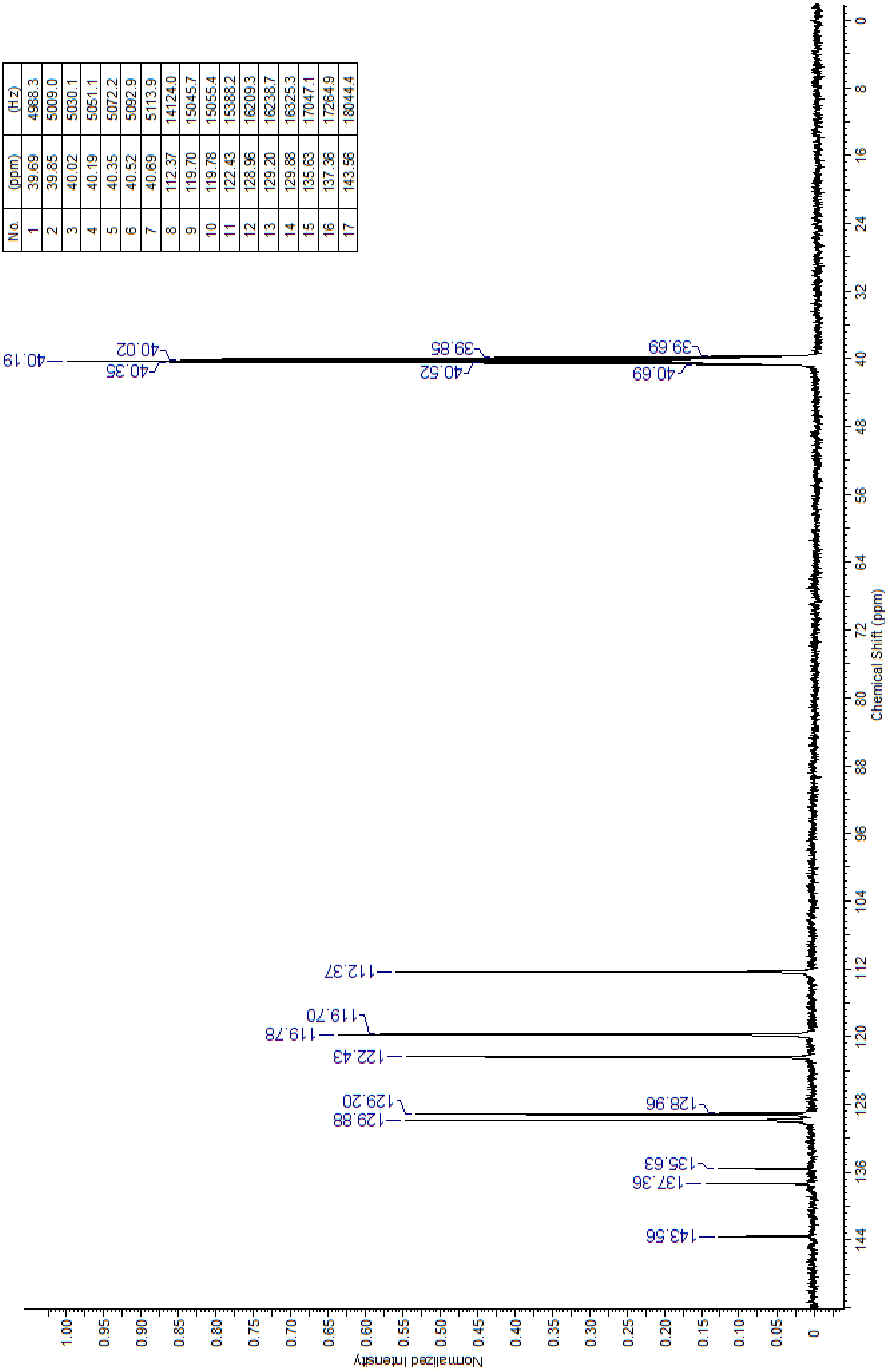

No.	(ppm)	(Hz)
1	39.69	4988.3
2	39.85	5009.0
3	40.02	5030.1
4	40.19	5051.1
5	40.35	5072.2
6	40.52	5092.9
7	40.69	5113.9
8	112.37	14124.0
9	119.70	15045.7
10	119.78	15055.4
11	122.43	15388.2
12	128.96	16209.3
13	129.20	16238.7
14	129.88	16325.3
15	135.63	17047.1
16	137.36	17264.9
17	143.56	18044.4

Spectrum 14. ^{13}C NMR of compd **23** (125 MHz, DMSO-d_6).

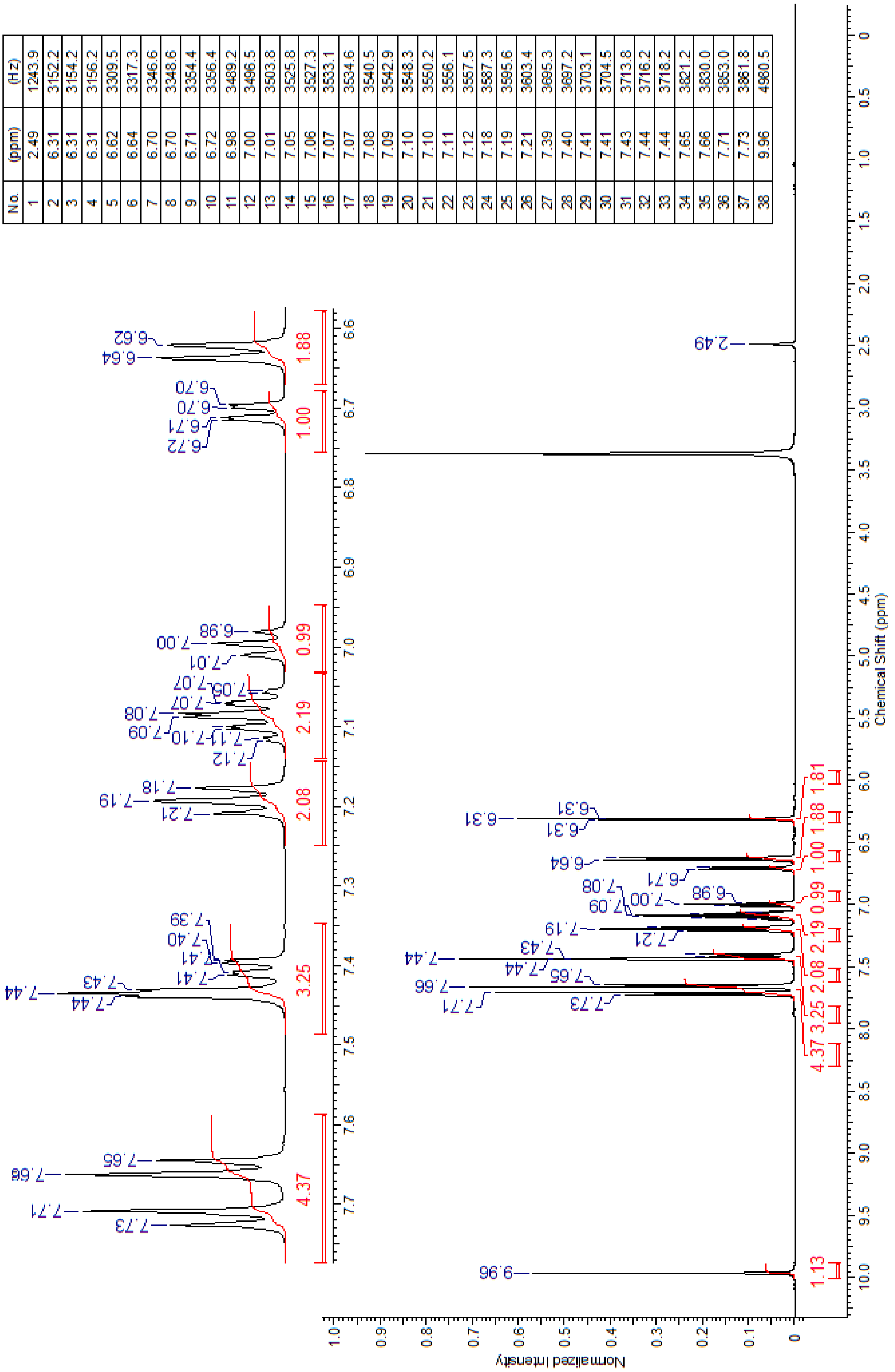

Spectrum 15. ^{1}H NMR of compd **25** (500 MHz, DMSO-d_6).

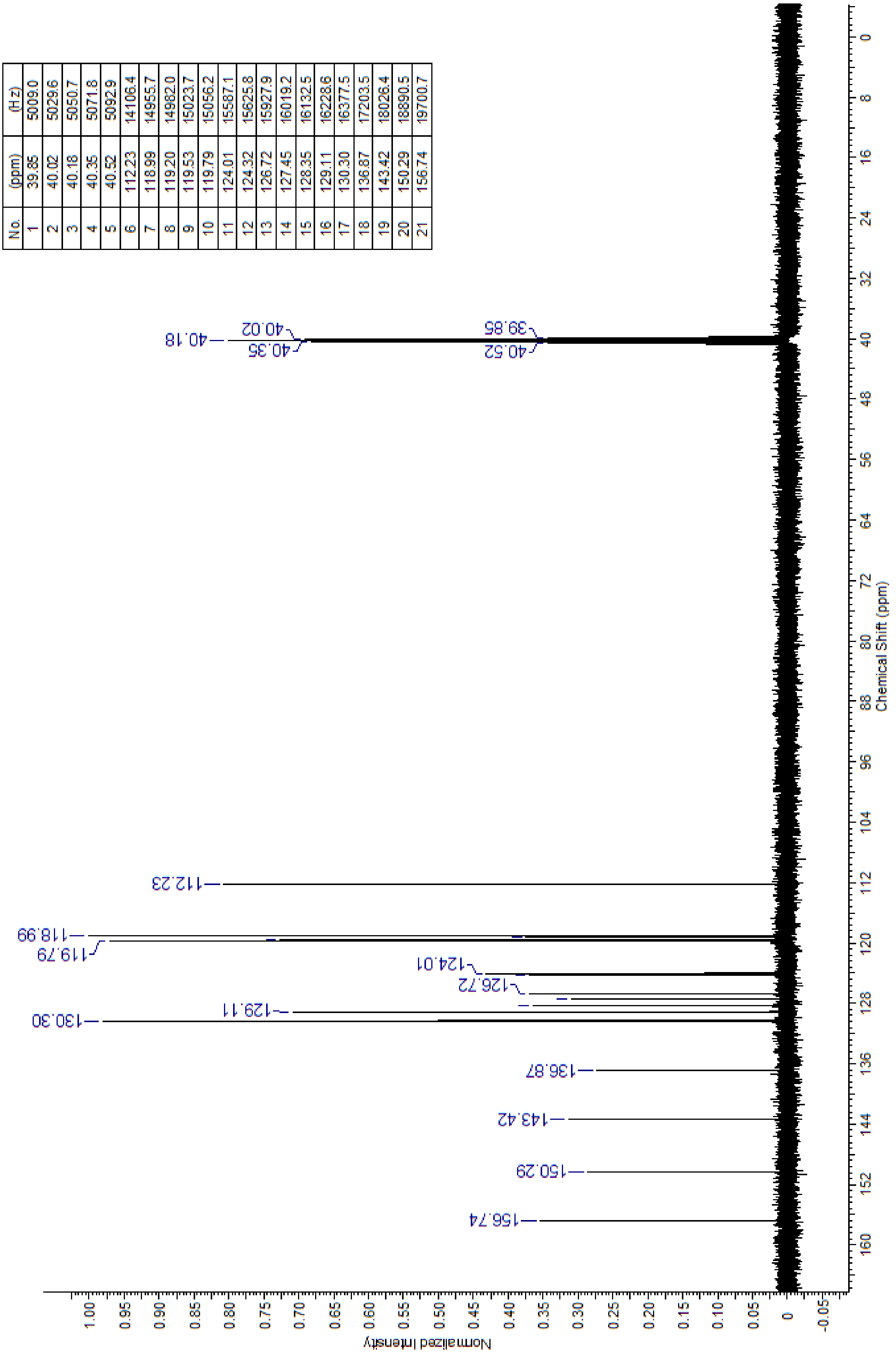

No.	(ppm)	(Hz)
1	39.85	5009.0
2	40.02	5029.6
3	40.18	5050.7
4	40.35	5071.8
5	40.52	5092.9
6	112.23	14106.4
7	118.99	14955.7
8	119.20	14982.0
9	119.53	15023.7
10	119.79	15056.2
11	124.01	15587.1
12	124.32	15625.8
13	126.72	15927.9
14	127.45	16019.2
15	128.35	16132.5
16	129.11	16228.6
17	130.30	16377.5
18	136.87	17203.5
19	143.42	18026.4
20	150.29	18890.5
21	156.74	19700.7

Spectrum 16. ^{13}C NMR of compd **25** (125 MHz, DMSO-d_6).

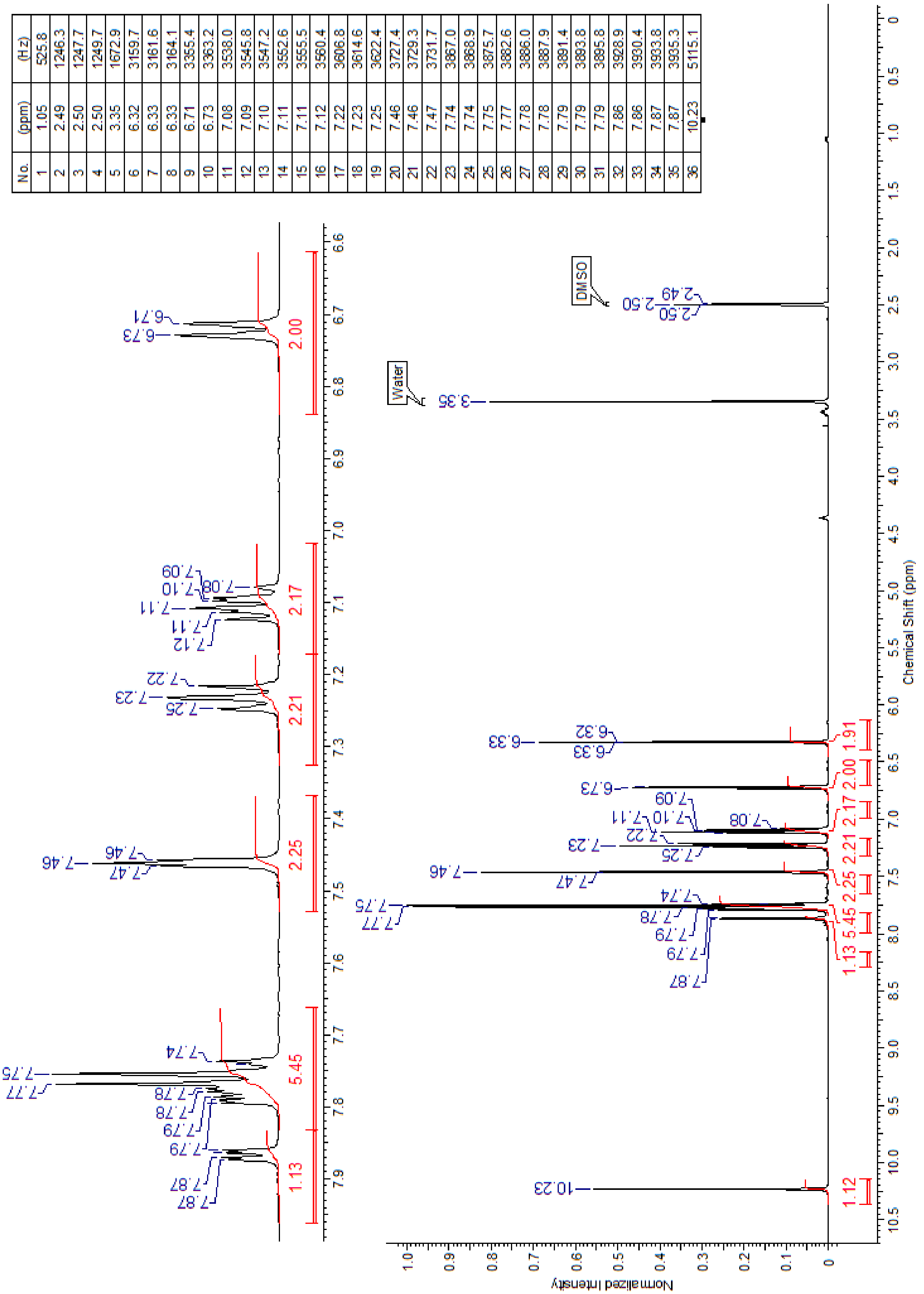

Spectrum 17. ^{1}H NMR of compd **27** (500 MHz, DMSO-d_6).

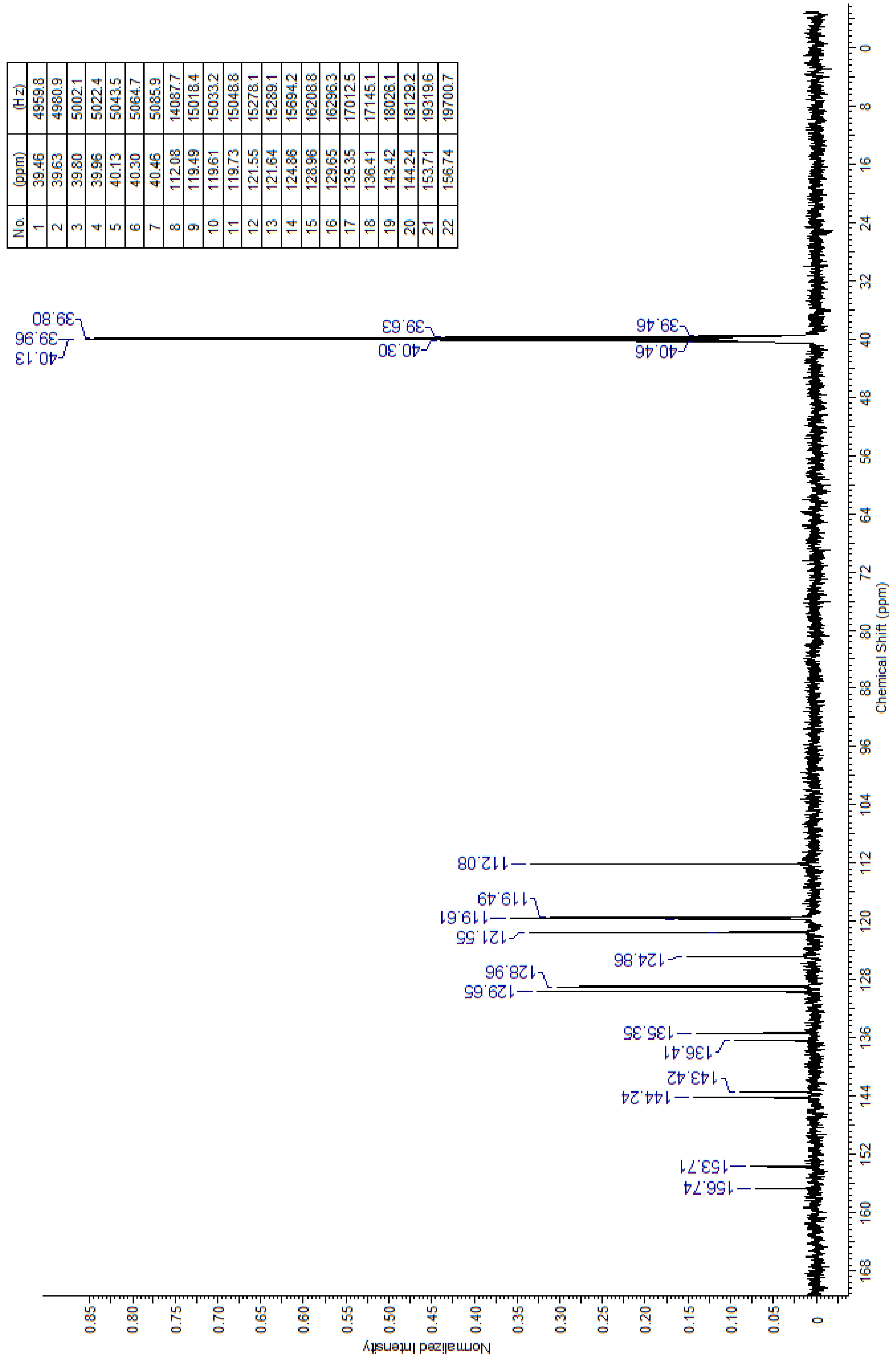

Spectrum 18. ^{13}C NMR of compd **27** (125 MHz, DMSO-d_6).

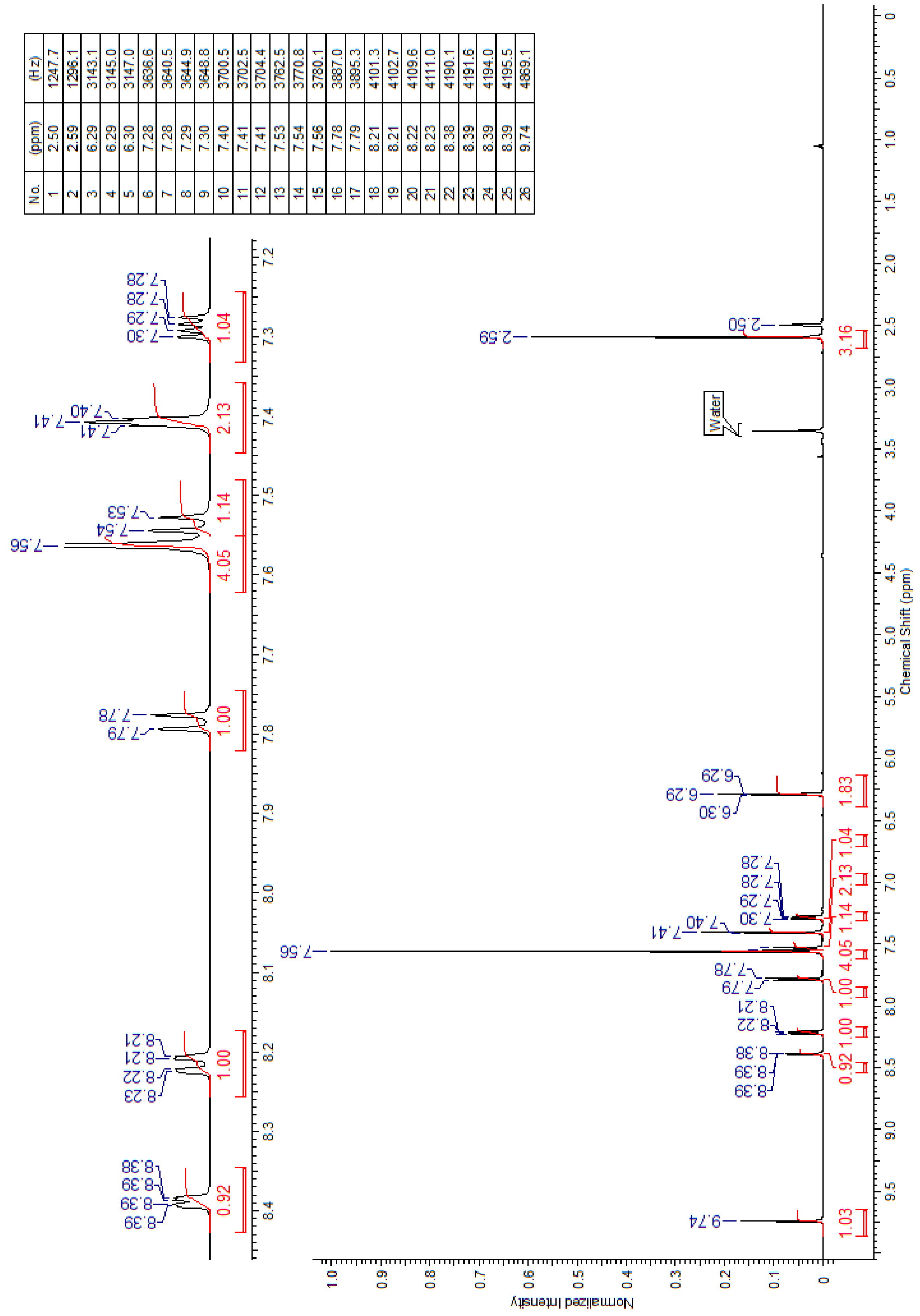

No.	(ppm)	(Hz)
1	2.50	1247.7
2	2.59	1296.1
3	6.29	3143.1
4	6.29	3145.0
5	6.30	3147.0
6	7.28	3636.6
7	7.28	3640.5
8	7.29	3644.9
9	7.30	3648.8
10	7.40	3700.5
11	7.41	3702.5
12	7.41	3704.4
13	7.53	3762.5
14	7.54	3770.8
15	7.56	3780.1
16	7.78	3887.0
17	7.79	3895.3
18	8.21	4101.3
19	8.21	4102.7
20	8.22	4109.6
21	8.23	4111.0
22	8.38	4190.1
23	8.39	4191.6
24	8.39	4194.0
25	8.39	4195.5
26	9.74	4869.1

Spectrum 19. ^{1}H NMR of compd **30** (500 MHz, DMSO-d_6).

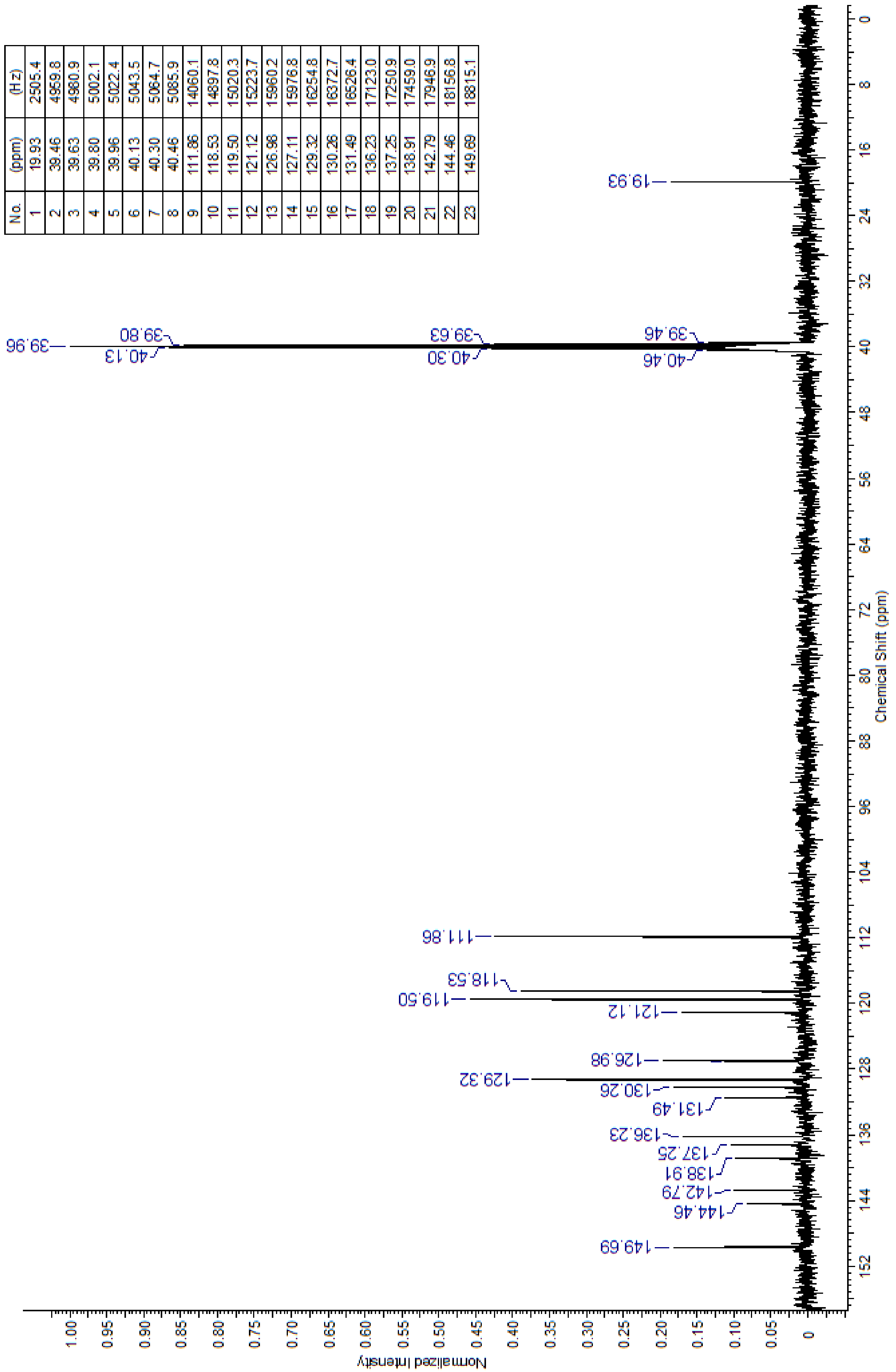

Spectrum 20. ^{13}C NMR of compd **30** (125 MHz, DMSO-d_6).
